# Catalyst-Free Assembly of δ-Lactam-Based Hydrazide–Hydrazone Compounds from 3-Arylglutaconic Anhydrides and Aldazines

**DOI:** 10.3390/ijms26188834

**Published:** 2025-09-10

**Authors:** Anna Ananeva, Elizaveta Karchuganova, Dar’ya Spiridonova, Grigory Kantin, Olga Bakulina

**Affiliations:** Department of Medicinal Chemistry, Institute of Chemistry, Saint Petersburg State University, 199034 Saint Petersburg, Russia; ananjewa.anna98@yandex.ru (A.A.); exoeliza@gmail.com (E.K.); daria.spiridonova@spbu.ru (D.S.); kanting@mail.ru (G.K.)

**Keywords:** hydrazide–hydrazone, aldazine, anhydride, arylglutaconic, δ-lactam, annulation

## Abstract

A novel general approach to cyclic hydrazide–hydrazone compounds with a dihydropyridine-2-one core has been developed, involving annulation of symmetrical aldazines with 3-arylglutaconic anhydrides. This approach provides the benefits of straightforward and catalyst-free procedures, diastereoselectivity, and the ability to switch between two isomeric dihydropyridine-2-one cores based on the reaction temperature. Several post-modifications were performed on the side functional groups and the core to demonstrate the synthetic potential of the resulting products. This approach significantly expands the chemical diversity of medicinally relevant *N*-functionalized δ-lactams.

## 1. Introduction

**Figure 1:**
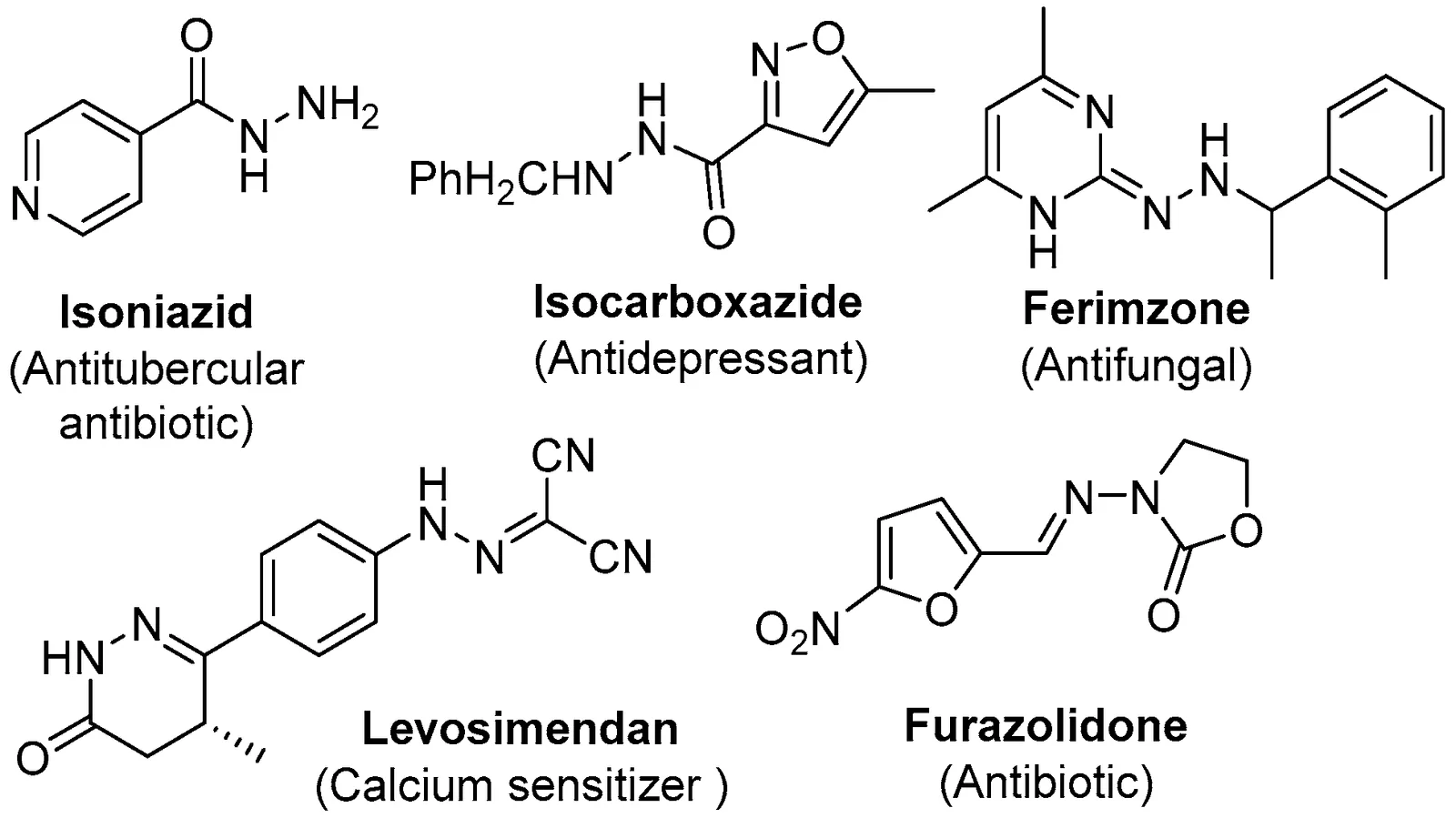
Structures of commercial drugs containing hydrazide, hydrazone, or hydrazide–hydrazone moieties.

Acyl hydrazides [1] (R^1^CONR^2^NR^3^R^4^) and hydrazones [2,3] (R^1^R^2^C=NR^3^NR^4^R^5^) play a crucial role in modern drug development due to their success, with more than 50 years of extensive clinical use (Figure 1) as antibacterial and antifungal agents, anticonvulsants, and antidepressants. Recent developments have expanded their applications to include the treatment of cancer [4,5,6], chronic inflammatory diseases [7,8], neurodegenerative diseases, and viral infections [9,10]. These two structural motifs are often combined into a single functional group, and such compounds are then referred to as hydrazide–hydrazone [11,12,13,14] derivatives. Their structure often enhances drug delivery (e.g., to tumor sites) and activity by affecting solubility, stability, and toxicity. The hydrazone moiety undergoes hydrolysis under physiological conditions to produce less toxic metabolites, making the hydrazide–hydrazone functional group a promising platform for prodrug design [14]. Their sensitivity to acid-promoted hydrolysis specifically provides hydrazones with the unique ability to release the attached pharmacophore at the tumor cells having acidic pH, enabling pH-responsive targeted drug delivery [15]. Furthermore, hydrazide–hydrazone derivatives have been used in molecular switches, sensing, metal complexation [12], and the synthesis of heterocyclic compounds (e.g., β-lactams [16]). These practical applications make improving the structural diversity and synthetic accessibility of hydrazide–hydrazone derivatives highly desirable in medicinal chemistry and organic synthesis.

Although acyclic derivatives are readily available, there are relatively few known methods for preparing the corresponding cyclic compounds. One of the most interesting of these is the δ-lactam scaffold, which is considered privileged [17] in drug discovery. All three possible degrees of saturation for such a scaffold, namely piperidine-2-one, pyridine-2-one and dihydropyridine-2-one, are widely spread among the structures of bioactive compounds. The methodologies for constructing dihydropyridones offer greater versatility and efficiency as they provide access to all three types of lactams mentioned above through a single annulation strategy combined with post-condensation oxidation and reduction.

**Figure 2:**
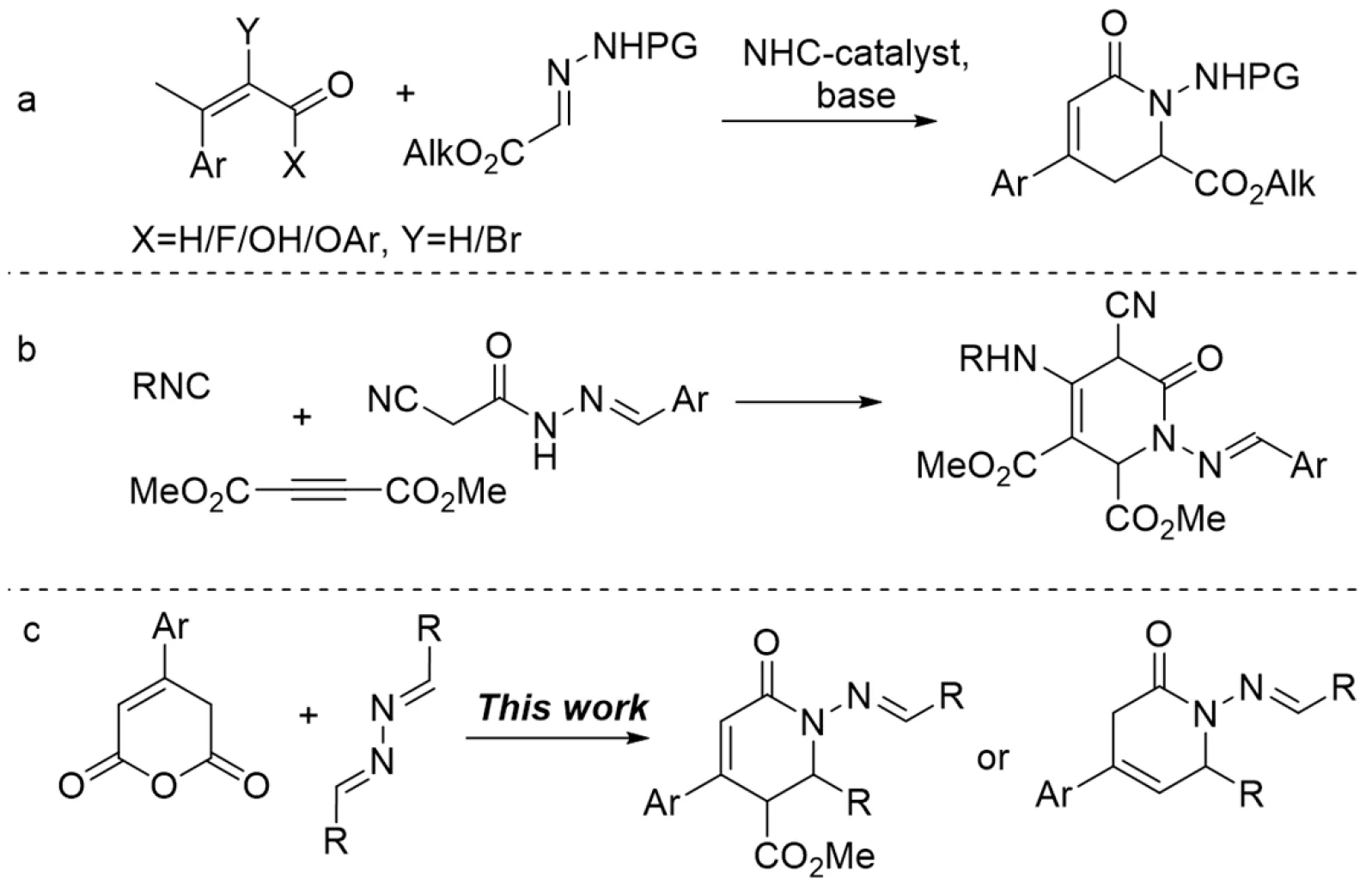
Published approaches to cyclic hydrazide–hydrazones with dihydropyridine-2-one core and the aim of the present study: (**a**) based on [4 + 2] annulation of acrolein derivatives with hydrazide–hydrazones derivatived from alkyl glyoxylate [18,19,20,21,22,23] (**b**) based on multicomponent reaction involving isocyanides, *N*-arylidene-2-cyanoacetohydrazides, and dimethylpropiolate [24] (**c**) based on reaction of cyclic anhydrides and aldazines.

Published approaches to assembling such molecules functionalized with hydrazide–hydrazone fragments include a group of similar [4 + 2] annulation substrates of the croton series (e.g., aldehydes, acyl halides, acids, and esters) with acyclic hydrazide–hydrazone derivatives from alkyl glyoxylate (Figure 2a). These reactions are often promoted by an *N*-heterocyclic carbene (NHC) catalyst [18,19,20,21,22] and can be performed in an electrocatalytic [23] format. Another approach [24] is based on a multicomponent reaction involving isocyanides, *N*-arylidene-2-cyanoacetohydrazides, and dimethylpropiolate (Figure 2b). A significant limitation of these methods is the lack of variability of the substituent at position 6—only an ester group can be present. Additionally, only one type of regioisomer can be obtained, mostly 5,6-dihydropyridin-2(1H)-ones.

In 2017, our group reported the synthesis of cyclic hydrazide–hydrazone compounds involving selective mono-annulation of aldazines with homophthalic anhydride (Castagnoli–Cushman reaction, CCR [25]), which was used to obtain compounds with the same functional group but based on a different scaffold, namely tetrahydroisoquinolone [26].

In this study, we present a novel approach to rare cyclic hydrazide–hydrazones with a dihydropyridine-2-one scaffold that employs the same type of aldazine reactivity but extends it to a new type of anhydride: 3-arylglutaconic anhydrides (Figure 2c). This methodology significantly increases the structural diversity of available unsaturated δ-lactam-derived hydrazide–hydrazones, enabling variability in the substituent at position 6 and the introduction of an additional substituent at position 5. It also allows for the switchable, selective formation of 3,6-dihydropyridin-2(1*H*)-ones and 5,6-dihydropyridin-2(1*H*)-ones.

## 2. Results and Discussion

**Scheme 1:**
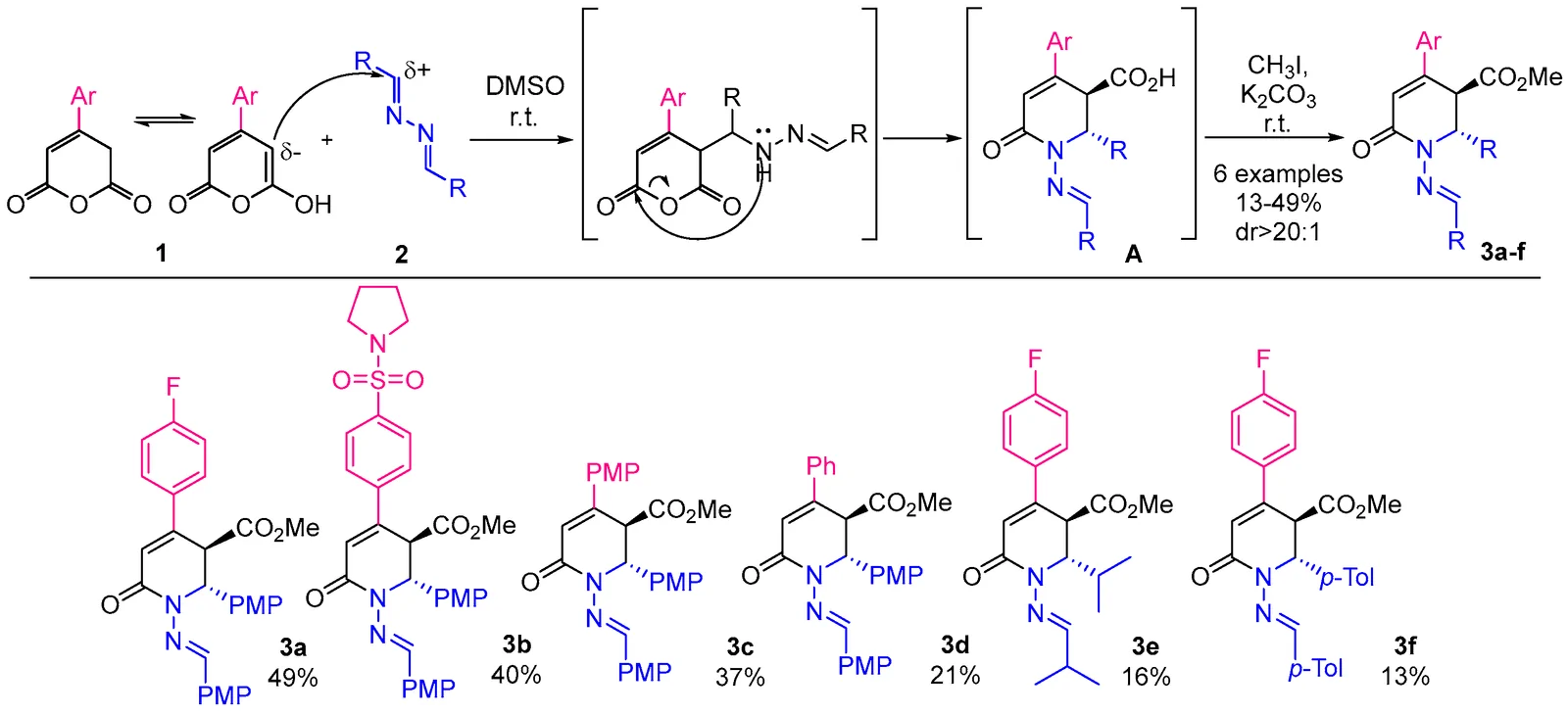
Preparation of hydrazide–hydrazones with 5,6-dihydropyridin-2(1H)-one core from 3-arylglutaconic anhydrides and aldazines. **1**: *C*-nucleophilic enolized anhydride; **2**: Imine; **A**: reaction product A; **3a–f**: Six esters **3a–f** with a 5,6-dihydropyridin-2(1H)-one core in moderate yields.

Our investigation began with conducting a reaction of anhydride **1a** (Ar = *p*-FC_6_H_4_) with aldazine **2a** (R = *p*-OMeC_6_H_4_) in DMSO at room temperature (Scheme 1). NMR monitoring revealed that at first acid A was formed via the Castagnoli–Cushman reaction [25,27] pathway. The key steps of CCR include Mannich-type addition of a *C*-nucleophilic enolized anhydride (Scheme 1, compound **1**, right structure) to the electrophilic carbon atom of the imine and subsequent intramolecular *N*-acylation. Notably, compound A was formed as a single regio- and diastereomer (according to ^1^H NMR), in contrast to the similar reactions of anhydride **1a** with *N*-alkyl imines [28] and oximes [29]. Similar acids have been shown to be thermally unstable [28], which are difficult to isolate in pure form due to decarboxylation. Therefore, in this work, we performed in situ esterification using a methyl iodide/potassium carbonate system. This protocol enabled us to isolate six esters **3a–f** with a 5,6-dihydropyridin-2(1H)-one core in moderate yields (13–49% for two steps). The relative *trans*-configuration for compound **3** was assigned based on the ^3^J_HH_ values (<1 Hz) of the vicinal methine protons from the lactam ring, according to our previous studies [28] on a similar reaction involving *N*-alkyl imines and 3-arylglutaconic anhydrides (the *cis*-isomers demonstrated ^3^J_HH_ values of >5 Hz).

**Scheme 2:**
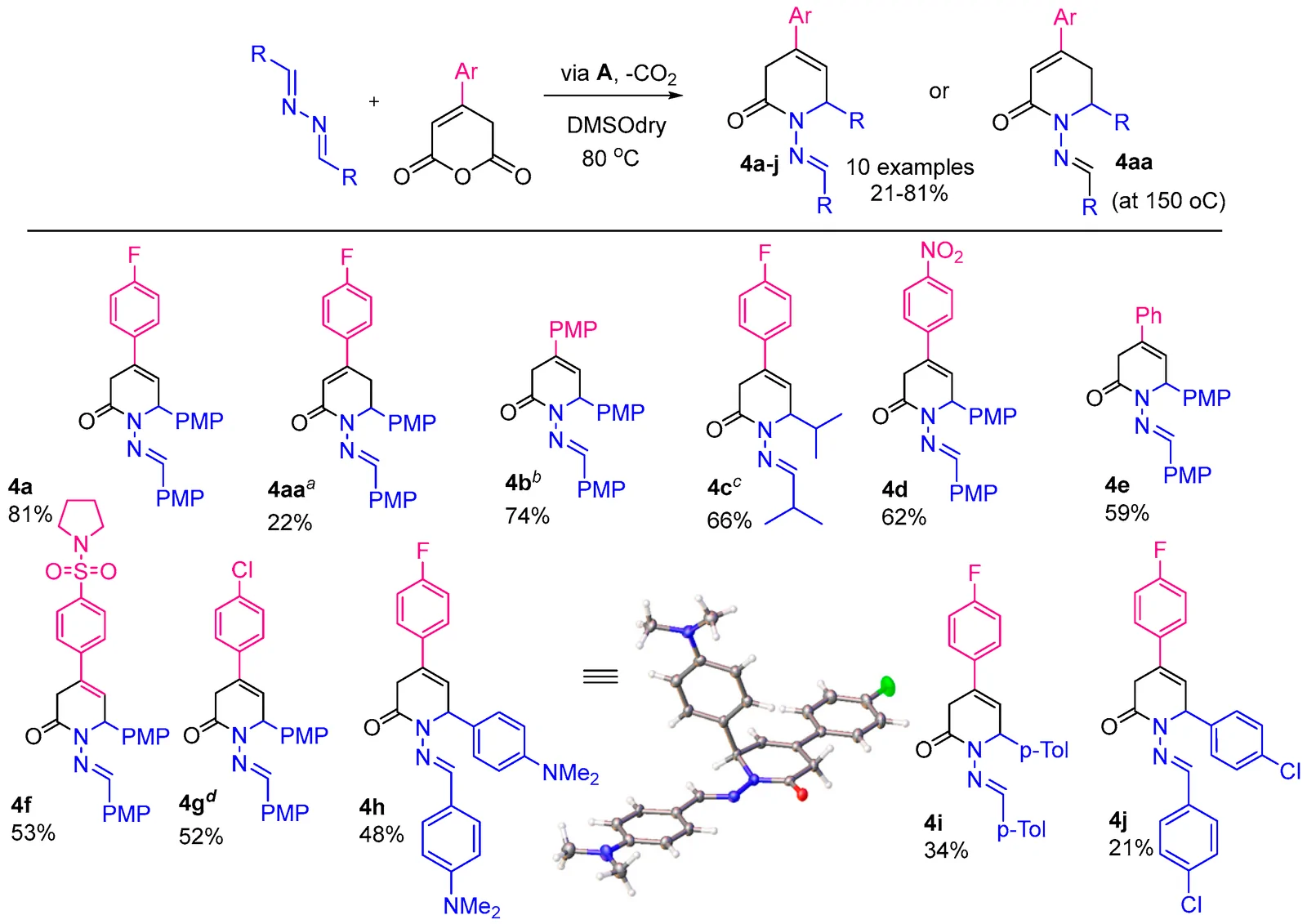
Preparation of hydrazide–hydrazones with 3,6-dihydropyridin-2(1H)-one core from 3-arylglutaconic anhydrides and aldazines. *^a^* Isolated from the reaction conducted at 150 °C in the presence of Sc(OTF)_3_ as a catalyst. *^b^* PMP = *p*-methoxyphenyl. *^c^* Conducted at 60 °C. *^d^* Reaction was conducted in the presence of Sc(OTf)_3_ (10%mol). **A**: reaction product A from Scheme 1; **4a–j**: ten novel cyclic hydrazide–hydrazone compounds; **4aa**: 5,6-dihydropyridin-2(1H)-one.

Considering that the initial reaction products **A** are prone to decarboxylation, we investigated the possibility of a targeted synthesis of the corresponding products **4** (Scheme 2). We screened the reaction conditions in order to identify the optimal parameters for the isolation of the desired product **4** (ESI, Table S1). The best results were achieved using **2** equivalents of anhydride in dry DMSO at 80 °C for 16 h without a catalyst. The protocol allowed the isolation of ten novel cyclic hydrazide–hydrazone compounds **4a–j** in up to 81% yield. It should be noted that the expected [28] double-bond migration occurred alongside anionic decarboxylation, leading to the formation of another regioisomer, namely 3,6-dihydropyridin-2(1*H*)-one. This isomerization is presumably the result of tautomerization of the allyl anion due to stabilization of the negative charge by the electron-withdrawing carbonyl group.

Both electron-rich and electron-poor aryl groups were well tolerated in position 3 of the anhydride, including the medicinally relevant sulfonamide group (compound **4f**). The scope of the imine substrate included symmetrical aldazines with aryl or alkyl groups. Substrates with strong electron-donating groups, such as alkoxy and dialkylamino groups, provided higher yields than substrates with weaker electron-donating alkyl or electron-withdrawing halogen groups. Interestingly, an aliphatic enolizable aldazine with an isopropyl moiety produced the corresponding product, **4c**, in a 66% yield. Previously, similar substrates (imines and oximes) were found to be unreactive towards 3-arylglutaconic anhydrides. The structure of 3,6-dihydropyridin-2(1*H*)-one and the *E*-configuration of compound **4h** were determined using X-ray crystallographic data (CCDC 2448075).

While screening reaction conditions for the synthesis of compound **4**, we found that conducting the reaction at a higher temperature (150 °C) in the presence of scandium triflate leads to the formation of a second isomer, **4aa** (5,6-dihydropyridin-2(1H)-one). It can be isolated in a pure form after preparative HPLC. However, our attempts to synthesize product **4aa** by heating pure, isolated compound **4a** and/or treating it with a base (DBU) only resulted in the decomposition of the starting material. Although for similar compounds with *N*-alkyl [30], NOH [29] or NH [31] functional groups the same approach was successful.

**Figure 3:**
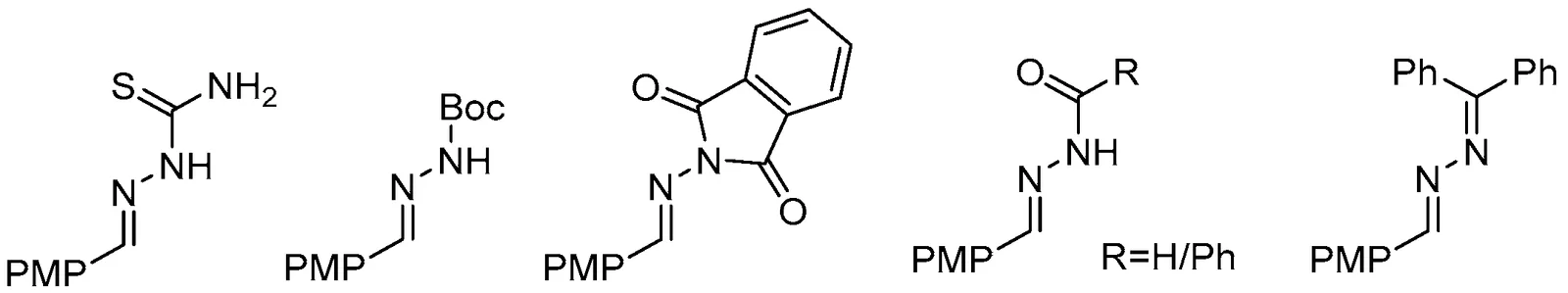
Structures of *N*-protected hydrazones, which were found to be unsuccessful reaction partners for 3-arylglutaconic anhydrides.

We also investigated switching from symmetrical aldazines to different types of structurally related, unsymmetrical substrates. Specifically, we examined *N*-substituted hydrazones with the general formula ArCH=N-N(H)PG, where PG is either a C(S)NH_2_, Boc, phthalimide, formyl, benzoyl, or benzhydrylidene moiety (Figure 3). However, no reaction occurred with 3-arylglutaconic anhydrides under various conditions. Therefore, we conclude that the presence of a second *N*-arylidene or *N*-alkylidene moiety in the substrate’s structure is crucial for the reactivity of the first C=N bond and for the developed dihydropyridone-based hydrazide–hydrazone approach. Presumably, moieties other than the *N*-arylidene/*N*-alkylidene serve as electron acceptors, reducing the nucleophilicity of the second nitrogen atom and/or creating steric hindrance. Both of these factors slow down the N-acylation step required to form the ring.

**Scheme 3:**
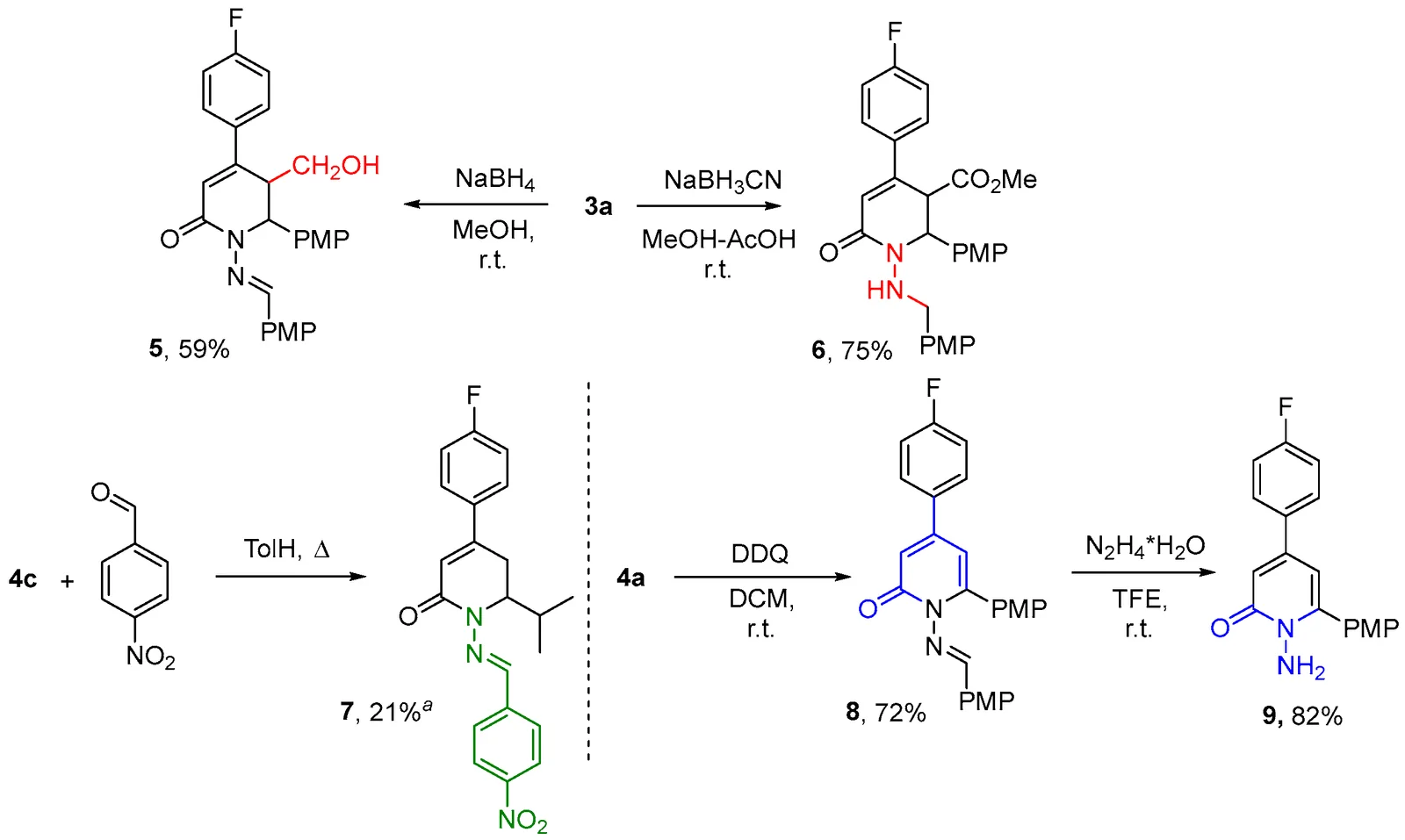
Post-condensational modifications of compounds **3** and **4**: reduction of side groups, oxidation of the core, exchange and deprotection of the hydrazide moiety. *^a^* A thermally induced isomerization to 5,6-dihydropyridin-2(1*H*)-one occurred in addition to the target reaction. **3a**: Methyl (2RS,3RS)-4-(4-Fluorophenyl)-1-(((E)-4-methoxybenzylidene)amino)-2-(4-methoxyphenyl)-6-oxo-1,2,3,6-tetrahydropyridine-3-carboxylate; **4a**: (E)-4-(4-Fluorophenyl)-1-((4-methoxybenzylidene)amino)-6-(4-methoxyphenyl)-3,6-dihydropyridin-2(1H)-one; **4c**: (E)-4-(4-Fluorophenyl)-6-isopropyl-1-((2-methylpropylidene)amino)-3,6-dihydropyridin-2(1H)-one. **5**: Alcohol; **6**: N-alkyl hydrazide; **7:** Unsymmetrical 1-N-arylidene/6-alkyl product; **8**: N-arylidene pyridine-2-one; **9**: NH2-hydrazide.

The synthetic potential of compounds **3a** and **4a** was demonstrated through a series of post-condensational modifications (see Scheme 3). 5,6-Dihydropyridin-2(1H)-one 3a was found to be selectively reduced with various hydride reagents. Reduction of the ester moiety to an alcohol **5** occurred when sodium borohydride was used. In contrast, treatment of the same substrate with sodium cyanoborohydride reduced the C=N bond only, yielding *N*-alkyl hydrazide **6**. Another important transformation involving the hydrazide–hydrazone moiety was the exchange of the *N*-alkylidene residue with an *N*-arylidene using *p*-nitrobenzaldehyde. This allowed for the preparation of unsymmetrical 1-*N*-arylidene/6-alkyl product **7** from 3,6-dihydropyridin-2(1H)-one **4c**. Notably, compound **7** could not be obtained selectively from the reaction of the anhydride and the unsymmetrical aldazine *i*PrCH=N-N=CH*p*-C_6_H_4_, as both [26] azine moieties would react to give isomeric products. Additionally, the dihydropyridine core was shown to be oxidizable with DDQ under mild conditions to produce *N*-arylidene pyridine-2-one **8**. The latter was then treated with hydrazine in trifluoroethanol, yielding deprotected NH_2_-hydrazide **9**.

## 3. Materials and Methods

### 3.1. General Information

NMR spectra were recorded with a 400 MHz Bruker Avance III spectrometer (Bruker, Billerica, MA, USA) (400.13 MHz for ^1^H and 100.61 MHz for ^13^C) in CDCl_3_ or DMSO-*d*_6_ and were referenced to residual solvent proton signals (δ_H_ = 7.26 and 2.50, respectively) and solvent carbon signals (δ_C_ = 77.16 and 39.52, respectively). Mass spectra were recorded with an HRMS-ESI-qTOF spectrometer Nexera LCMS-9030 (Shimadzu, Kyoto, Japan) or MaXis II Bruker Daltonic GmbH (Bruker, Billerica, MA, USA) (electrospray ionization mode, positive ions detection). Flash column chromatography on silica (Merck, 230–400 mesh) was carried out using the Biotage Isolera Prime instrument (Biotage, Uppsala, Sweden). TLC was performed on aluminum-backed pre-coated plates (0.25 mm) with silica gel 60 F254 with a suitable solvent system and was visualized using UV fluorescence. Preparative HPLC was carried out on a compact preparative system ECOM ECS28P00 (ECOM, Chrastany u Prahy, Czech Republic), equipped with a spectrophotometric detector or Shimadzu LC-20AP (Shimadzu, Kyoto, Japan). Column: YMC-Pack SIL-06 (YMC, Kyoto, Japan), 5 µm, 250 × 20 mm or Agilent Zorbax prepHT XDB-C18 (Agilent, Santa Clara, California), 5 μm, 21.2 × 150 mm. Dimethylsulfoxide, DMSO, was dried by distillation from calcium hydride and was stored over activated molecular sieves 4Å.

### 3.2. General Procedure for the Preparation of Lactams 3a-f and Their Analytical Data

In a screw-cap vial equipped with a magnetic stir bar, the corresponding arylglutaconic anhydride [28] 1 (2 equivalents, 0.06–0.54 mmol) and the corresponding aldazine 2 (1 equivalents, 0.03–0.27 mmol) were mixed in dry DMSO (0.06–0.54 M 0.5 mL). The resulting mixture was placed in a pre-heated to 30 °C metal heating block. DMSO was removed from the reaction mixture after 16 h using freeze-drying at 10^−2^ mbar. Next, the reaction mixture was dissolved in acetonitrile, ACN (1 mL), followed by the addition of CH_3_I (5 equivalents, 0.3–1.35 mmol) and K_2_CO_3_ (1.5 equivalents, 0.09–0.41 mmol). After 16 h, the mixture was concentrated under reduced pressure and diluted with EA (20 mL) and water (20 mL). The aqueous layer was extracted with EA (3 × 20 mL), then the combined organic phase was dried over Na_2_SO_4_ and evaporated. Then, the resulting mixture was purified by column chromatography on silica gel with a linear gradient (5−50%) of acetone in hexane (total volume of eluent, 400 mL) to provide pure compounds (**3a–f**). Prepared compound **3** should not be stored as chloroform solutions due to possible hydrolysis.

#### 3.2.1. Methyl (2RS,3RS)-4-(4-Fluorophenyl)-1-(((E)-4-methoxybenzylidene)amino)-2-(4-methoxyphenyl)-6-oxo-1,2,3,6-tetrahydropyridine-3-carboxylate (**3a**)

Yield 35 mg, 49%, dr > 20:1, yellow solid. ^1^H NMR (400 MHz, CDCl_3_) δ 8.62 (s, 1H), 7.74–7.60 (m, 2H), 7.40–7.29 (m, 2H), 7.24–7.19 (m, 2H), 7.04 (t, J = 8.62 Hz, 2H), 6.90–6.79 (m, 4H), 6.49 (s, 1H), 5.80 (d, J = 1.69 Hz, 1H), 4.07 (d, J = 1.72 Hz, 1H), 3.82 (s, 3H), 3.76 (s, 3H), 3.72 (s, 3H). ^13^C NMR (101 MHz, CDCl_3_) δ = 170.3, 163.7 (d, J = 251.0), 161.7, 161.6, 159.5, 151.1, 142.8, 133.0 (d, J = 3.4), 129.7, 129.6, 128.3 (d, J = 8.5), 127.3, 122.6 (d), 116.1 (d, J = 21.7), 114.6, 114.1, 62.9, 55.5, 55.4, 53.4, 51.4. ^19^F NMR (376 MHz, CDCl_3_) δ −110.72. HRMS (ESI) *m*/*z*: [M+H]^+^ Calcd for C_28_H_26_FN_2_O_5_^+^ 489.1820; Found 489.1821.

#### 3.2.2. Methyl (2RS,3RS)-1-(((E)-4-Methoxybenzylidene)amino)-2-(4-methoxyphenyl)-6-oxo-4-(4-(pyrrolidin-1-ylsulfonyl)phenyl)-1,2,3,6-tetrahydropyridine-3-carboxylate (**3b**)

Yield 19 mg, 40%, dr > 20:1, yellow oil. ^1^H NMR (400 MHz, CDCl_3_) δ 8.66 (s, 1H), 7.84–7.75 (m, 2H), 7.68–7.61 (m, 2H), 7.51–7.42 (m, 2H), 7.24–7.16 (m, 2H), 6.92–6.80 (m, 4H), 6.58 (s, 1H), 5.86–5.79 (m, 1H), 4.10 (d, J = 1.67 Hz, 1H), 3.82 (s, 3H), 3.77 (s, 3H), 3.74 (s, 3H), 3.27–3.18 (m, 4H), 1.82–1.72 (m, 4H). ^13^C NMR (126 MHz, CDCl_3_) δ ^13^C NMR (126 MHz, CDCl_3_) δ = 169.8, 161.7, 161.0, 159.4, 151.9, 142.3, 140.8, 138.1, 132.0, 129.7, 128.0, 127.2, 126.9, 126.8, 124.6, 114.5, 114.0, 62.8, 55.4, 55.3, 53.4, 51.2, 47.9, 25.3. HRMS (ESI) *m*/*z*: [M+H]^+^ Calcd for 604.2112 C_32_H_34_N_3_O_7_S^+^; Found 604.2112.

#### 3.2.3. Methyl (2RS,3RS)-1-(((E)-4-Methoxybenzylidene)amino)-2,4-bis(4-methoxyphenyl)-6-oxo-1,2,3,6-tetrahydropyridine-3-carboxylate (**3c**)

Yield 43 mg, 37%, dr > 20:1, yellow solid. ^1^H NMR (400 MHz, CDCl_3_) δ 8.59 (s, 1H), 7.69–7.57 (m, 2H), 7.38–7.32 (m, 2H), 7.24–7.19 (m, 2H), 6.89–6.85 (m, 4H), 6.85–6.82 (m, 2H), 6.50 (s, 1H), 5.79–5.77 (m, 1H), 4.12 (d, J = 1.63 Hz, 1H), 3.81 (s, 3H), 3.80 (s, 3H), 3.75 (s, 3H), 3.71 (s, 3H). ^13^C NMR (101 MHz, CDCl_3_) δ 170.6, 162.0, 161.5, 161.1, 159.4, 150.4, 143.2, 129.9, 129.5, 128.9, 127.8, 127.5, 127.4, 120.5, 114.5, 114.4, 114.1, 62.8, 55.5, 55.5, 55.4, 53.3, 51.1. HRMS (ESI) *m*/*z*: [M+H]^+^ Calcd for C_29_H_29_N_2_O_6_^+^ 501.2020; Found 501.2014.

#### 3.2.4. Methyl (2RS,3RS)-1-(((E)-4-Methoxybenzylidene)amino)-2-(4-methoxyphenyl)-6-oxo-4-phenyl-1,2,3,6-tetrahydropyridine-3-carboxylate (**3d**)

Yield 26 mg, 21%, dr > 20:1, yellow solid. ^1^H NMR (400 MHz, CDCl_3_) δ 8.61 (s, 1H), 7.68–7.63 (m, 2H), 7.25–7.19 (m, 2H), 6.92–6.80 (m, 5H), 6.56 (s, 1H), 5.86–5.66 (m, 1H), 4.14 (d, J = 1.65 Hz, 1H), 3.81 (s, 3H), 3.75 (s, 3H), 3.71 (s, 3H). ^13^C NMR (101 MHz, CDCl_3_) δ 170.4, 161.7, 161.6, 159.4, 150.9, 143.9, 136.8, 129.9, 129.7, 129.6, 129.0, 127.4, 126.3, 125.2, 122.6, 114.5, 114.1, 62.9, 55.5, 55.4, 53.3, 51.3. HRMS (ESI) *m*/*z*: [M+H]^+^ Calcd for C_28_H_27_N_2_O_5_^+^ 471.1914; Found 471.1919.

#### 3.2.5. Methyl (2RS,3RS)-4-(4-Fluorophenyl)-2-isopropyl-1-(((E)-2-methylpropylidene)amino)-6-oxo-1,2,3,6-tetrahydropyridine-3-carboxylate (**3e**)

Yield 28 mg, 16%, dr > 20:1, yellow solid. ^1^H NMR (400 MHz, CDCl_3_) δ 8.24 (d, J = 5.80 Hz, 1H), 7.52–7.44 (m, 2H), 7.15–7.02 (m, 2H), 6.35 (s, 1H), 4.22 (dd, J = 7.63, 1.47 Hz, 1H), 3.85 (d, J = 1.49 Hz, 1H), 3.66 (s, 3H), 2.68–2.56 (m, 1H), 2.26–2.14 (m, 1H), 1.14 (dd, J = 6.85, 2.34 Hz, 6H), 1.01 (d, J = 6.88 Hz, 3H), 0.95 (d, J = 6.76 Hz, 3H). ^13^C NMR (101 MHz, CDCl_3_) δ 171.1, 163.8, 163.7 (d, J = 243.00 Hz), 143.2, 133.0 (d, J = 3.39 Hz), 128.2 (d, J = 8.36 Hz), 122.2, 116.2 (d, J = 21.96 Hz), 66.5, 53.1, 45.0, 32.7, 31.6, 20.3, 20.1, 19.9. ^19^F NMR (376 MHz, CDCl_3_) δ −110.9. HRMS (ESI) *m*/*z*: [M+H]^+^ Calcd for C_20_H_26_FN_2_O_3_^+^ 361.1922; Found 361.1931.

#### 3.2.6. Methyl (2RS,3RS)-4-(4-Fluorophenyl)-1-(((E)-4-methylbenzylidene)amino)-6-oxo-2-(p-tolyl)-1,2,3,6-tetrahydropyridine-3-carboxylate (**3f**)

Yield 14 mg, 13%, dr > 20:1, white solid. ^1^H NMR (400 MHz, CDCl_3_) δ 8.62 (s, 1H), 7.59 (d, J = 8.12 Hz, 2H), 7.37–7.31 (m, 2H), 7.21–7.09 (m, 6H), 7.09–6.97 (m, 2H), 6.49 (s, 1H), 5.86–5.80 (m, 1H), 4.11 (d, J = 1.63 Hz, 1H), 3.72 (s, 3H), 2.35 (s, 3H), 2.30 (s, 3H). ^13^C NMR (101 MHz, CDCl_3_) δ 170.2, 163.8 (d, J = 250.99 Hz), 161.7, 150.9, 143.0, 140.8, 138.1, 134.7, 133.0 (d, J = 3.36 Hz), 131.9, 129.9, 129.4, 128.3 (d, J = 8.46 Hz), 128.0, 126.0, 122.6, 116.1 (d, J = 21.83 Hz), 63.1, 53.4, 51.4, 21.6, 21.1. ^19^F NMR (376 MHz, CDCl_3_) δ −110.7. HRMS (ESI) *m*/*z*: [M+H]^+^ Calcd for C_28_H_26_FN_2_O_3_^+^ 457.1922; Found 457.1920.

### 3.3. Synthesis of Lactams ***4a–j*** and Their Analytical Data

The corresponding arylglutaconic anhydride [28] **1** (2 equivalents, 0.06–0.54 mmol) and the aldazine **2** (1 equivalents, 0.03–0.27 mmol) were mixed in dry DMSO (0.5 mL; 0.06–0.54 M) and placed in a screw-cap vial equipped with a magnetic stir bar. The resulting mixture was heated to 80 °C using a pre-heated metal heating block and left stirring for 16 h. After cooling to room temperature, the mixture was purified by column chromatography on silica gel with a linear gradient (5−50%) of EA in Et_2_O (total volume of eluent, 400 mL) to provide pure compounds **4a–j** *.

* For **4d**, the reaction time was 2 days;

For **4g** scandium (III) trifluoromethanesulfonate (10% mol, 0.005 mmol) was added to the reaction mixture, the reaction time was 2 days;

For **4c**, the corresponding aldazine was used in excess (5 equivalents, 1.2 mmol) and the reaction temperature was 60 °C; all prepared compounds **4** should not be stored as chloroform solutions due to possible hydrolysis.

#### 3.3.1. (E)-4-(4-Fluorophenyl)-1-((4-methoxybenzylidene)amino)-6-(4-methoxyphenyl)-3,6-dihydropyridin-2(1H)-one (**4a**)

Yield 18 mg, 81%, dr > 20:1, white solid. ^1^H NMR (400 MHz, CDCl_3_) δ = 8.61 (s, 1H), 7.61 (d, J = 8.8, 2H), 7.47–7.32 (m, 2H), 7.25–7.15 (m, 2H), 7.05 (t, J = 8.7, 2H), 6.94–6.76 (m, 4H), 6.19 (dd, J = 4.5, 2.1, 1H), 5.63 (d, J = 3.9, 1H), 3.82 (s, 3H), 3.77 (s, 3H), 3.75–3.55 (m). ^13^C NMR (101 MHz, CDCl_3_) δ ^13^C NMR (101 MHz, CDCl_3_) δ = 164.1, 162.8 (d, J = 248.0), 162.0, 159.5, 158.0, 133.9 (d, J = 3.4), 131.9, 129.8, 129.7, 128.4, 127.0, 126.9, 121.7 (d, J = 1.3), 115.7 (d, J = 21.5), 114.5, 114.1, 65.1, 55.5, 55.4, 36.0. ^19^F NMR (376 MHz, CDCl_3_) δ –110.83. HRMS (ESI) *m*/*z*: [M+H]^+^ Calcd for C_26_H_24_FN_2_O_3_^+^ 431.1756; Found 431.1773.

#### 3.3.2. (E)-1-((4-Methoxybenzylidene)amino)-4,6-bis(4-methoxyphenyl)-3,6-dihydropyridin-2(1H)-one (**4b**)

Yield 15 mg, 74%, dr > 20:1, yellow oil. ^1^H NMR (400 MHz, CDCl_3_) δ 8.61 (s, 1H), 7.71–7.54 (m, 2H), 7.45–7.34 (m, 2H), 7.26–7.20 (m, 2H), 6.98–6.61 (m, 6H), 6.16 (dd, J = 4.54, 1.90 Hz, 1H), 5.62 (q, J = 3.68 Hz, 1H), 3.82 (s, 3H), 3.81 (s, 3H), 3.77 (s, 3H), 3.69 (dd, J = 3.79, 2.17 Hz, 1H), 3.62 (dd, J = 21.04, 2.96 Hz, 1H). ^13^C NMR (101 MHz, CDCl_3_) δ 164.4, 161.9, 159.8, 159.4, 157.6, 132.3, 130.2, 129.9, 129.7, 128.4, 127.1, 126.3, 120.0, 114.4, 114.1, 114.1, 65.1, 55.5, 55.4, 35.9. HRMS (ESI) *m*/*z*: [M+H]^+^ Calcd for C_27_H_27_N_2_O_4_^+^ 443.1965; Found 443.1973.

#### 3.3.3. (E)-4-(4-Fluorophenyl)-6-isopropyl-1-((2-methylpropylidene)amino)-3,6-dihydropyridin-2(1H)-one (**4c**)

Yield 48 mg, 66%, dr > 20:1, orange oil. ^1^H NMR (400 MHz, CDCl_3_) δ 8.20 (d, J = 5.57 Hz, 1H), 7.43–7.29 (m, 2H), 7.11–6.95 (m, 2H), 6.07 (dd, J = 4.51, 2.39 Hz, 1H), 4.40 (p, J = 4.03 Hz, 1H), 3.49 (ddd, J = 21.02, 4.05, 2.45 Hz, 1H), 3.36 (dd, J = 20.94, 2.84 Hz, 1H), 2.67 (pd, J = 6.88, 5.53 Hz, 1H), 2.31 (dtt, J = 10.82, 6.94, 3.91 Hz, 1H), 1.24–1.12 (m, 6H), 1.04 (d, J = 7.07 Hz, 3H), 0.81 (d, J = 6.77 Hz, 3H). ^13^C NMR (101 MHz, Chloroform-*d*) δ 169.6, 161.7 (d, J = 248.5 Hz), 159.5, 134.4 (d, J = 3.2 Hz), 128.8, 127.4 (d, J = 7.9 Hz), 126.8, 116.1 (d, J = 21.9 Hz), 66.4, 49.4, 30.8, 21.8, 19.0. HRMS (ESI) *m*/*z*: [M+H]^+^ Calcd for C_18_H_24_FN_2_O^+^ 303.1867; Found 303.1857.

#### 3.3.4. (E)-1-((4-Methoxybenzylidene)amino)-6-(4-methoxyphenyl)-4-(4-nitrophenyl)-3,6-dihydropyridin-2(1H)-one (**4d**)

Yield 12 mg, 62%, dr > 20:1, yellow solid. ^1^H NMR (400 MHz, CDCl_3_) δ 8.66 (s, 1H), 8.25–8.21 (m, 2H), 7.66–7.54 (m, 4H), 7.25–7.21 (m, 2H), 6.90–6.85 (m, 4H), 6.43 (dd, J = 4.53, 1.99 Hz, 1H), 5.68 (q, J = 3.76 Hz, 1H), 3.74 (dd, J = 3.87, 2.21 Hz, 3H), 3.65 (dd, J = 21.07, 2.98 Hz, 3H). ^13^C NMR (101 MHz, CDCl_3_) δ 163.3, 162.1, 159.6, 158.6, 147.5, 144.0, 131.2, 129.8, 129.0, 128.4, 126.8, 126.0, 125.7, 124.1, 114.6, 114.2, 65.2, 55.5, 55.4, 35.5. HRMS (ESI) *m*/*z*: [M+H]^+^ Calcd for C_26_H_24_N_3_O_5_^+^ 458.1710; Found 458.1720.

#### 3.3.5. (E)-1-((4-Methoxybenzylidene)amino)-6-(4-methoxyphenyl)-4-phenyl-3,6-dihydropyridin-2(1H)-one (**4e**)

Yield 13 mg, 59%, dr > 20:1, yellow oil. ^1^H NMR (400 MHz, CDCl_3_) δ 8.63 (s, 1H), 7.65–7.54 (m, 2H), 7.48–7.41 (m, 2H), 7.41–7.34 (m, 2H), 7.34–7.30 (m, 1H), 7.24 (d, J = 8.71 Hz, 2H), 6.93–6.82 (m, 4H), 6.26 (dd, J = 4.53, 2.01 Hz, 1H), 5.64 (q, J = 3.73 Hz, 1H), 3.82 (s, 3H), 3.77 (s, 3H), 3.73 (dd, J = 3.81, 2.19 Hz, 1H), 3.65 (dd, J = 21.20, 3.00 Hz, 1H). ^13^C NMR (101 MHz, CDCl_3_) δ 164.3, 161.9, 159.4, 157.8, 137.8, 132.0, 130.6, 129.7, 128.8, 128.4, 128.3, 127.0, 125.2, 121.8, 114.5, 114.1, 65.1, 55.5, 55.4, 35.8. HRMS (ESI) *m*/*z*: [M+H]^+^ Calcd for C_26_H_25_N_2_O_3_^+^ 413.1860; Found 413.1875.

#### 3.3.6. (E)-1-((4-Methoxybenzylidene)amino)-6-(4-methoxyphenyl)-4-(4-(pyrrolidin-1-ylsulfonyl)phenyl)-3,6-dihydropyridin-2(1H)-one (**4f**)

Yield 45 mg, 53%, dr > 20:1, white solid. ^1^H NMR (400 MHz, CDCl_3_) δ 8.65 (s, 1H), 7.83 (d, J = 8.26 Hz, 2H), 7.61 (d, J = 8.54 Hz, 2H), 7.57 (d, J = 8.14 Hz, 2H), 7.23 (d, J = 8.51 Hz, 2H), 6.87 (dd, J = 8.60, 3.83 Hz, 4H), 6.38 (dd, J = 4.70, 2.12 Hz, 1H), 5.67 (q, J = 3.78 Hz, 1H), 3.82 (s, 3H), 3.78 (s, 3H), 3.76–3.72 (m, 1H), 3.65 (dd, J = 21.01, 2.94 Hz, 1H). ^13^C NMR (101 MHz, CDCl_3_) δ 163.6, 162.1, 159.6, 158.5, 141.9, 136.7, 131.5, 129.8, 129.4, 128.4, 128.1, 126.9, 125.7, 124.6, 114.6, 114.2, 65.3, 55.5, 55.4, 48.1, 35.6, 25.4. HRMS (ESI) *m*/*z*: [M+H]^+^ Calcd for C_30_H_32_N_3_O_5_S^+^ 546.2057; Found 546.2076.

#### 3.3.7. (E)-4-(4-Chlorophenyl)-1-((4-methoxybenzylidene)amino)-6-(4-methoxyphenyl)-3,6-dihydropyridin-2(1H)-one (**4g**)

Yield 11 mg, 52%, dr > 20:1, white solid. ^1^H NMR (400 MHz, CDCl_3_) δ 8.61 (s, 1H), 7.66–7.56 (m, 2H), 7.35 (d, J = 3.72 Hz, 4H), 7.26–7.18 (m, 2H), 6.90–6.80 (m, 4H), 6.23 (dd, J = 4.54, 1.95 Hz, 1H), 5.63 (q, J = 3.76 Hz, 1H), 3.82 (s, 3H), 3.77 (s, 3H), 3.68 (dd, J = 3.86, 2.23 Hz, 1H), 3.60 (dd, J = 21.11, 2.98 Hz, 1H). ^13^C NMR (101 MHz, Toluene-*d*_8_) δ 164.0, 162.4, 160.2, 157.6, 138.1, 137.6, 137.0, 134.4, 133.5, 130.1, 130.0, 129.1, 127.0, 123.1, 114.9, 114.6, 66.9, 55.0, 55.0, 36.5. HRMS (ESI) *m*/*z*: [M+H]^+^ Calcd for C_26_H_24_ClN_2_O_3_^+^ 447.1470; Found 447.1477.

#### 3.3.8. (E)-1-((4-(Dimethylamino)benzylidene)amino)-6-(4-(dimethylamino)phenyl)-4-(4-fluorophenyl)-3,6-dihydropyridin-2(1H)-one (**4h**)

Yield 11 mg, 48%, dr > 20:1, red solid. ^1^H NMR (400 MHz, Toluene-*d*_8_) δ 9.42 (s, 1H), 7.73–7.67 (m, 2H), 7.33–7.27 (m, 2H), 7.00–6.92 (m, 2H), 6.85–6.77 (m, 2H), 6.61–6.55 (m, 2H), 6.46–6.37 (m, 2H), 5.97 (dd, J = 4.60, 2.05 Hz, 1H), 5.59 (q, J = 3.67 Hz, 1H), 3.56 (ddd, J = 20.96, 3.76, 2.21 Hz, 1H), 3.46 (dd, J = 20.91, 2.92 Hz, 1H), 2.56 (s, 6H), 2.48 (s, 6H). ^13^C NMR (101 MHz, Toluene-*d*_8_) δ 163.6, 163.2 (d, J = 246.73 Hz), 159.1, 152.5, 150.8, 135.1 (d, J = 3.32 Hz), 129.9, 128.9, 127.4 (d, J = 7.94 Hz), 124.1, 123.2, 123.2, 115.8 (d, J = 21.28 Hz), 113.4, 112.4, 66.8, 40.5, 40.0, 36.8. ^19^F NMR (376 MHz, Toluene-*d*_8_) δ −114.52. HRMS (ESI) *m*/*z*: [M+H]^+^ Calcd for C_28_H_30_FN_4_O^+^ 457.2398; Found 457.2401.

#### 3.3.9. Crystallographic Data for **4h**

Crystals of C_28_H_29_FN_4_O (*M* = 456.55) are monoclinic, space group *P*2_1_/*n*, at 100(2) K: *a* = 13.6438(3) Å, *b* = 6.3904(2) Å and *c* = 26.9347(7) Å, *α* = 90°, *β* = 100.653(3)°, *γ* = 90°, *V* = 2307.94(11) Å^3^, *Z* = 4, *d_calc_* = 1.314 g/cm^3^, *μ* = 0.698 mm^−1^, *F*(000) = 968.0. 16,247 reflections were measured, and 4256 independent reflections (*R*_int_ = 0.0468) were used in a further refinement. The final *R*_1_ was 0.0994 (*I* ≥ 2σ (*I*)), and *wR*_2_ was 0.2876 (all data). X-ray diffraction study of 4h was performed at 100(2) K on the Rigaku XtaLAB Synergy-S (Rigaku, Tokyo, Japan) diffractometer (HyPix-6000HE type detector (Rigaku, Tokyo, Japan)) using Cu Kα (λ = 1.54184 Å) radiation. The structure was solved with the ShelXT [32] structure solution program using Intrinsic Phasing and refined with the ShelXL [33] refinement package incorporated in the OLEX2 program package [34] using Least Squares minimization. Empirical absorption correction was applied in the CrysAlisPro program complex using spherical harmonics, implemented in the SCALE3 ABSPACK scaling algorithm. The hydrogen atom positions were fixed geometrically at calculated distances and allowed to ride on the parent atoms.

CCDC 2448075 contains the supplementary crystallographic data for this paper. These data can be obtained free of charge from The Cambridge Crystallographic Data Centre via http://www.ccdc.cam.ac.uk (accessed on 23 July 2025).

#### 3.3.10. (E)-4-(4-Fluorophenyl)-1-((4-methylbenzylidene)amino)-6-(p-tolyl)-3,6-dihydropyridin-2(1H)-one (**4i**)

Yield 32 mg, 34%, dr > 20:1, yellow solid. ^1^H NMR (400 MHz, CDCl_3_) δ 8.65 (s, 1H), 7.55 (d, J = 8.03 Hz, 2H), 7.43–7.33 (m, 2H), 7.23–7.09 (m, 6H), 7.08–7.02 (m, 2H), 6.20 (dd, J = 4.66, 2.15 Hz, 1H), 5.67 (q, J = 3.40 Hz, 1H), 3.73 (ddd, J = 21.49, 3.62, 2.23 Hz, 1H), 3.62 (dd, J = 21.21, 2.81 Hz, 1H), 2.35 (s, 3H), 2.31 (s, 3H). ^13^C NMR (101 MHz, CDCl_3_) δ 164.4, 162.8 (d, J = 247.84 Hz), 156.8, 141.2, 138.0, 136.8, 133.9 (d, J = 3.33 Hz), 131.6, 129.9, 129.6, 129.4, 128.1, 126.9 (d, J = 8.10 Hz), 126.8, 121.7, 115.7 (d, J = 21.59 Hz), 65.1, 36.0, 21.6, 21.2. ^19^F NMR (376 MHz, CDCl_3_) δ -113.54. HRMS (ESI) *m*/*z*: [M+H]^+^ Calcd for C_26_H_24_FN_2_O^+^ 399.1867; Found 399.1883.

#### 3.3.11. (E)-1-((4-Chlorobenzylidene)amino)-6-(4-chlorophenyl)-4-(4-fluorophenyl)-3,6-dihydropyridin-2(1H)-one (**4j**)

Yield 22 mg, 21%, dr > 20:1, yellow solid. ^1^H NMR (400 MHz, CDCl_3_). δ 8.89 (s, 1H), 7.57 (d, J = 8.39 Hz, 2H), 7.44–7.37 (m, 3H), 7.33 (d, J = 8.40 Hz, 4H), 7.07 (t, J = 8.53 Hz, 3H), 6.16 (dd, J = 4.62, 2.01 Hz, 1H), 5.67 (q, J = 3.75 Hz, 1H), 3.72 (dt, J = 21.85, 2.67 Hz, 1H), 3.63 (dd, J = 21.31, 2.98 Hz, 1H). ^13^C NMR (101 MHz, CDCl_3_) δ = 164.7, 163.0 (d, J = 248.6), 155.8, 138.5, 137.0, 134.1, 133.5 (d, J = 3.3), 132.9, 130.4, 129.4, 129.2, 129.1, 128.4, 127.0 (d, J = 8.2), 120.9, 115.9 (d, J = 21.6), 65.6, 36.1.^19^F NMR (376 MHz, CDCl_3_) δ -113.0. HRMS (ESI) *m*/*z*: [M+H]^+^ Calcd for C_24_H_18_Cl_2_FN_2_O^+^ 439.0775; Found 439.0785.

#### 3.3.12. (E)-4-(4-Fluorophenyl)-1-((4-methoxybenzylidene)amino)-6-(4-methoxyphenyl)-5,6-dihydropyridin-2(1H)-one (**4aa**)

In a screw-cap vial equipped with a magnetic stir bar, the corresponding arylglutaconic anhydride **1a** (2 equivalents, 0.5 mmol) and the corresponding aldazine **2a** (1 equivalents, 0.24 mmol) were mixed in dry DMSO (0.5 mL, 0.5 M). The reaction mixture was heated to 150 °C for 16h using a pre-heated metal heating block. After cooling to room temperature, the mixture was separated by column chromatography on silica gel with a linear gradient (5−50%) of ethyl acetate in Et_2_O (total volume of eluent, 400 mL) to afford pure compounds **4a** (28 mg, 26%, dr > 20:1) and **4aa**.

Yield 23 mg, 22%, dr > 20:1, white solid. ^1^H NMR (400 MHz, CDCl_3_) δ 8.59 (s, 1H), 7.62 (d, J = 8.8 Hz, 2H), 7.48–7.35 (m, 2H), 7.20 (d, J = 8.7 Hz, 2H), 7.05 (t, J = 8.6 Hz, 2H), 6.85 (dd, J = 10.9, 8.7 Hz, 3H), 6.40 (d, J = 2.3 Hz, 1H), 5.38 (dd, J = 6.8, 3.3 Hz, 1H), 3.82 (s, 3H), 3.76 (s, 3H), 3.48 (ddd, J = 17.1, 6.8, 2.4 Hz, 1H), 3.11 (dd, J = 17.1, 3.3 Hz, 1H). ^13^C NMR (101 MHz, CDCl_3_) δ = 164.0, 162.8 (d, J = 248.2), 162.0, 159.5, 157.9, 133.9 (d, J = 3.4), 132.0, 129.7, 129.7, 128.4, 127.0, 126.9 (d, J = 8.1), 121.7 (d, J = 1.1), 115.7 (d, J = 21.6), 114.5, 114.1, 65.1, 55.5, 55.4, 36.0. ^19^F NMR (376 MHz, CDCl_3_) δ -113.58. HRMS (ESI) *m*/*z*: [M+H]^+^ Calcd for C_26_H_24_FN_2_O_3_^+^ 431.1756; Found 431.1774.

### 3.4. Preparation of Compounds ***5–9***

#### 3.4.1. (E)-4-(4-fluorophenyl)-5-(hydroxymethyl)-1-((4-methoxybenzylidene)amino)-6-(4-methoxyphenyl)-5,6-dihydropyridin-2(1H)-one (**5**)

Compound **3a** (45 mg, 0.09 mmol) was dissolved in MeOH (2 mL) and cooled with an ice-water bath under stirring, followed by the addition of solid NaBH_4_ (18 mg, 0.48 mmol) in one portion. After stirring for 16 h at room temperature, **5** drops of acetic acid were added to the reaction mixture. The resulting solution was diluted with ether (25 mL) and water (10 mL). The organic layer was removed, and the aqueous layer was extracted twice with 10 mL of ether. The combined organics were washed with NaHCO_3_ sat., water, and brine, dried over sodium sulfate, filtered, and concentrated. The residue was purified using HPLC on silica (mobile phase: linear gradient of acetone in hexane 2–50%, total volume 250 mL). Yield 24 mg, 59%, dr > 20:1, yellow powder.

^1^H NMR (400 MHz, CDCl_3_) δ 8.22 (s, 1H), 7.63 (d, J = 8.8 Hz, 1H), 7.41–7.27 (m, 2H), 7.18 (d, J = 8.7 Hz, 1H), 7.01 (t, J = 8.6 Hz, 2H), 6.83 (dd, J = 8.6, 5.2 Hz, 4H), 6.38 (s, 1H), 5.68 (s, 1H), 3.80 (s, 3H), 3.77–3.71 (m, 5H), 3.42 (t, J = 6.8 Hz, 1H). ^13^C NMR (101 MHz, CDCl_3_) δ 161.9, 160.5 (d, J = 242.0 Hz), 148.4, 146.9, 132.3 (d, J = 3.3 Hz), 132.1, 130.4, 129.8, 128.3 (d, J = 8.4 Hz), 127.3, 127.1, 120.9, 116.1 (d, J = 21.8 Hz), 114.6, 114.2, 62.6, 59.5, 55.5, 55.4, 49.6. HRMS (ESI) *m*/*z*: [M+Na]^+^ Calcd for C_26_H_26_FN_2_NaO_4_^+^ 483.1691; Found 483.1697.

#### 3.4.2. Methyl 4-(4-fluorophenyl)-1-((4-methoxybenzyl)amino)-2-(4-methoxyphenyl)-6-oxo-1,2,3,6-tetrahydropyridine-3-carboxylate (**6**)

Compound **3a** (20 mg, 0.04 mmol) was dissolved in a mixture of methanol and acetic acid (2 + 0.2 mL) and cooled to 0 °C with an ice-water bath, followed by the addition of solid NaBH_3_CN (8 mg, 0.13 mmol) in one portion. After stirring for 16 h at room temperature, the reaction mixture was diluted with water (15 mL) and ether (25 mL). The organic layer was separated, washed with water, and aq. NaHCO_3_ sat., water and brine, dried over Na_2_SO_4_, filtered and concentrated in vacuo to provide pure title compound as a yellow solid. Yield 15 mg, 75%, dr > 20:1.

^1^H NMR (400 MHz, CDCl_3_) δ 7.34–7.27 (m, 4H), 7.15–7.08 (m, 2H), 7.05–6.97 (m, 2H), 6.84 (dd, J = 8.6, 5.7 Hz, 4H), 6.43 (s, 1H), 5.34 (d, J = 1.5 Hz, 1H), 3.97 (s, 2H), 3.89 (d, J = 1.5 Hz, 1H), 3.79 (s, 3H), 3.76 (s, 3H), 3.65 (s, 3H). ^13^C NMR (101 MHz, CDCl_3_) δ 170.3, 163.7, 163.6 (d, J = 250.7 Hz), 159.5, 159.2, 143.5, 133.3 (d, J = 3.5 Hz), 130.8, 130.4, 129.4, 128.2 (d, J = 8.5 Hz), 127.5, 121.1, 116.0 (d, J = 21.8 Hz), 114.4, 113.9, 63.5, 55.4, 55.4, 54.2, 53.1, 51.4. HRMS (ESI) *m*/*z*: [M+H]^+^ Calcd for C_28_H_28_FN_2_O_5_^+^ 491.1977; Found 491.1979.

#### 3.4.3. (E)-4-(4-Fluorophenyl)-6-isopropyl-1-((4-nitrobenzylidene)amino)-5,6-dihydropyridin-2(1H)-one (**7**)

In a screw-cap vial equipped with a septum cap and a magnetic stir bar, compound **4c** (40 mg, 0.13 mmol) and *p*-nitrobenzaldehyde (98 mg, 0.65 mmol) were mixed in dry toluene (0.5 mL). The resulting mixture was placed in a pre-heated to 110 °C metal heating block, and a needle was inserted into the septum. After 16 h, the mixture was cooled to room temperature, concentrated under reduced pressure, and purified by column chromatography on silica gel with a linear gradient (5−50%) of acetone in hexane (total volume of eluent, 400 mL) to provide the pure title compound. Yield 11 mg, 21%, dr > 20:1, yellow solid.

^1^H NMR (400 MHz, CDCl_3_) δ 9.64 (s, 1H), 8.28–8.21 (m, 2H), 7.89–7.82 (m, 2H), 7.59–7.48 (m, 2H), 7.18–7.10 (m, 2H), 6.26 (d, J = 2.42 Hz, 1H), 4.09 (td, J = 6.89, 2.81 Hz, 1H), 3.15 (ddd, J = 17.65, 6.87, 2.49 Hz, 1H), 2.94 (dd, J = 17.61, 2.84 Hz, 1H), 2.36 (h, J = 6.82 Hz, 1H), δ 1.0 (d, J = 6.9 Hz, 3H), 1.0 (d, J = 6.7 Hz, 3H). ^13^C NMR (101 MHz, CDCl_3_) δ 164.0 (d, J = 251.18 Hz), 163.4, 148.7, 148.5, 147.9, 142.1, 133.4 (d, J = 3.37 Hz), 128.0 (d, J = 8.43 Hz), 127.9, 124.1, 116.2 (d, J = 21.81 Hz), 66.3, 31.2, 20.1, 19.5. ^19^F NMR (376 MHz, DMSO-*d*_6_) δ −110.3. HRMS (ESI) *m*/*z*: [M+H]^+^ Calcd for C_19_H_19_FN_3_O_4_^+^ 382.1561; Found 382.1571.

#### 3.4.4. (E)-4-(4-Fluorophenyl)-1-((4-methoxybenzylidene)amino)-6-(4-methoxyphenyl)pyridin-2(1H)-one (**8**)

To a stirred solution of compound **4a** (30 mg, 0.07 mmol) in dry DCM (1 mL), 4,5-dichloro-3,6-dioxocyclohexa-1,4-diene-1,2-dicarbonitrile (DDQ) (1.1 equivalents, 0.08 mmol) was added in one portion. After stirring 16 h at room temperature, the precipitate formed was centrifuged, washed with DCM (5 mL), 10% NaOH solution (5 mL), and water (5 mL), then dried in a vacuum to obtain the target product.

Yield 22 mg, 72%, dr > 20:1, white solid. ^1^H NMR (400 MHz, DMSO-*d*_6_) δ 8.76 (s, 1H), 7.61–7.55 (m, 4H), 7.38–7.32 (m, 2H), 7.10 (t, J = 8.62 Hz, 2H), 6.89–6.77 (m, 4H), 6.69 (d, J = 2.21 Hz, 1H), 6.38 (d, J = 2.29 Hz, 1H), 3.78 (s, 3H), 3.75 (s, 3H). ^13^C NMR (101 MHz, CDCl_3_) δ = 166.7, 161.6 (d, J = 283.4), 160.1, 148.9, 148.3, 133.9 (d, J = 3.3), 131.2, 130.8, 129.7, 128.8 (d, J = 8.5), 127.4, 125.9, 125.7, 116.1 (d, J = 21.7), 115.5, 114.3, 113.5, 106.5, 55.6, 55.5. ^19^F NMR (376 MHz, CDCl_3_) δ −111.93. HRMS (ESI) *m*/*z*: [M+H]^+^ Calcd for 429.1609 C_26_H_22_FN_2_O_3_^+^; Found 429.1600.

#### 3.4.5. 1-Amino-4-(4-fluorophenyl)-6-(4-methoxyphenyl)pyridin-2(1H)-one (**9**)

To the mixture of compound **7** (30 mg, 0.07 mmol) in dry trifluoroethanol (0.3 mL), hydrazine hydrate (0.2 mL) was added. After stirring at room temperature for 16 h, the precipitate formed was centrifuged, washed with Et_2_O (5 mL), and then dried in a vacuum to obtain the title product.

Yield 18 mg, 82%, dr >20:1, white solid. ^1^H NMR (400 MHz, DMSO-*d*_6_) δ 7.91–7.71 (m, 2H), 7.65 (d, J = 8.70 Hz, 2H), 7.30 (t, J = 8.83 Hz, 2H), 7.03 (d, J = 8.84 Hz, 2H), 6.76 (d, J = 2.25 Hz, 1H), 6.50 (d, J = 2.32 Hz, 1H), 3.82 (s, 3H). ^13^C NMR (101 MHz, DMSO-*d*_6_) δ 162.8 (d, J = 246.80 Hz), 160.3, 159.8, 148.2, 147.3, 133.5 (d, J = 3.09 Hz), 130.8, 129.1 (d, J = 8.50 Hz), 126.5, 115.8 (d, J = 21.52 Hz), 113.3, 111.3, 105.1, 55.3. ^19^F NMR (376 MHz, DMSO-*d*_6_) δ -112.6. HRMS (ESI) *m*/*z*: [M+H]^+^ Calcd for 311.1190 C_18_H_16_FN_2_O_2_^+^; Found 311.1191.

## 4. Conclusions

In conclusion, we have developed a novel, general approach to synthesizing δ-lactam-based hydrazide–hydrazone compounds from 3-arylglutaconic anhydrides and aldazines. Two simple, catalyst-free protocols have been developed to selectively prepare products with 3,6-dihydropyridin-2(1*H*)-one or 5,6-dihydropyridin-2(1*H*)-one cores, depending on the reaction temperature. Furthermore, all compounds were isolated as single diastereomers with respect to the hydrazone moiety and the relative configuration of the substituents at the 5,6-dihydropyridin-2(1*H*)-one core. We carried out a series of post-condensation modifications to demonstrate useful synthetic transformations of the side functional groups and the core. These transformations include the selective reduction of the ester or hydrazone moiety, hydrazone moiety exchange, the oxidation of the lactam core to 2-pyridone, and the deprotection of the hydrazone moiety to the NH_2_-hydrazide. Thus, the developed strategy enables the preparation of several medicinally relevant and poorly available *N*-functionalized δ-lactam scaffolds from the same set of substrates and a single annulation strategy. Our future studies will continue to evaluate the antibacterial and antitubercular activity profiles of the obtained compounds to identify their medical potential.

## Data Availability

The original contributions presented in this study are included in the article/Supplementary Materials. Further inquiries can be directed to the corresponding author.

## Supplementary Materials

The following supporting information can be downloaded at: https://www.mdpi.com/article/10.3390/ijms26188834/s1.
