# Supplementary material for: Catalyst-Free Assembly of δ-Lactam-Based Hydrazide–Hydrazone Compounds from 3-Arylglutaconic Anhydrides and Aldazines
Source: Int J Mol Sci. 2025 Sep 10;26(18):8834. doi: 10.3390/ijms26188834 (PMC12469989; doi:10.3390/ijms26188834)
Supplement: Supplementary file 1 [file ijms-26-08834-s001.zip › ijms-3806528-supplementary.pdf]

ELECTRONIC SUPPORTING INFORMATION FOR

Catalyst-Free Assembly of δ-Lactam-Based Hydrazide–hydrazone Compounds from 3-Arylglutaconic Anhydrides and Aldazines

Anna Ananeva[a], Elizaveta Karchuganova [a], Dar'ya Spiridonova[a], Grigory Kantin[a] and Olga Bakulina*[a]

[a] *Institute of Chemistry, Saint Petersburg State University, Saint Petersburg 199034 Russia*
* Address correspondence to this author; phone: +7 981 787 1252, fax: +7 812 428 6939; E-mail: o.bakulina@spbu.ru

Table of contents

Table S1. Optimization of reaction conditions for preparation of compound 3a 2
Copies of ^{1}H, ^{13}C and ^{19}F NMR spectra of prepared compounds 3

Table S1. Optimization of reaction conditions for preparation of compound 3a

	Reagent	Solvent	T	Loading, equiv.			Yield, %*
				Aldazine **2a**	Anhydride **1a**	Reagent	
	-	DMSO	50	1	2	-	37
1	-	DMSO	80	1	1	-	42
2		DMSO	80	1	2		54
3	-	DMSO	110	1	1	-	39
4	-	DMSO	130	1	1	-	19
5	-	Neat, no solvent	80	1	1		9
6	-	DCE	80	1	2	-	28
7	-	ACN	80	1	2	-	23
8	-	TolH	80	1	2	-	12
9	-	PhH	80	1	2	-	34
11	-	1,4-dioxane	80	1	2	-	9
12	-	Trifluoroethanol	80	1	2	-	28
13	-	DMF	80	1	2	-	40
14	$Sc(OTf)_3$	DMSO	80	1	1	0.1	44
15	BF_3*Et_2O	DMSO	80	1	1	0.1	36
16	DIPEA	DMSO	80	1	1	0.1	32
17	$Yb(OTf)_3$	DMSO	80	1	1	0.1	37
18	$In(OTf)_3$	DMSO	80	1	1	0.1	37
19	$Zn(OTf)_2$	DMSO	80	1	1	0.1	41
20	$Cu(OTf)_2$	DMSO	80	1	1	0.1	19
21	3,4-di$C_6H_5B(OH)_2$	DMSO	80	1	1	0.1	39

* NMR yields were calculated using internal standard (dibromomethane)

Copies of ^{1}H, ^{13}C and ^{19}F NMR spectra of prepared compounds

^{1}H, ^{13}C NMR and ^{19}F spectra of compound **3a**

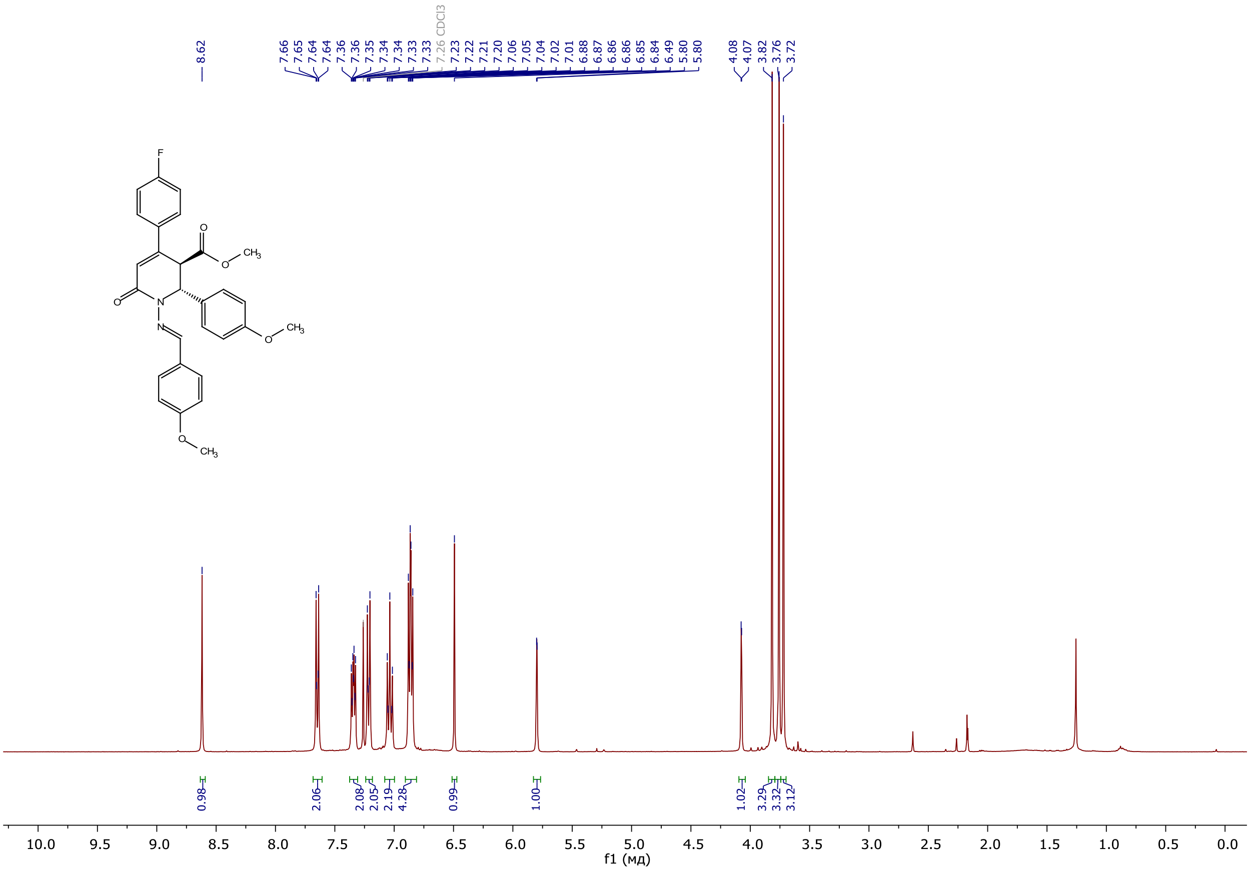

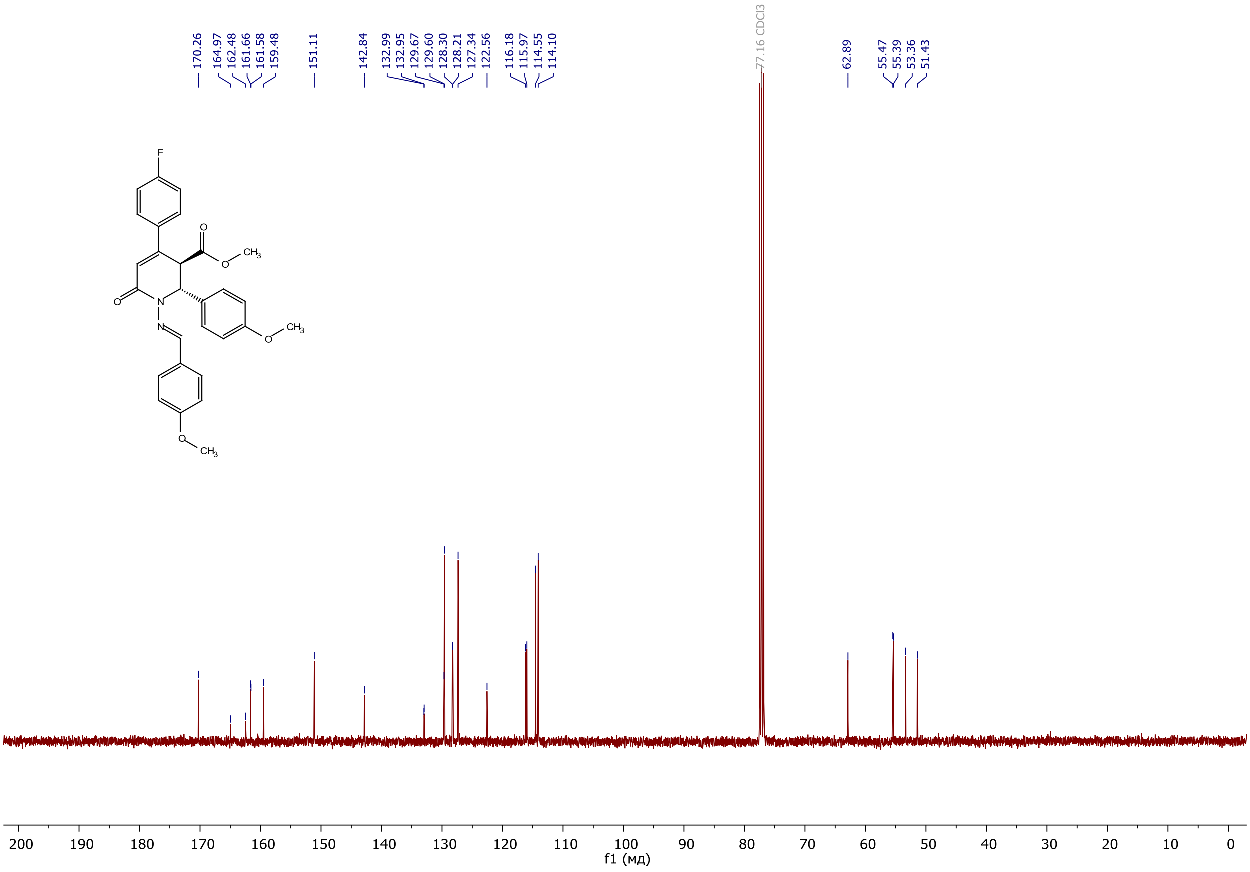

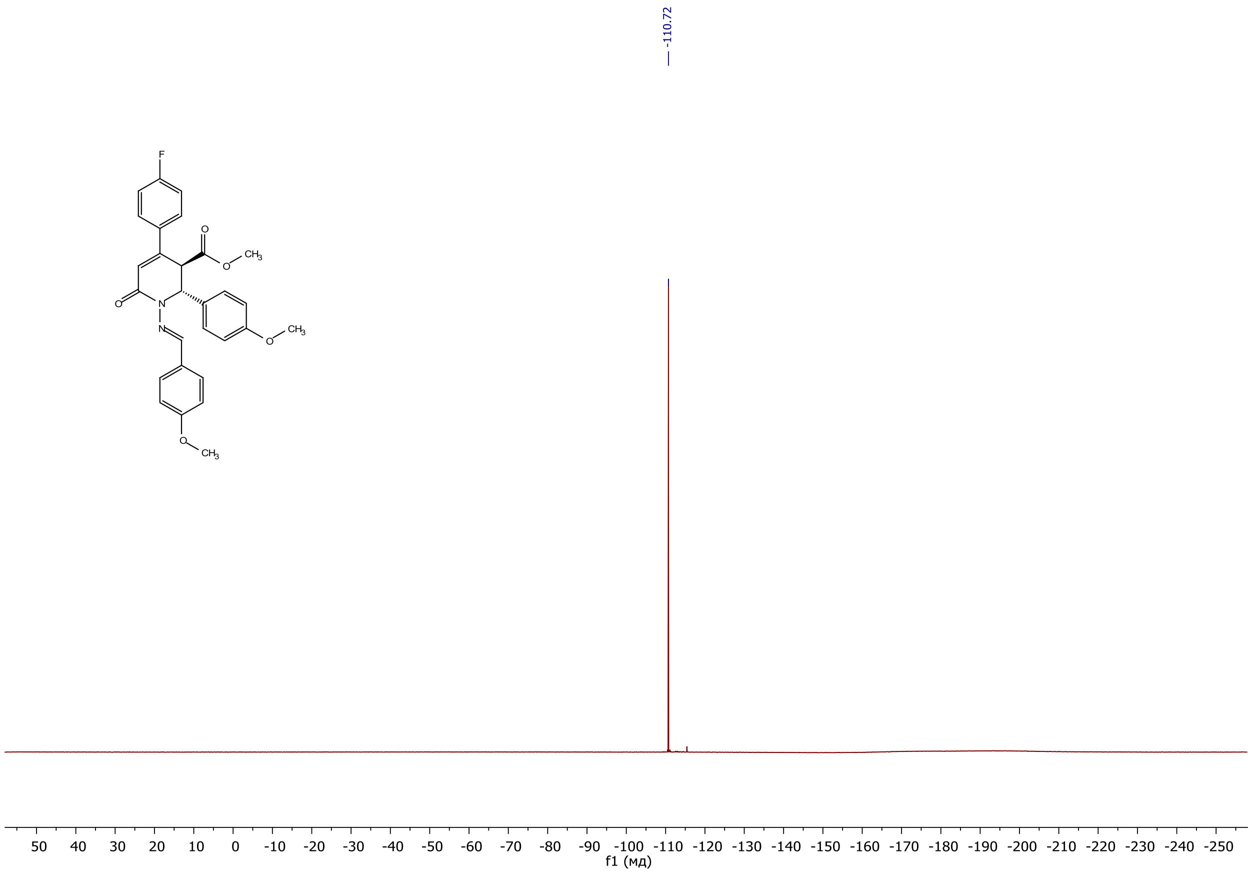

-110.72
F
O
CH3
O
N
N
O
CH3
O
CH3
50 40 30 20 10 0 -10 -20 -30 -40 -50 -60 -70 -80 -90 -100 -110 -120 -130 -140 -150 -160 -170 -180 -190 -200 -210 -220 -230 -240 -250
f1 (мд)

^1H and ^{13}C NMR spectra of compound **3b**

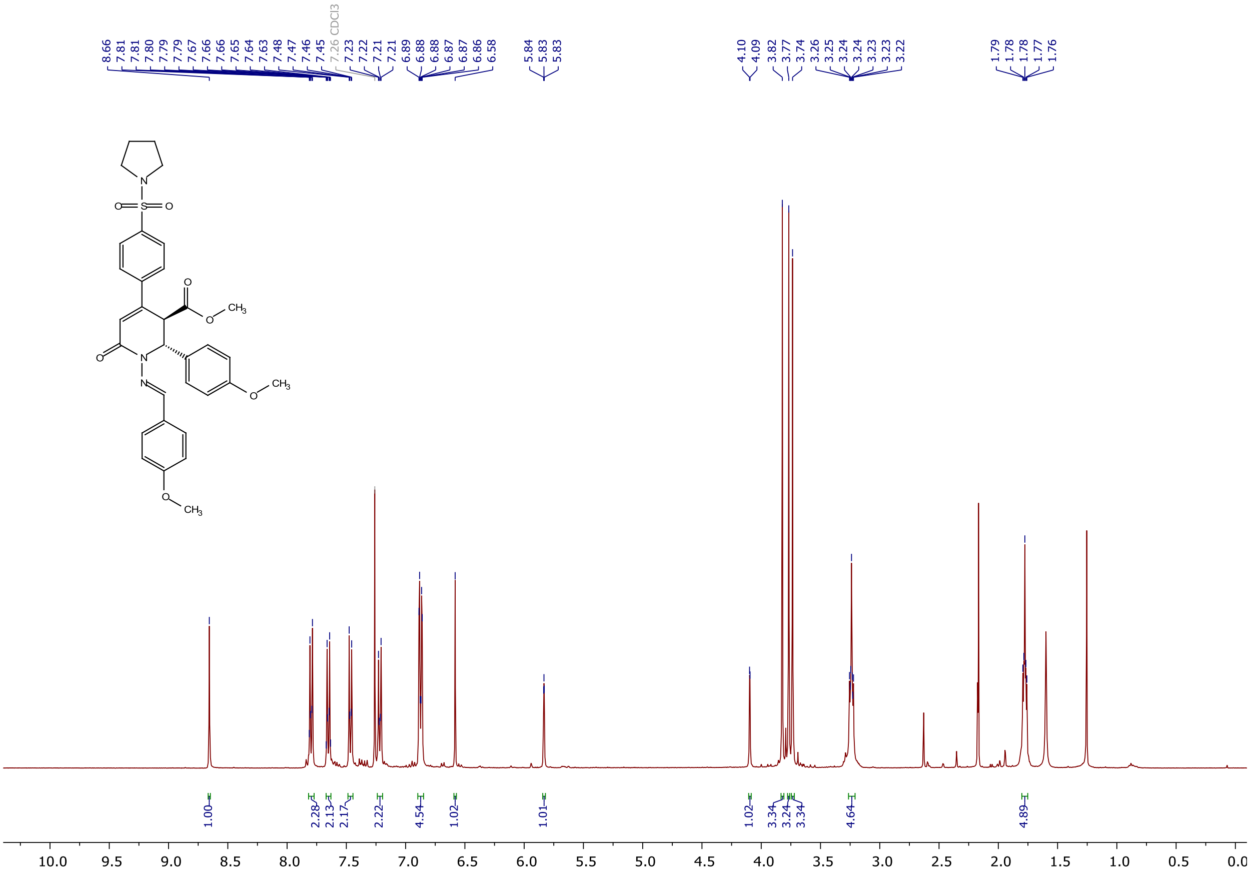

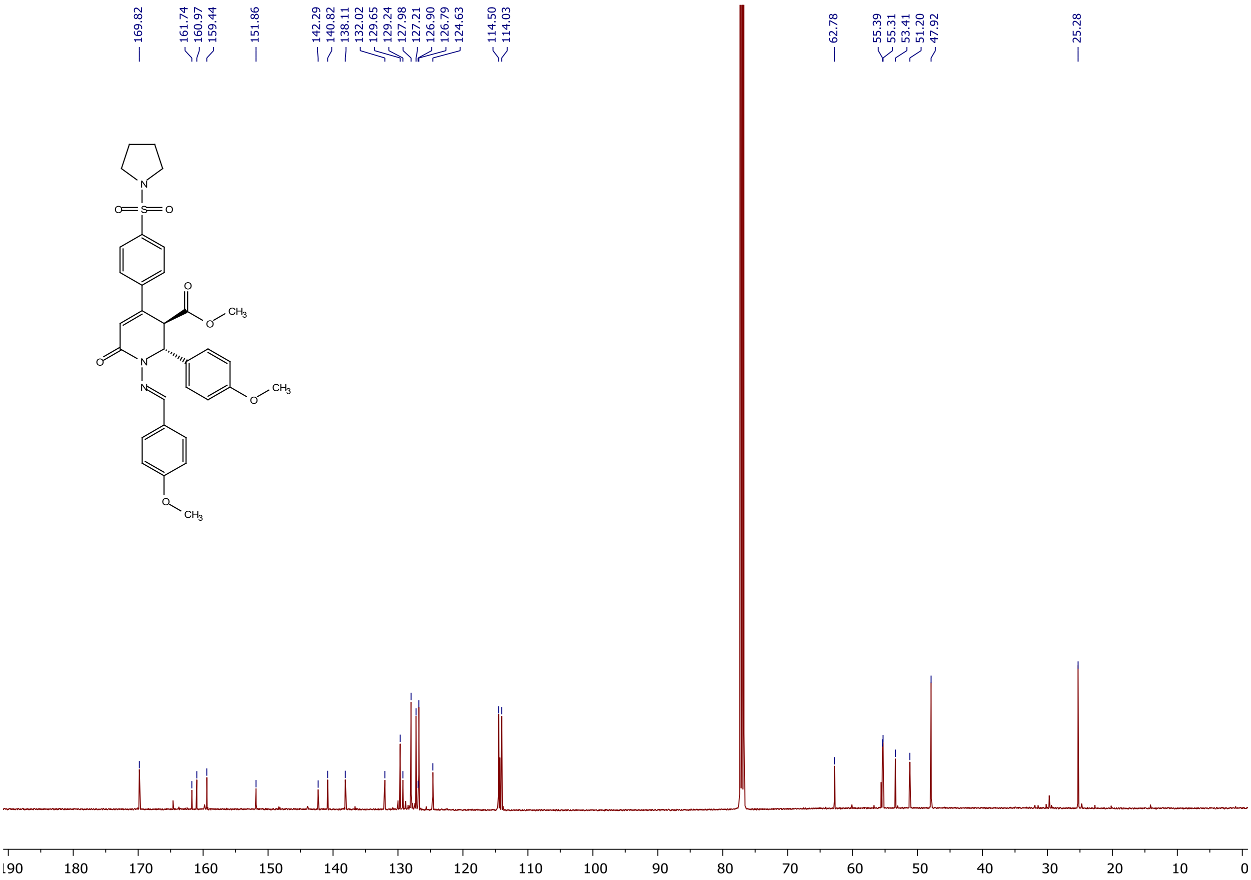

^{1}H and ^{13}C NMR spectra of compound **3c**

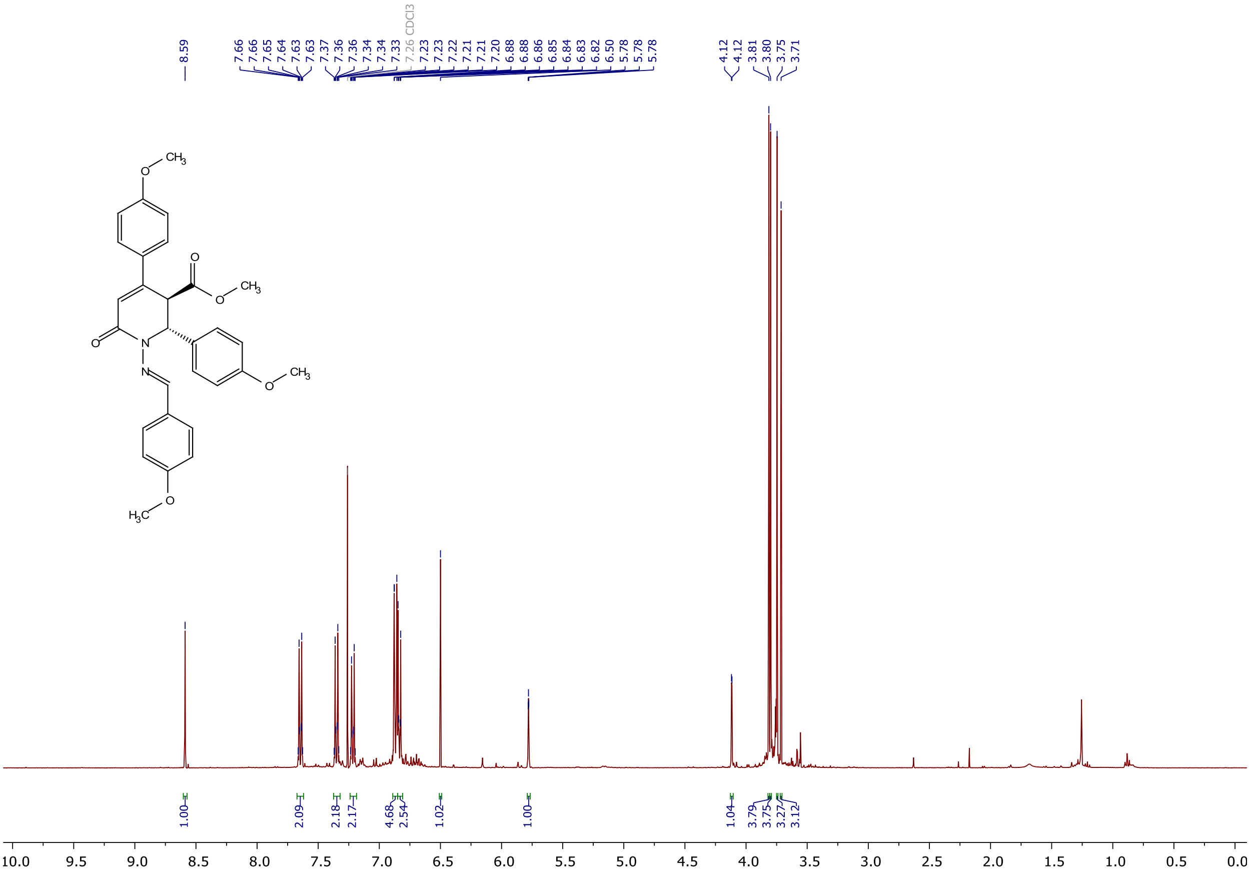

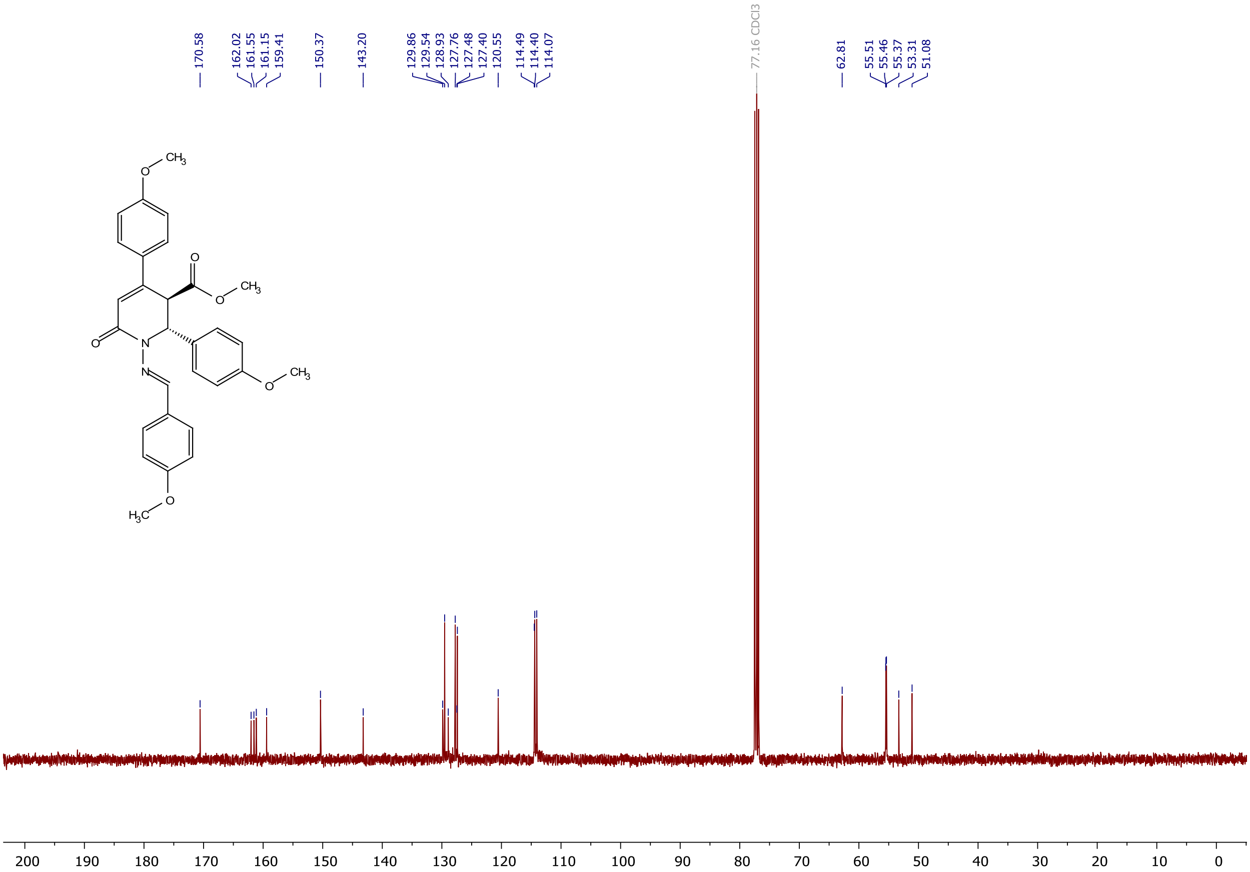

^{1}H and ^{13}C NMR spectra of compound **3d**

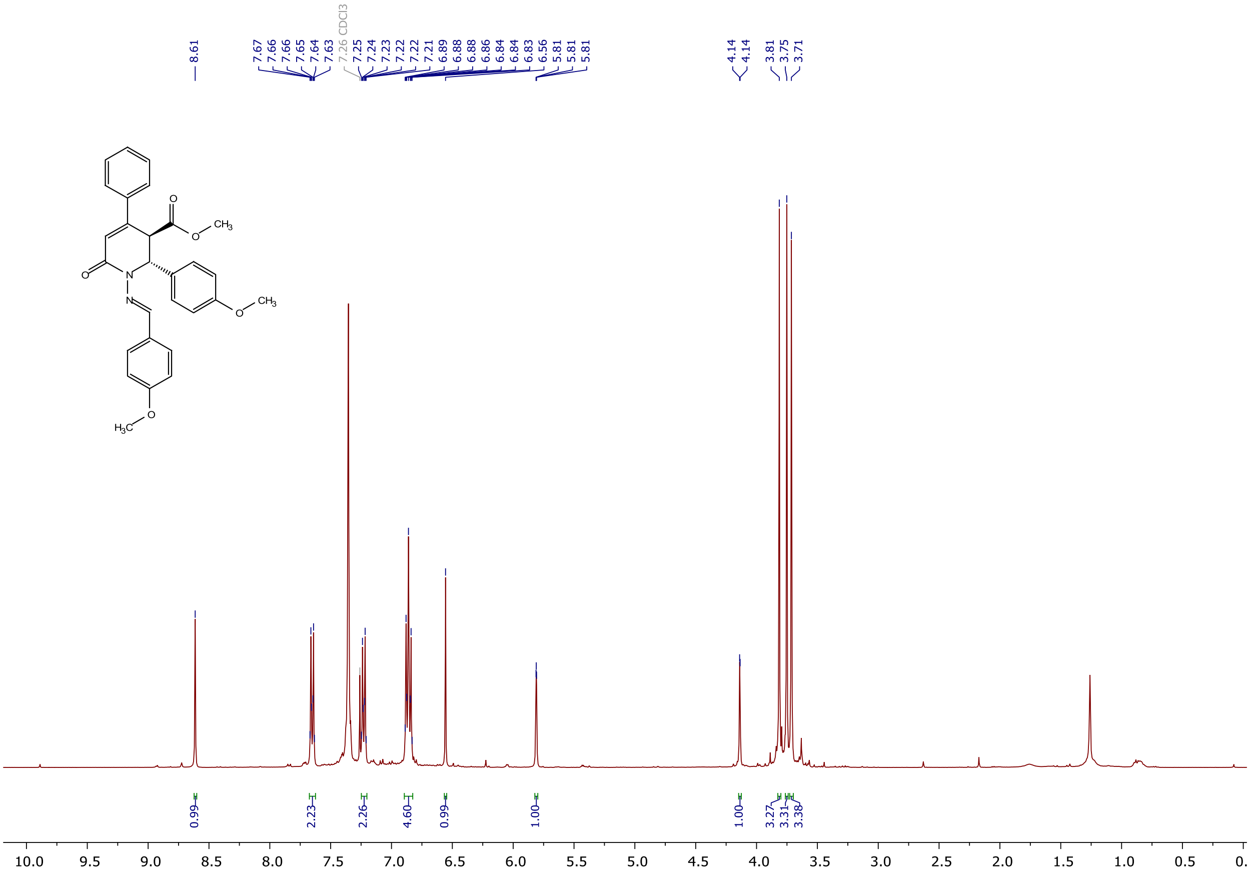

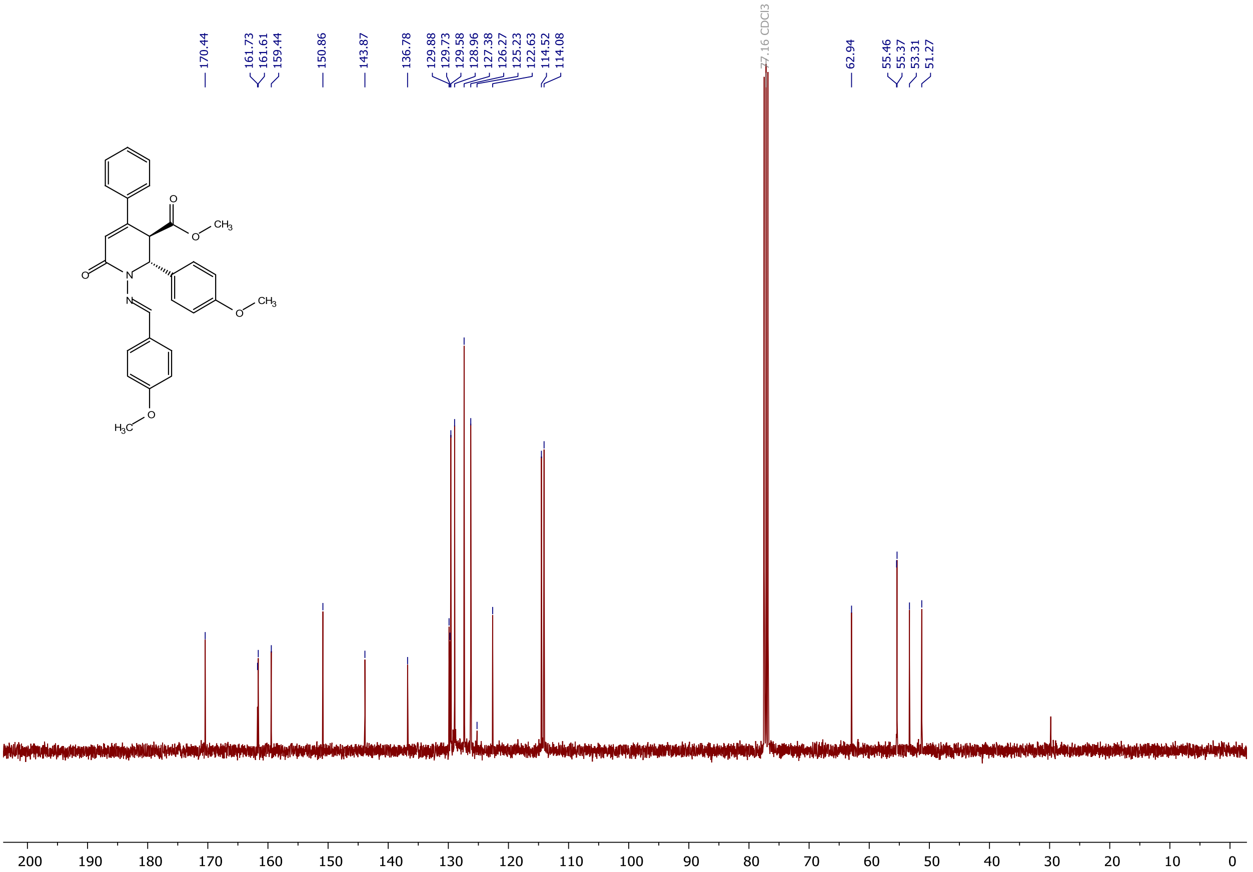

^1H, ^{13}C NMR and ^{19}F spectra of compound **3e**

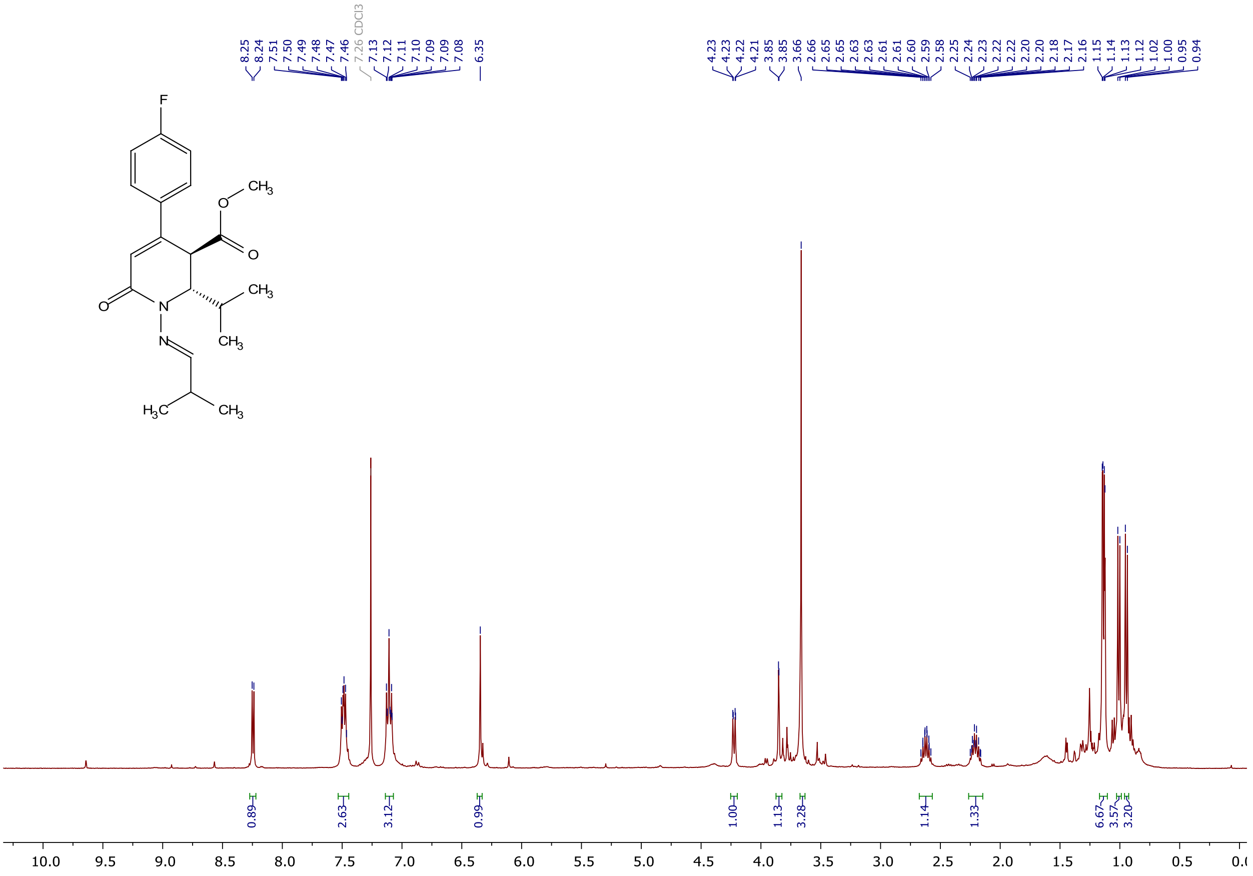

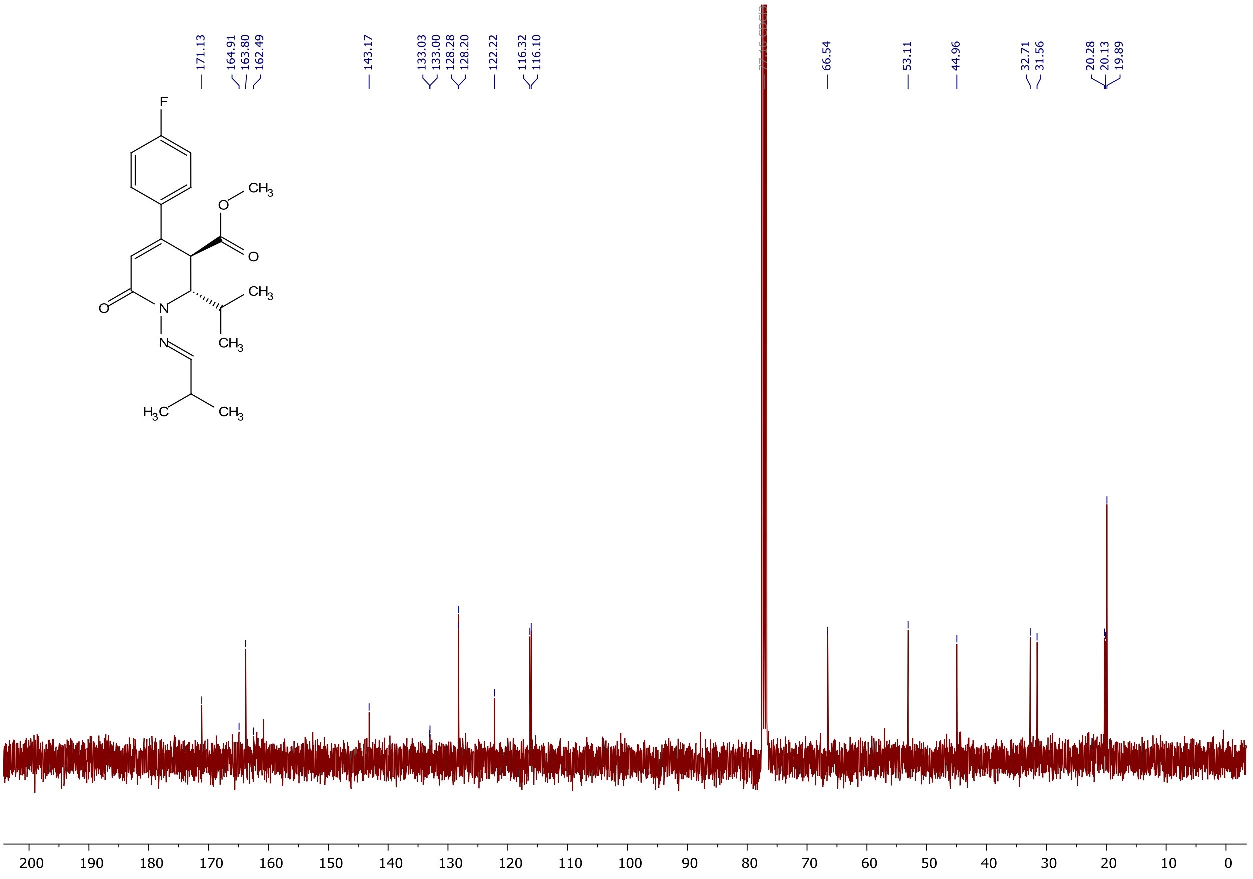

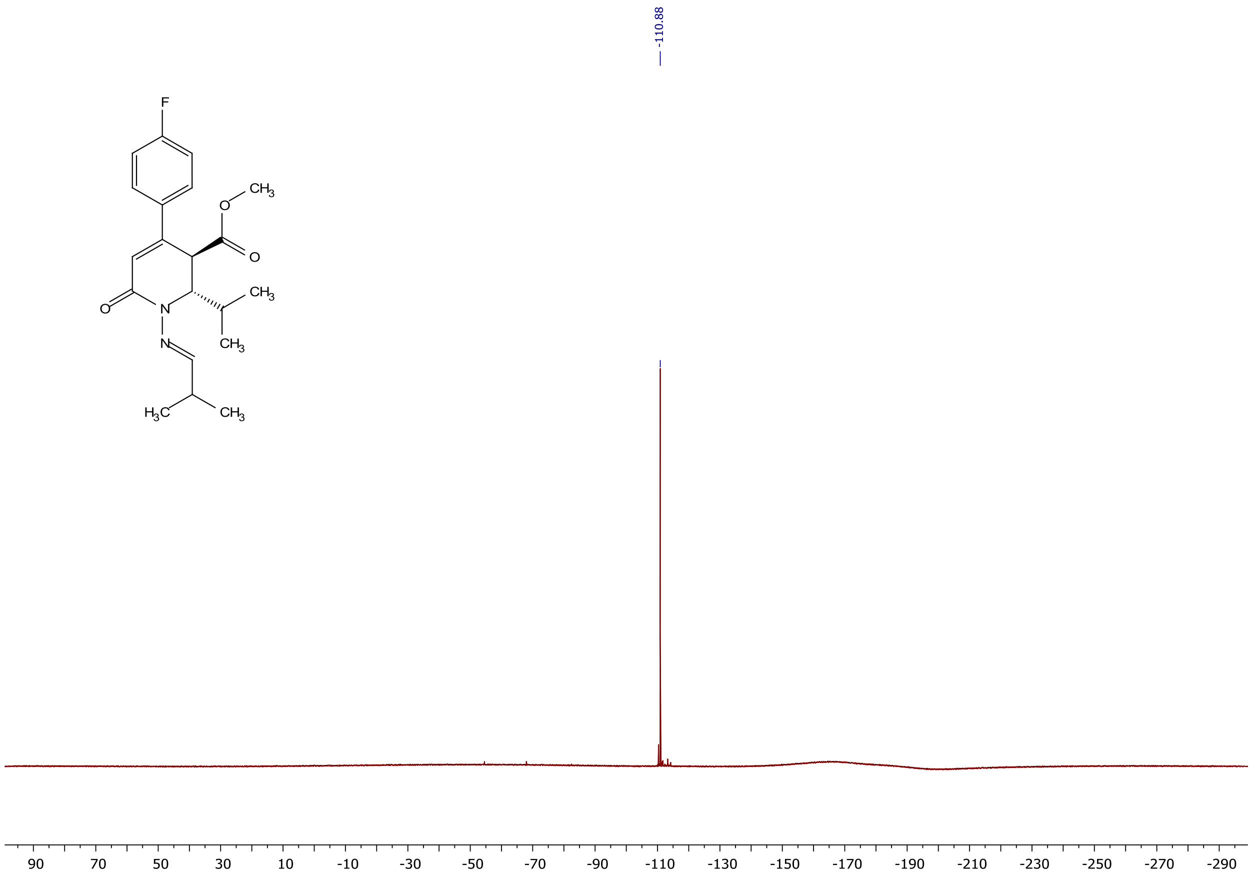

F
O
CH3
O
O
N
CH3
CH3
N
H3C
CH3
-110.88
90
70
50
30
10
-10
-30
-50
-70
-90
-110
-130
-150
-170
-190
-210
-230
-250
-270
-290

^{1}H, ^{13}C NMR and ^{19}F spectra of compound **3f**

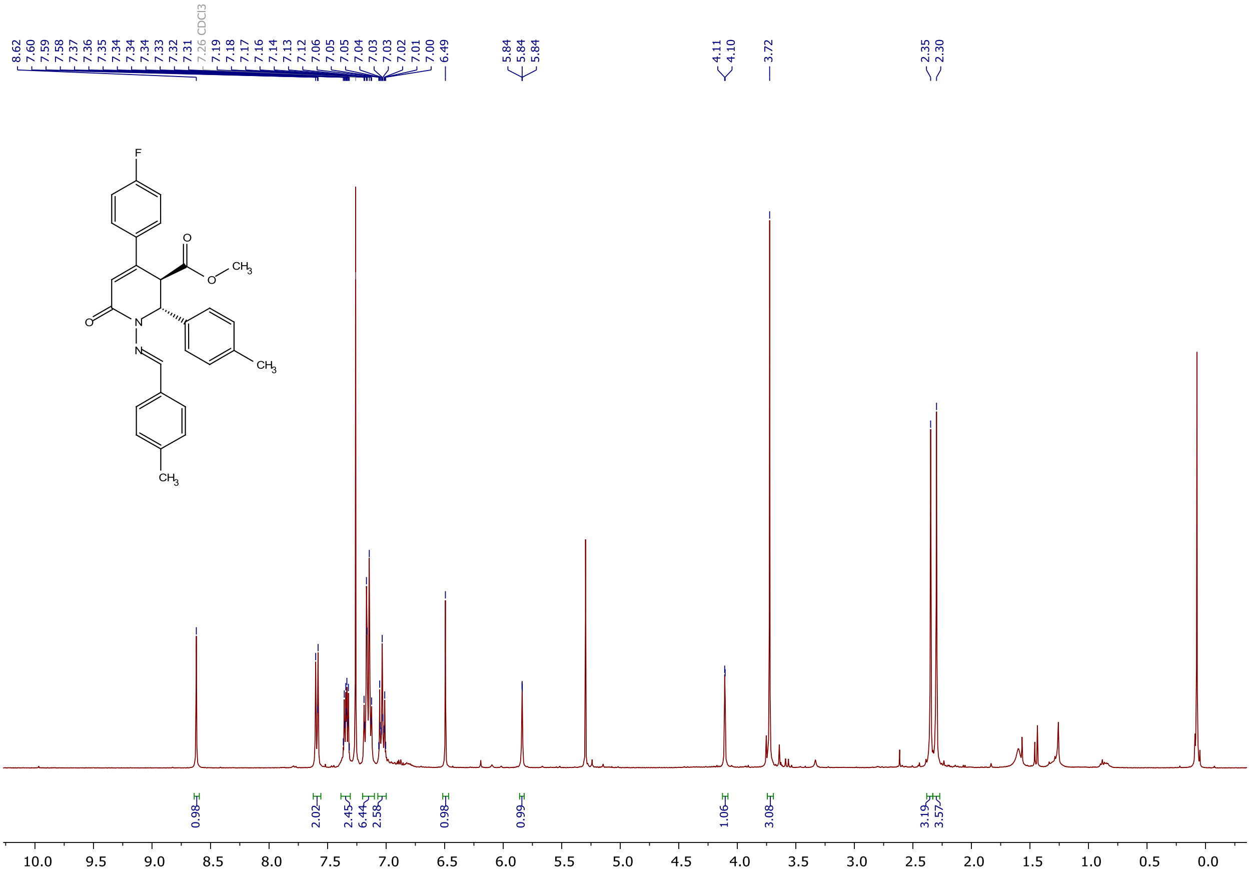

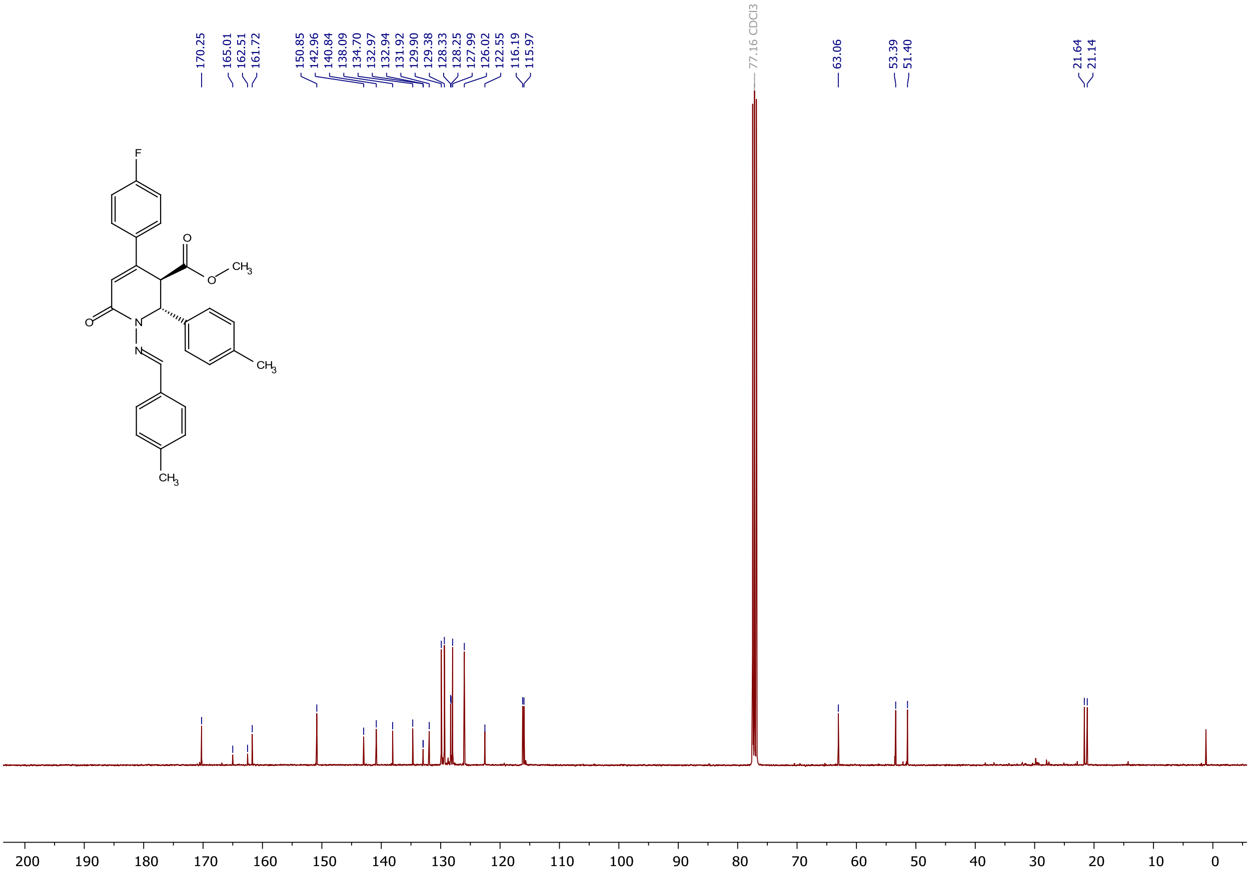

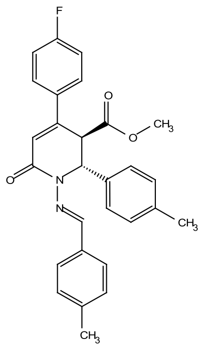
F
O
O
CH3
O
N
N
CH3
CH3

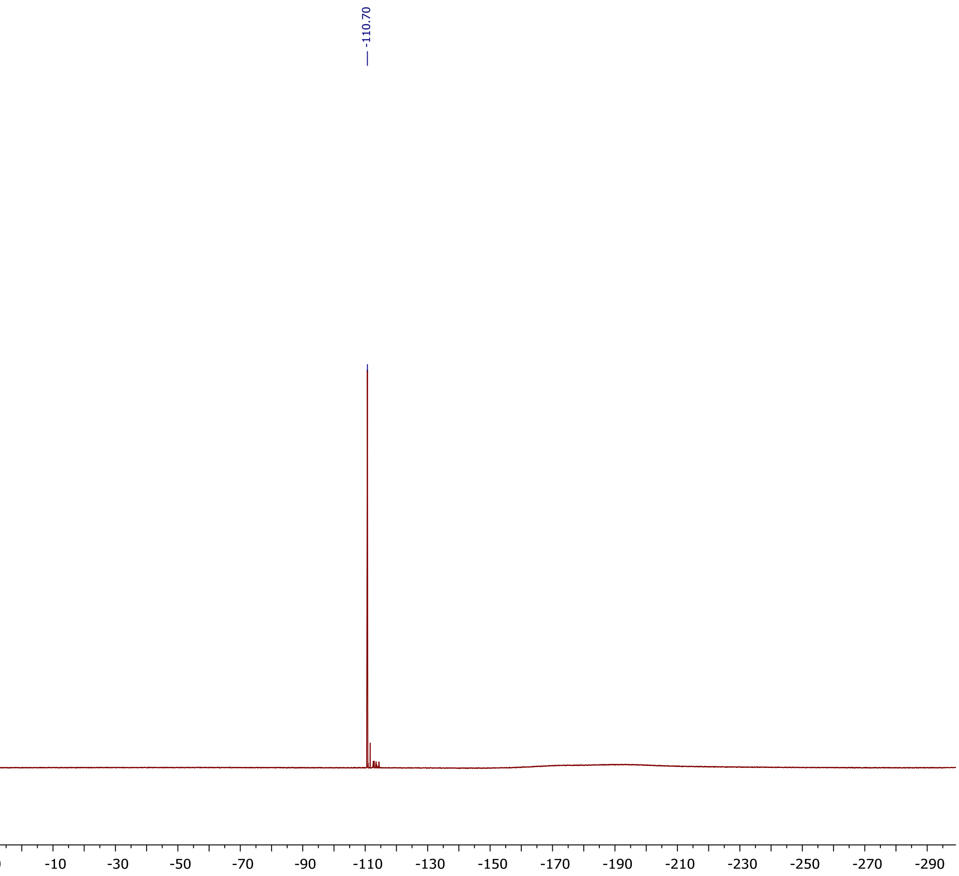
-110.70
90
70
50
30
10
-10
-30
-50
-70
-90
-110
-130
-150
-170
-190
-210
-230
-250
-270
-290

^{1}H, ^{13}C NMR and ^{19}F spectra of compound **4a**

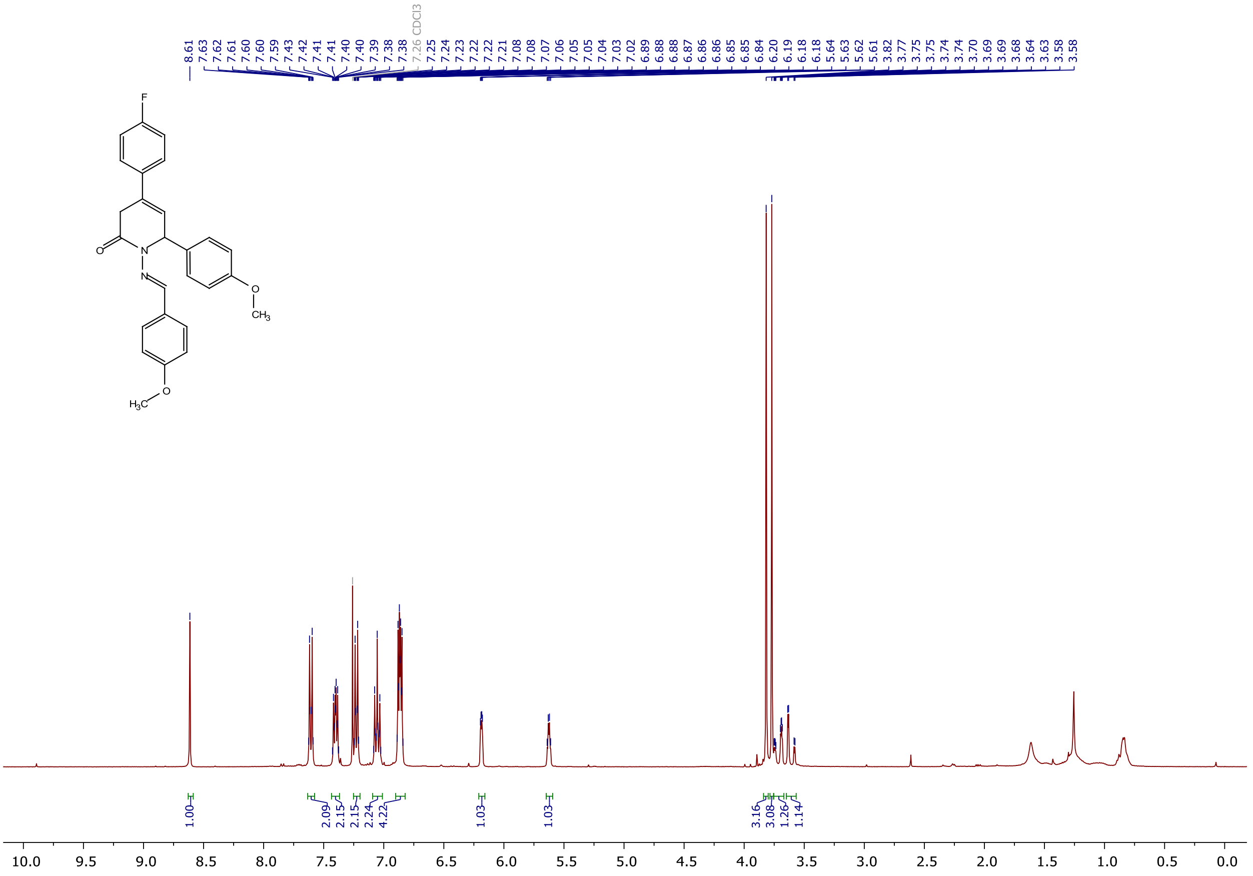

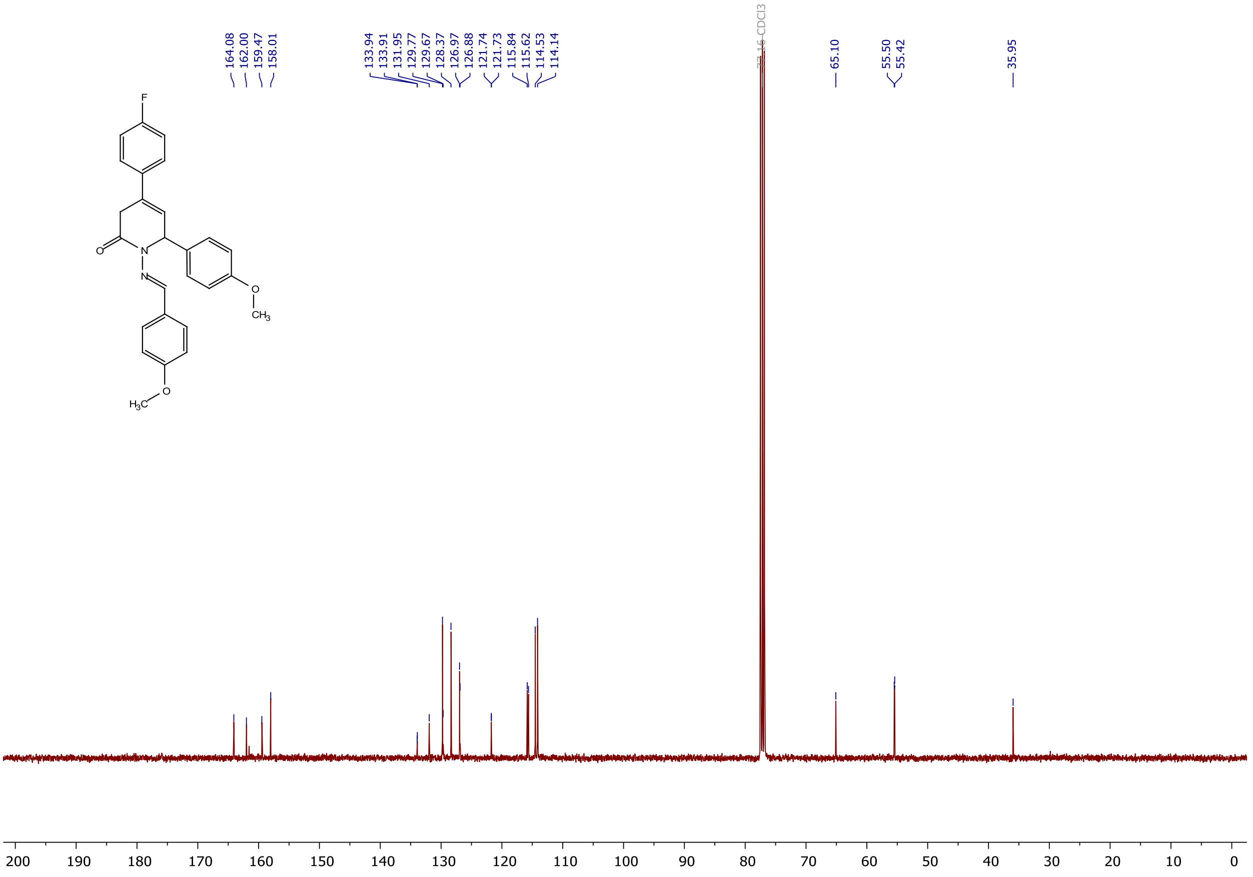

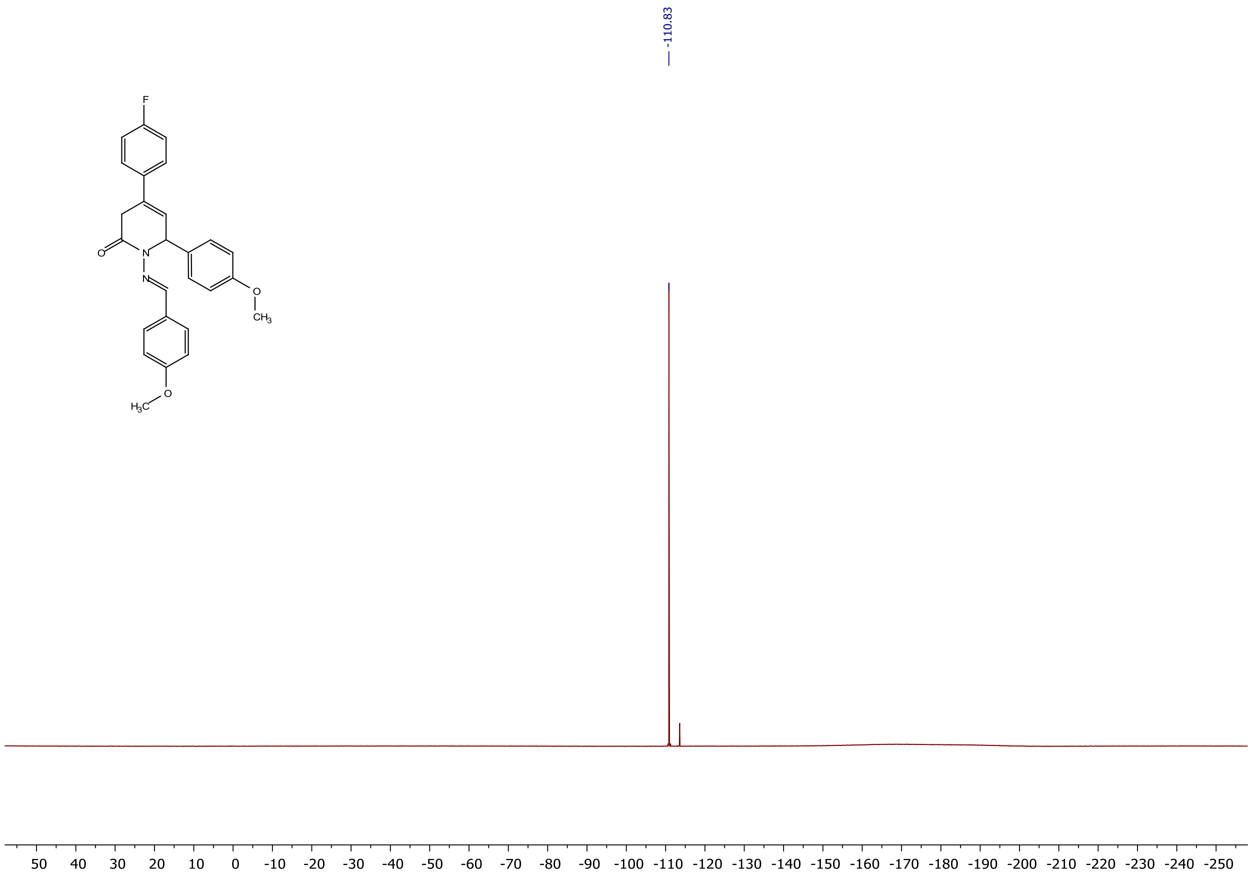

-110.83
F
O
N
N
O
CH3
H3C
50
40
30
20
10
0
-10
-20
-30
-40
-50
-60
-70
-80
-90
-100
-110
-120
-130
-140
-150
-160
-170
-180
-190
-200
-210
-220
-230
-240
-250

^{1}H and ^{13}C NMR spectra of compound **4b**

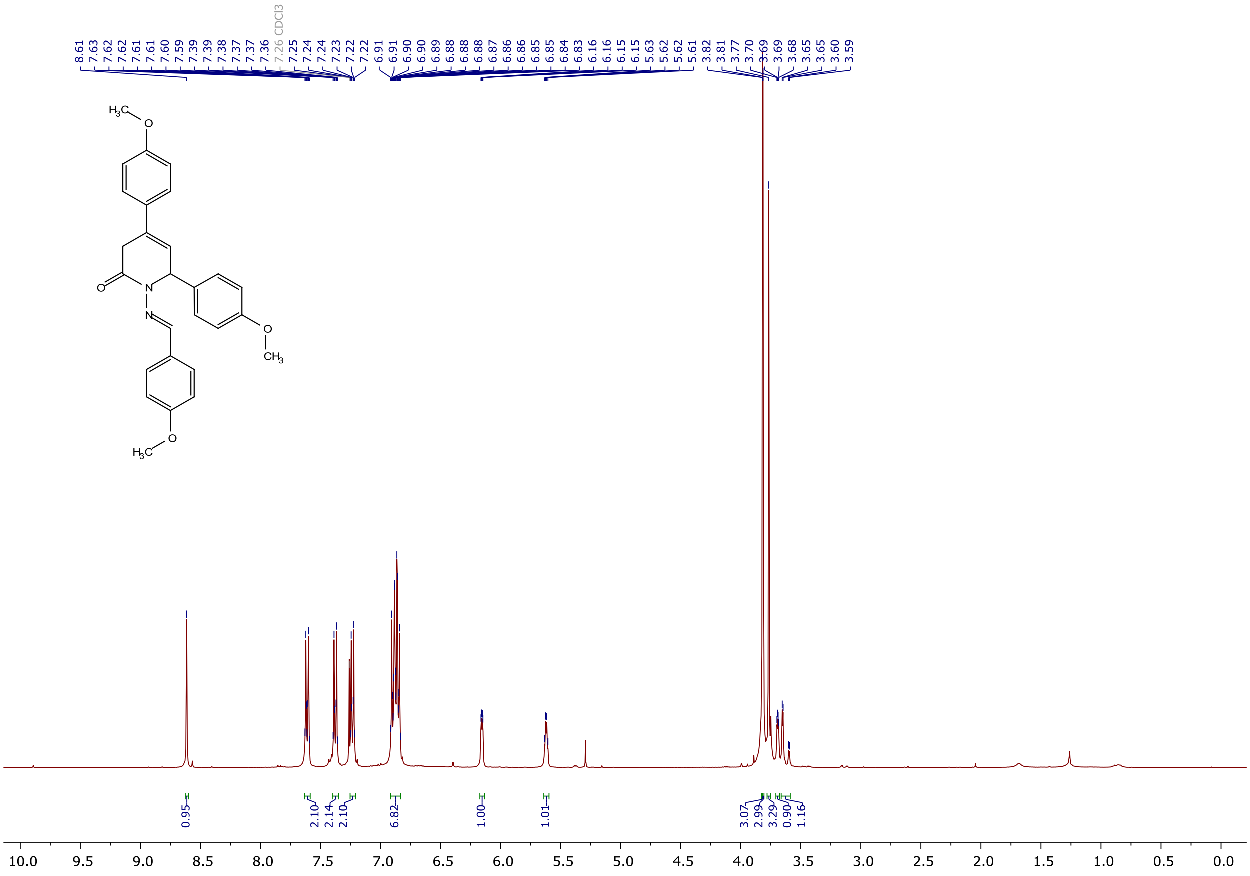

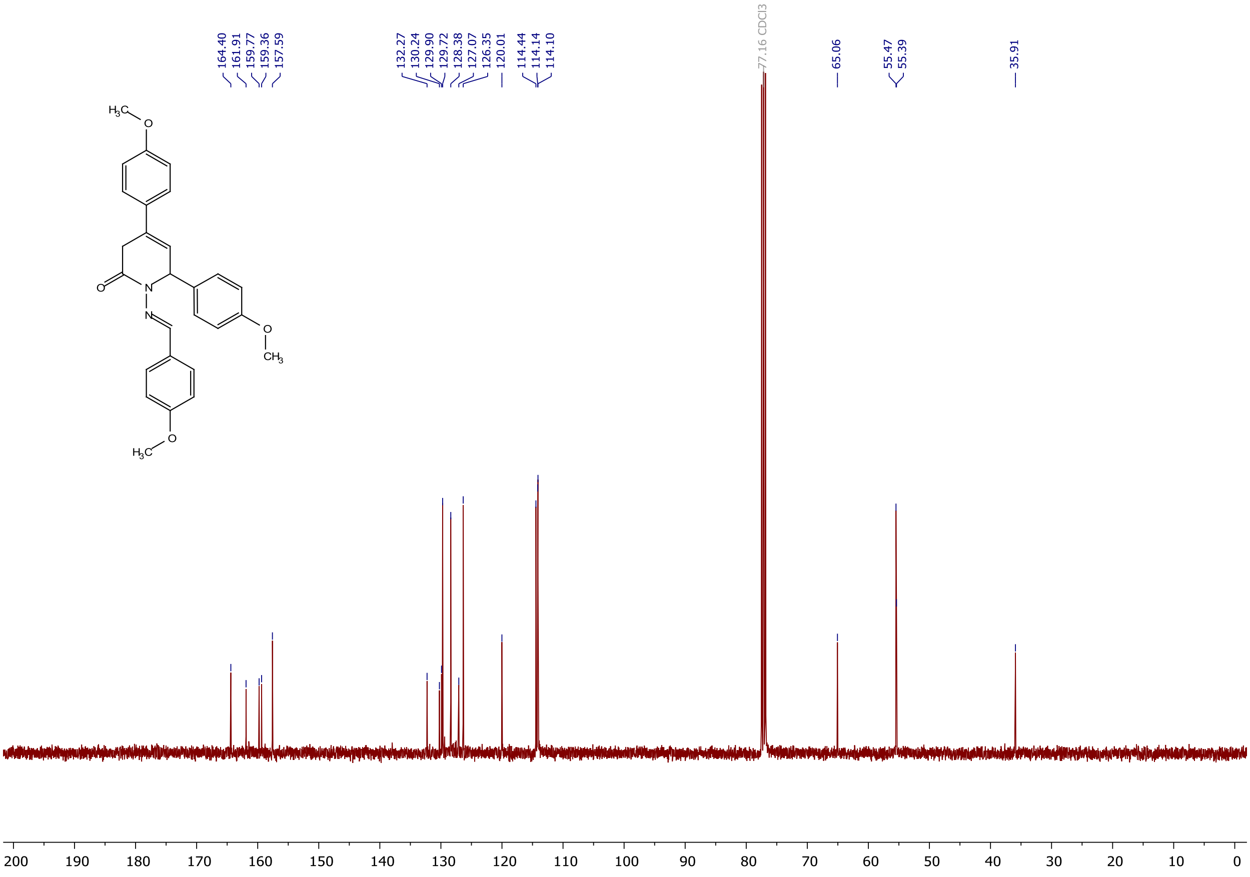

^{1}H and ^{13}C NMR spectra of compound **4c**

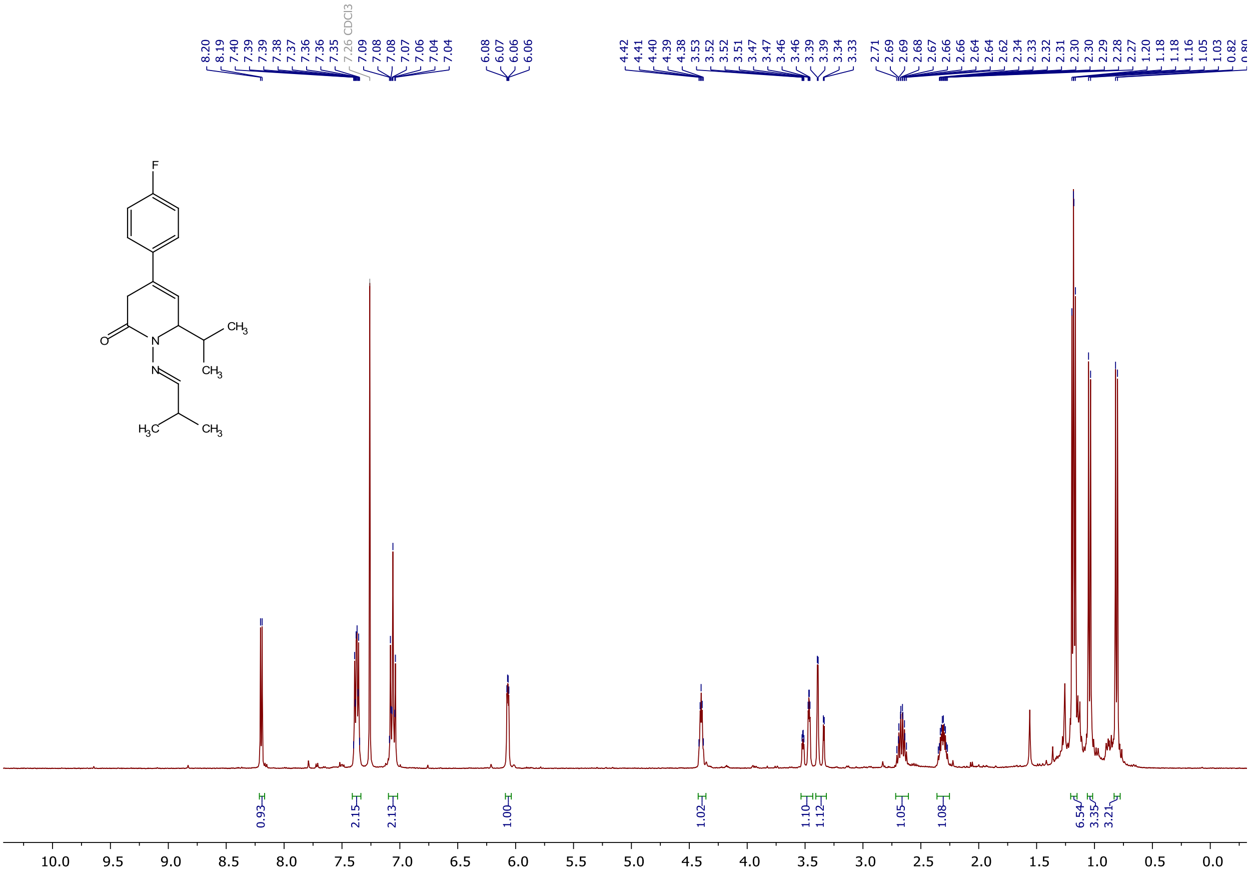

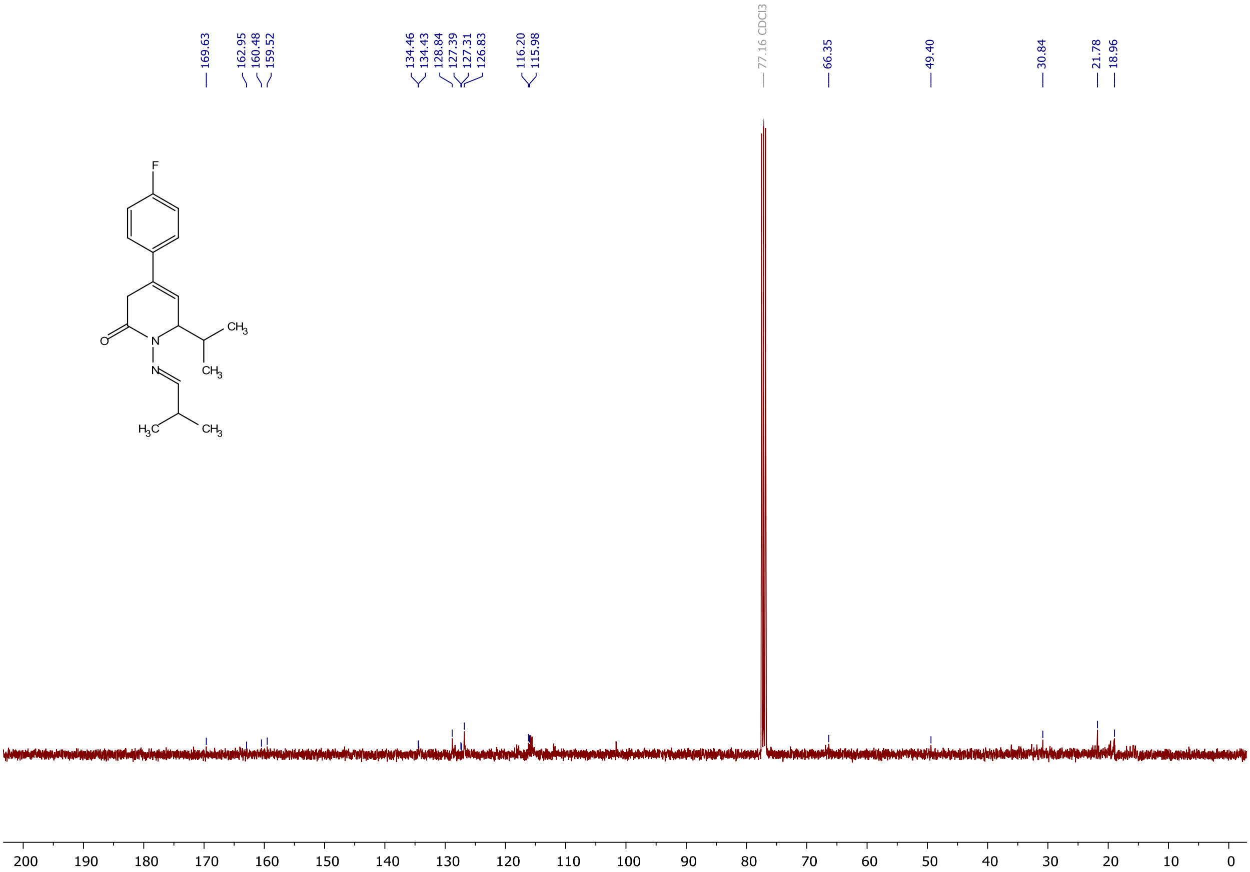

^{1}H and ^{13}C NMR spectra of compound **4d**

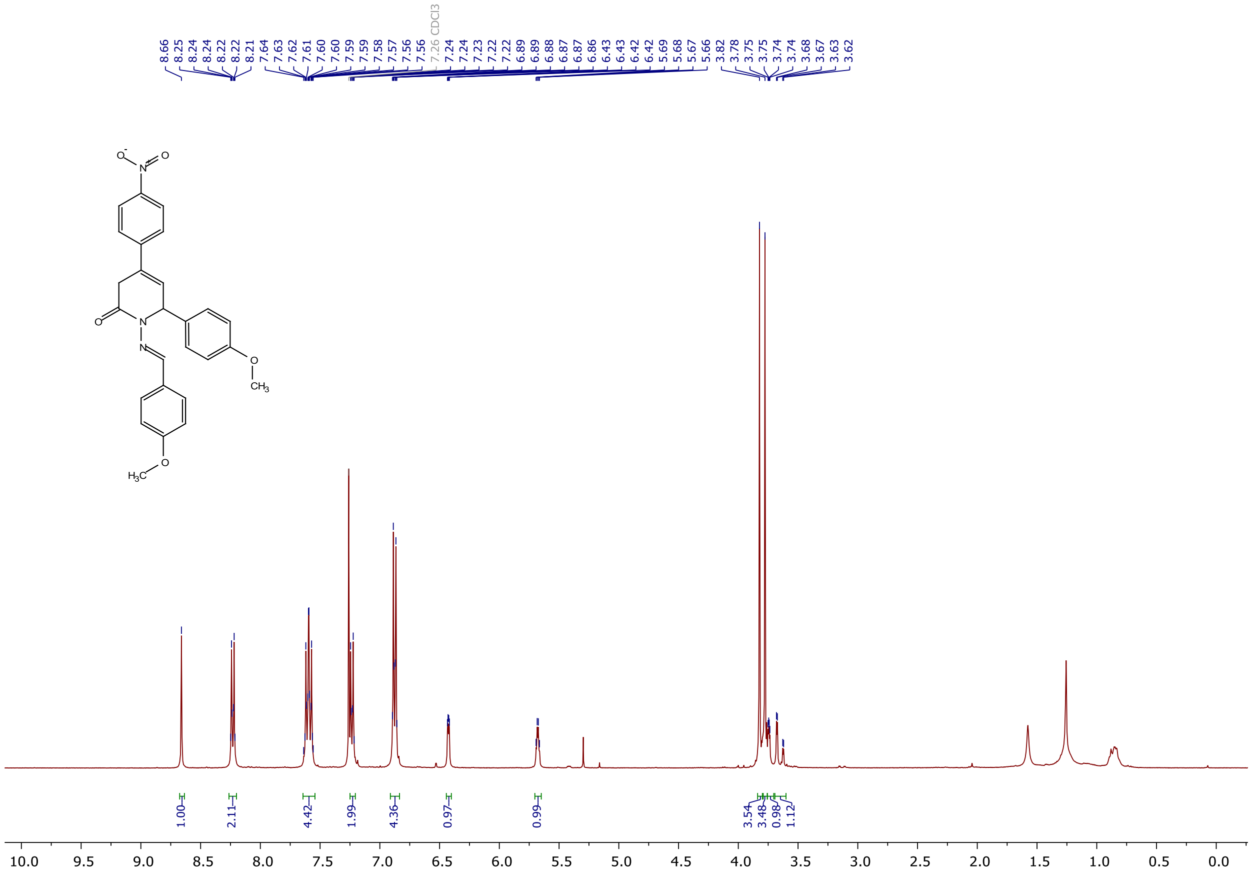

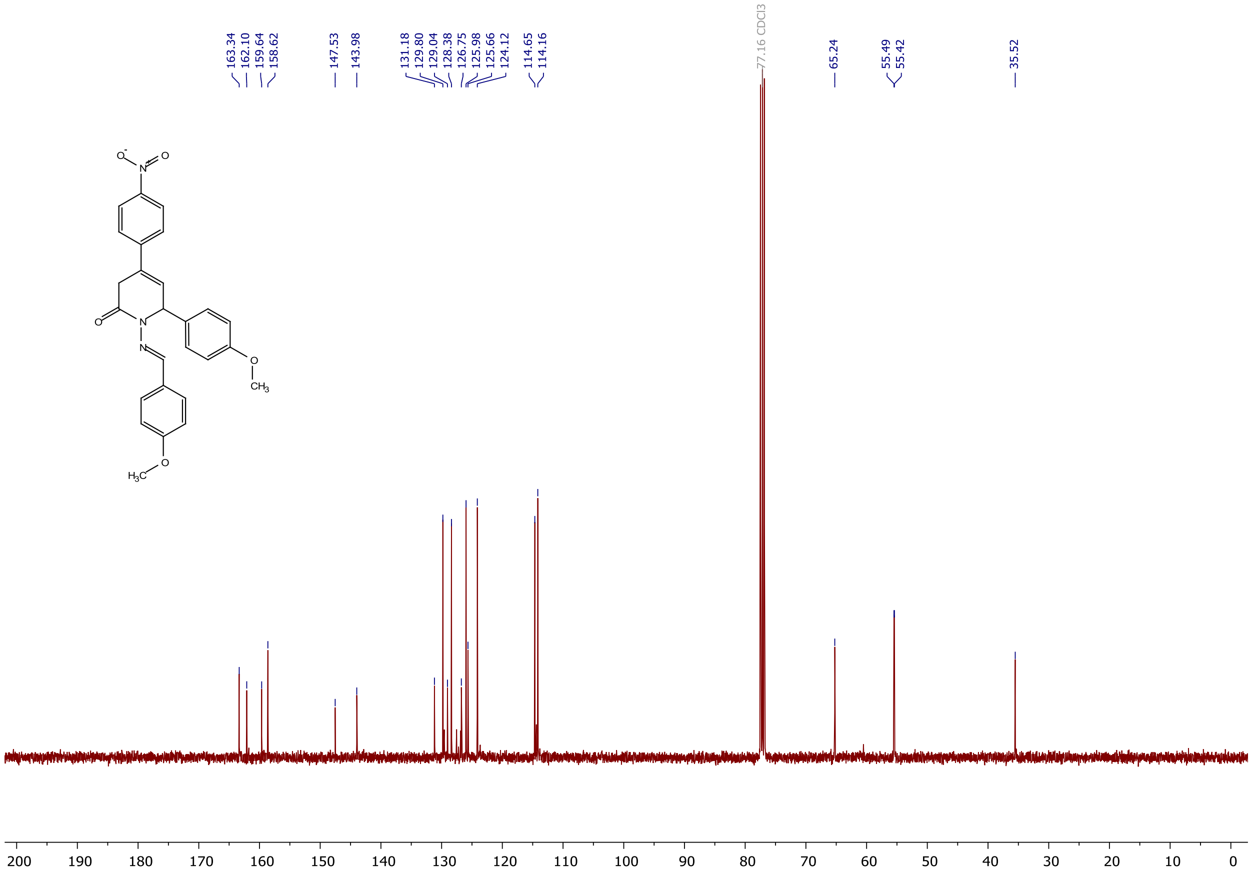

^{1}H and^{13}C NMR and ^{19}F spectra of compound **4e**

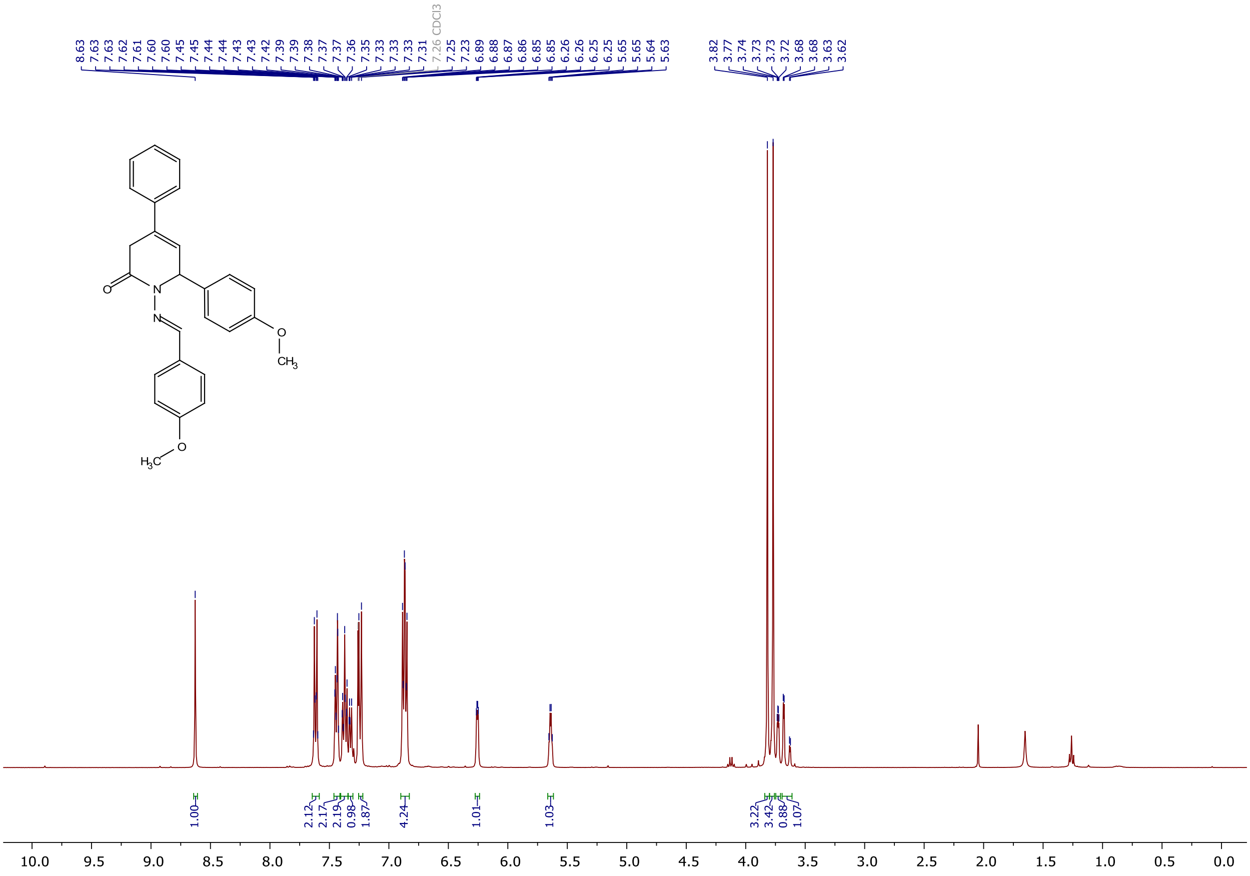

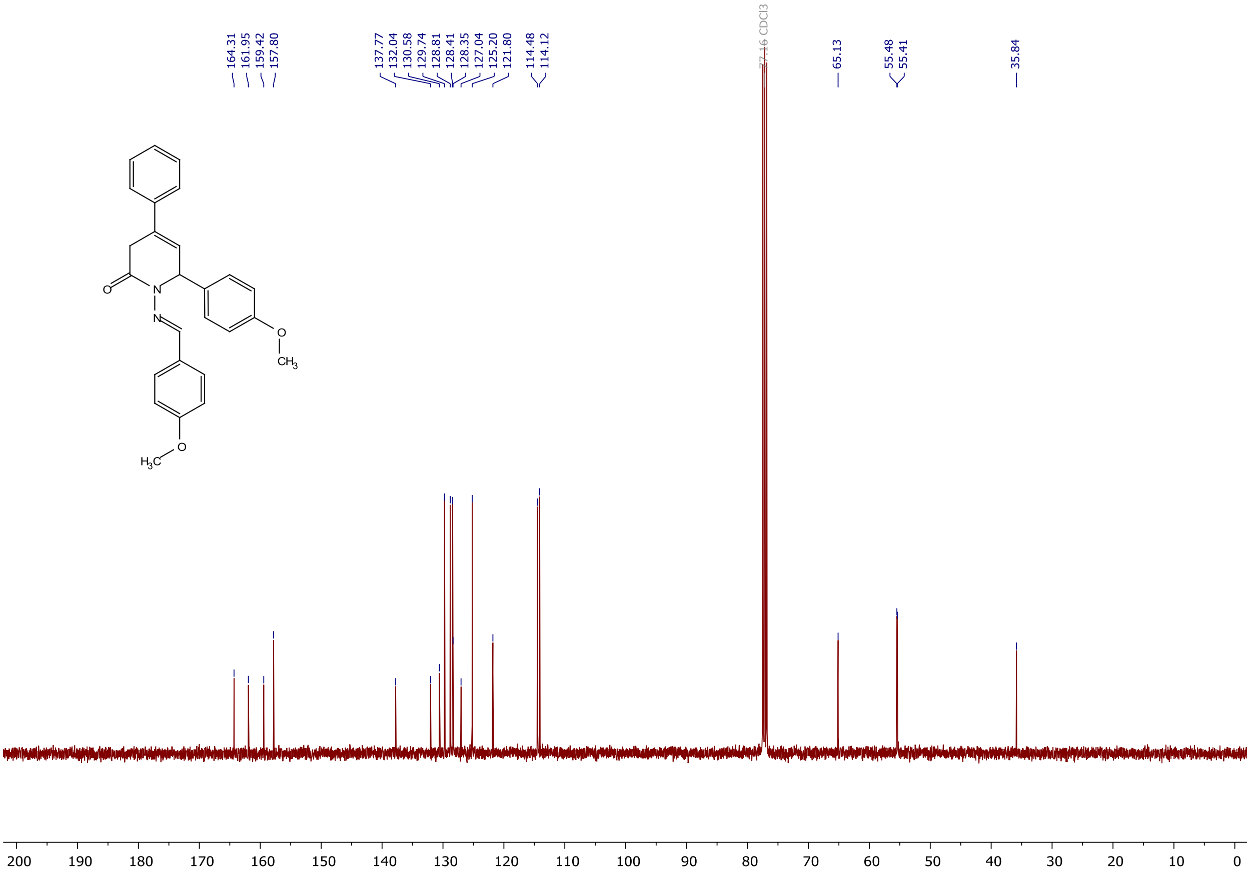

^{1}H, ^{13}C NMR and ^{19}F spectra of compound **4f**

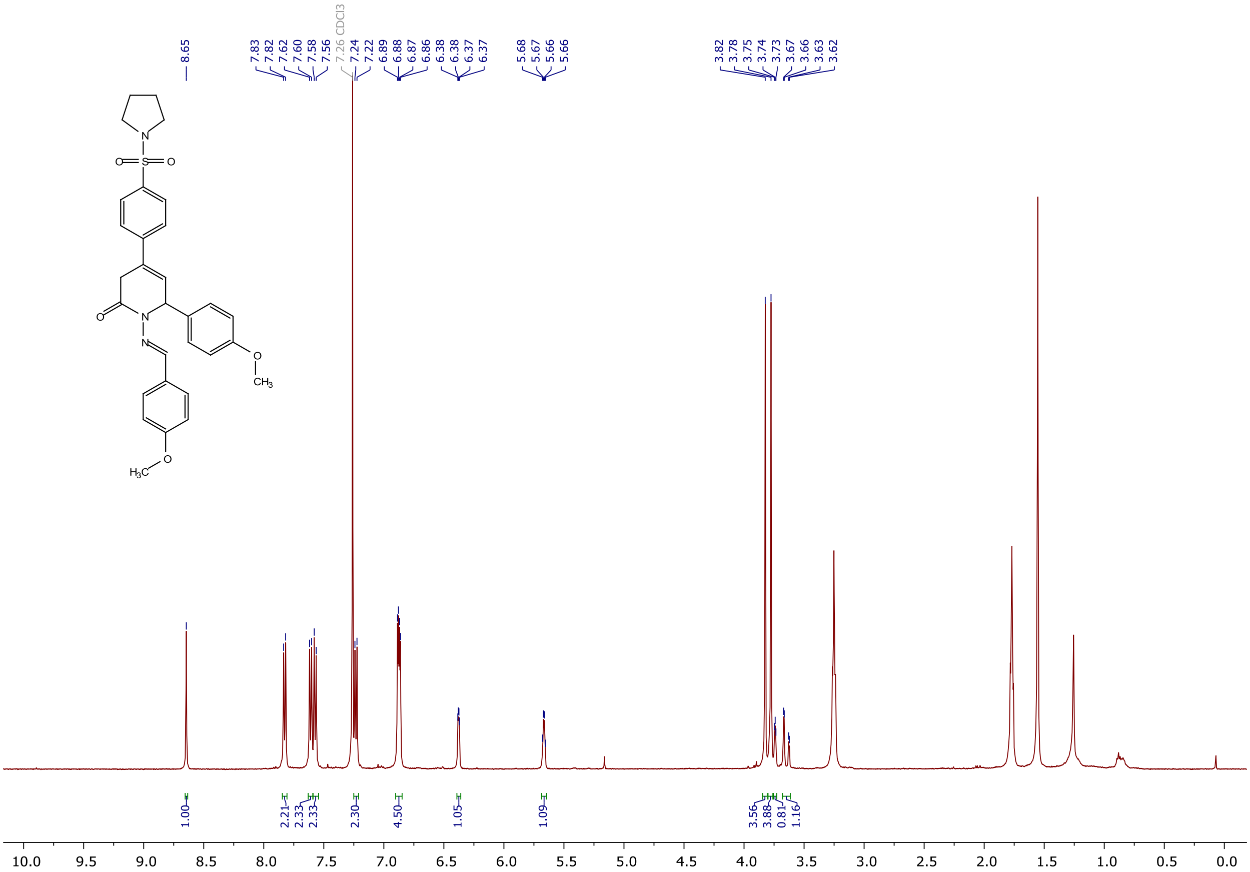

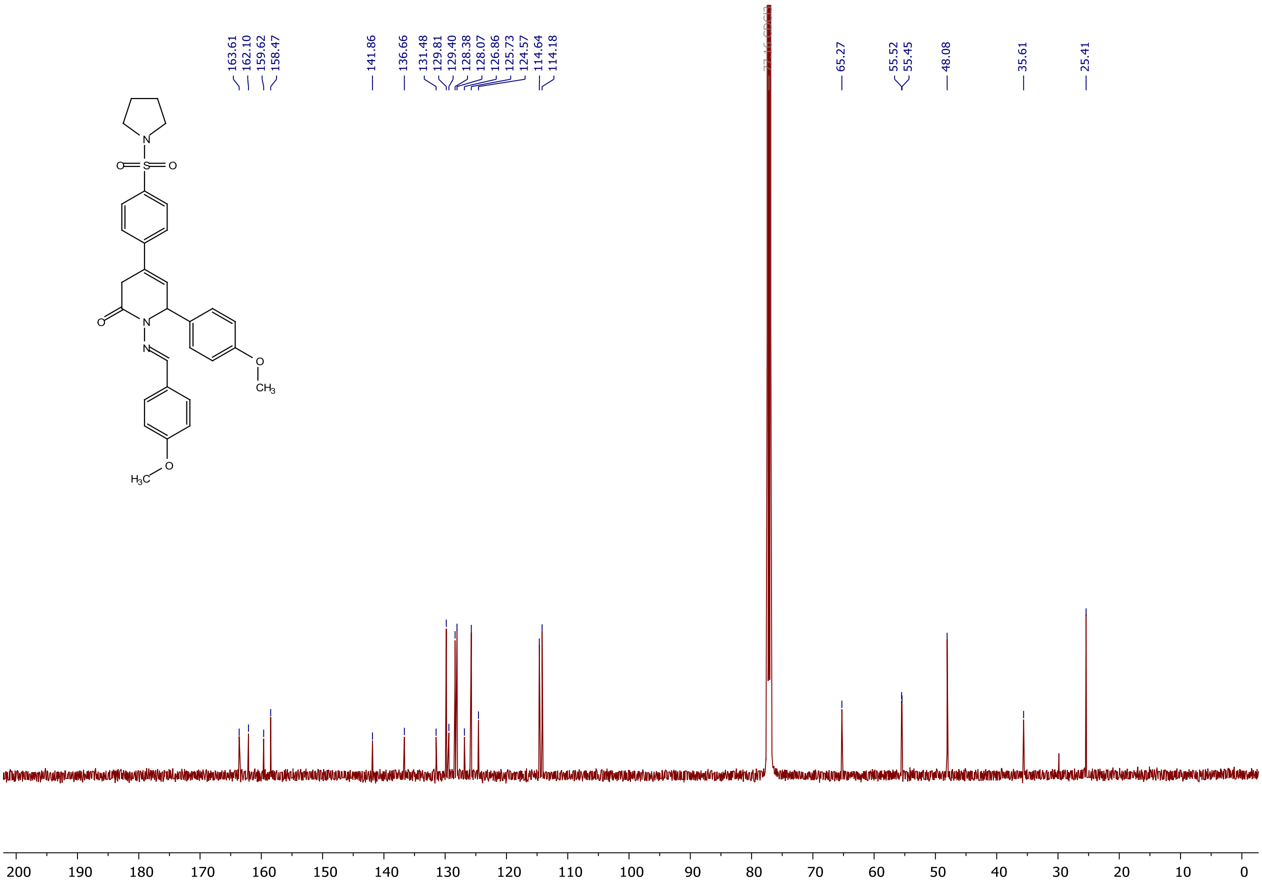

^{1}H and ^{13}C NMR spectra of compound **4g**

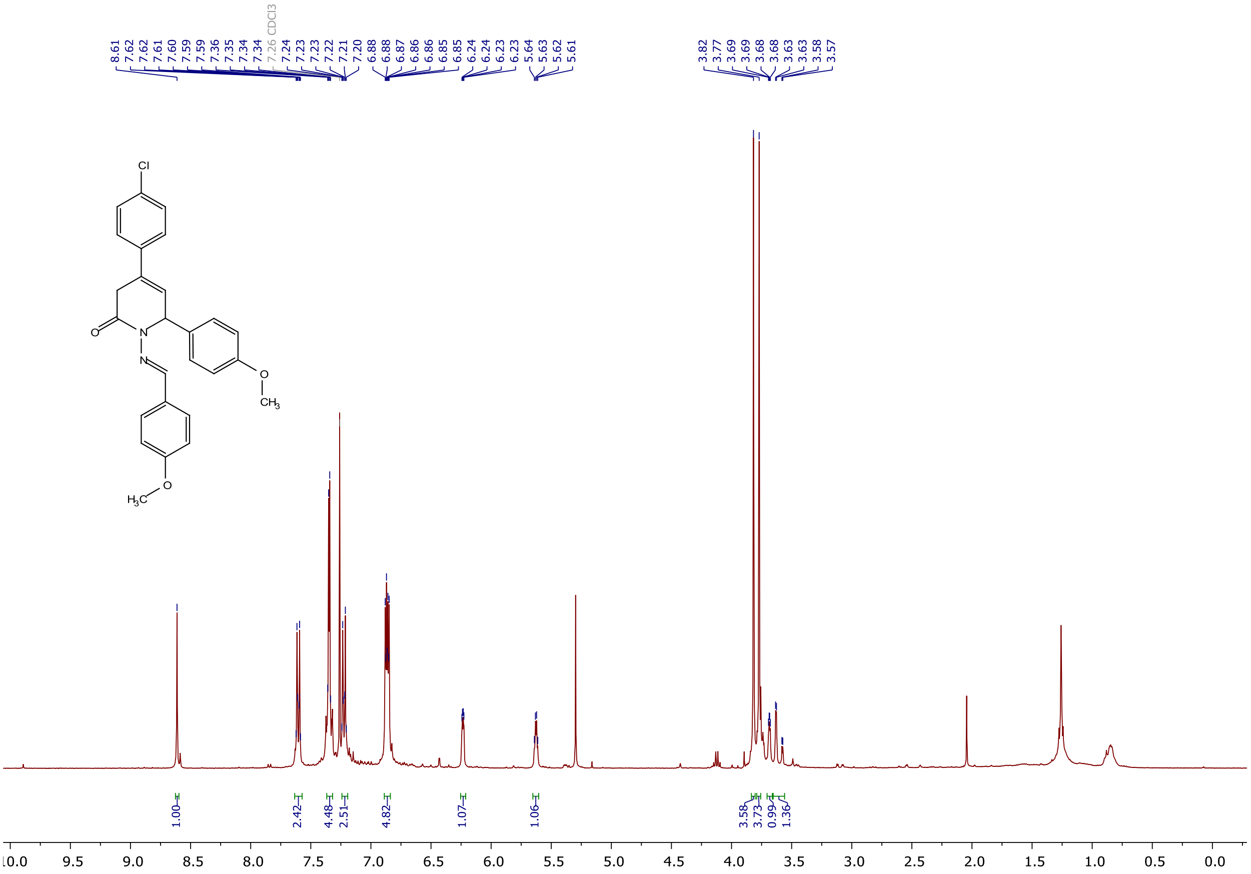

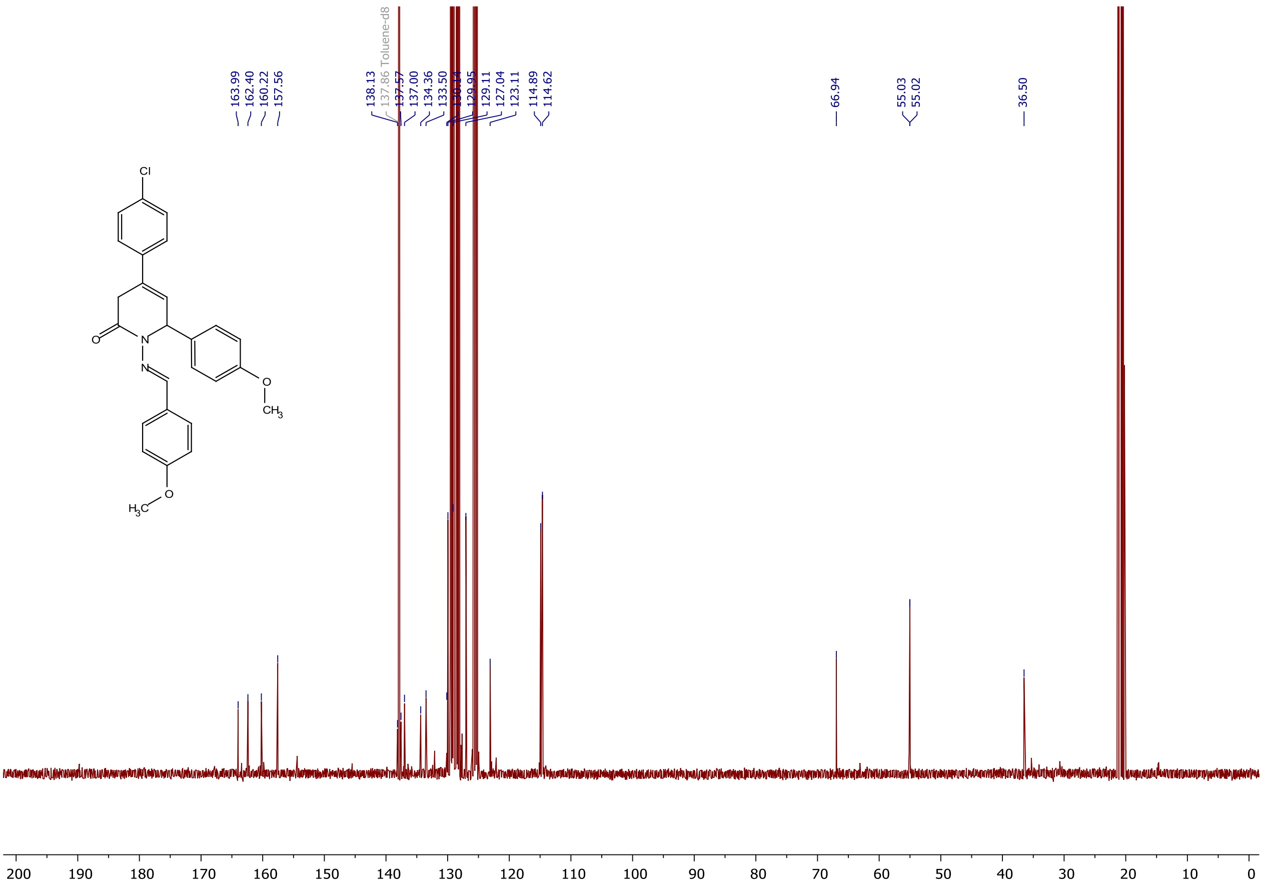

^{1}H, ^{13}C NMR and ^{19}F spectra of compound **4h**

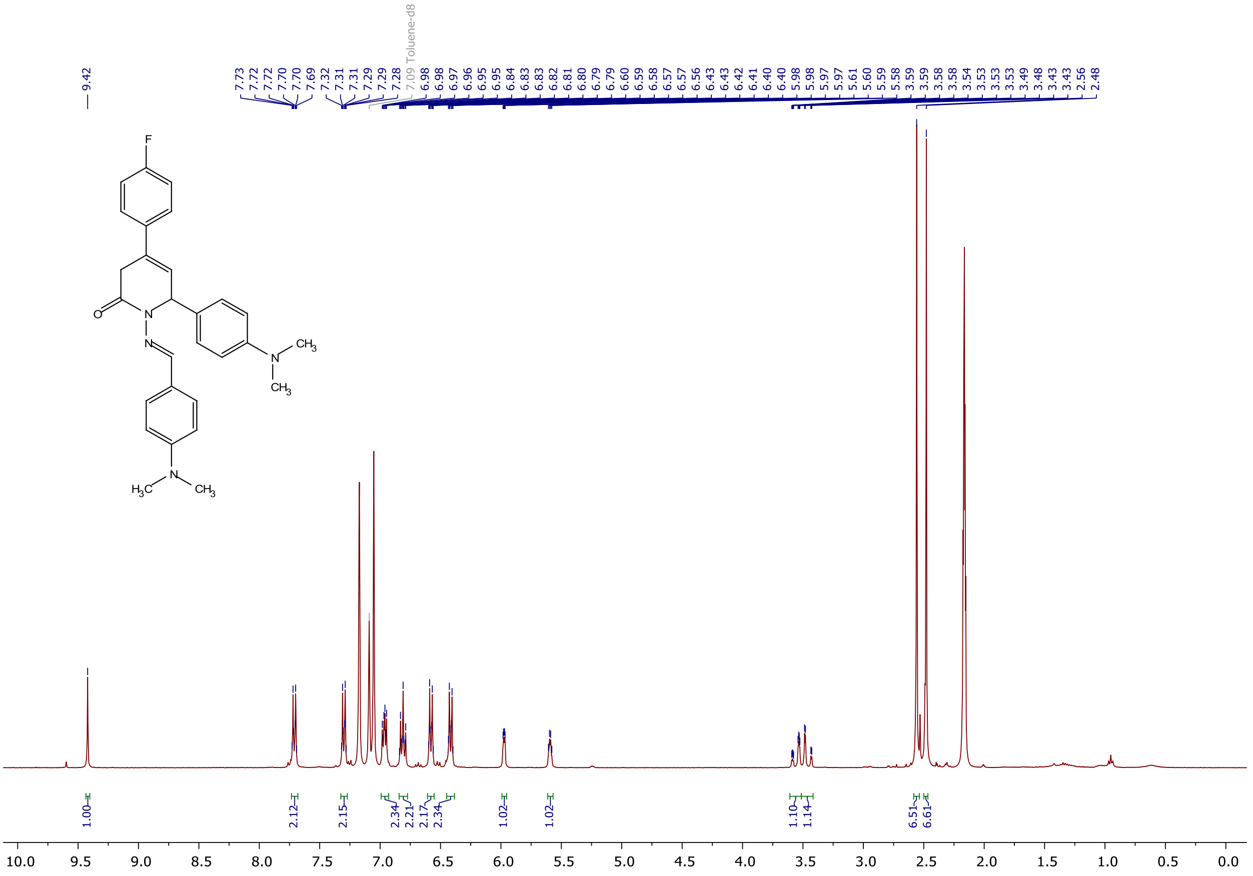

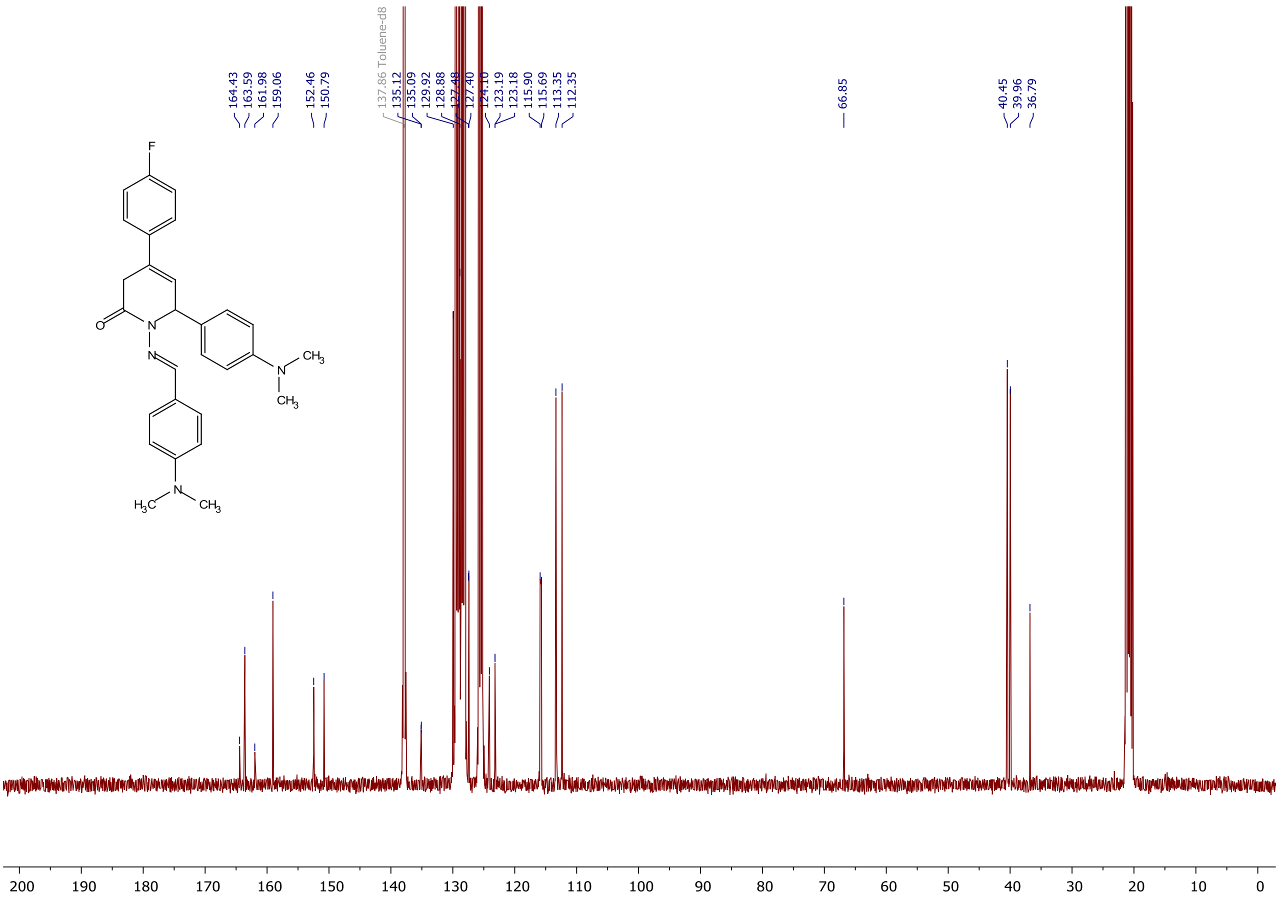

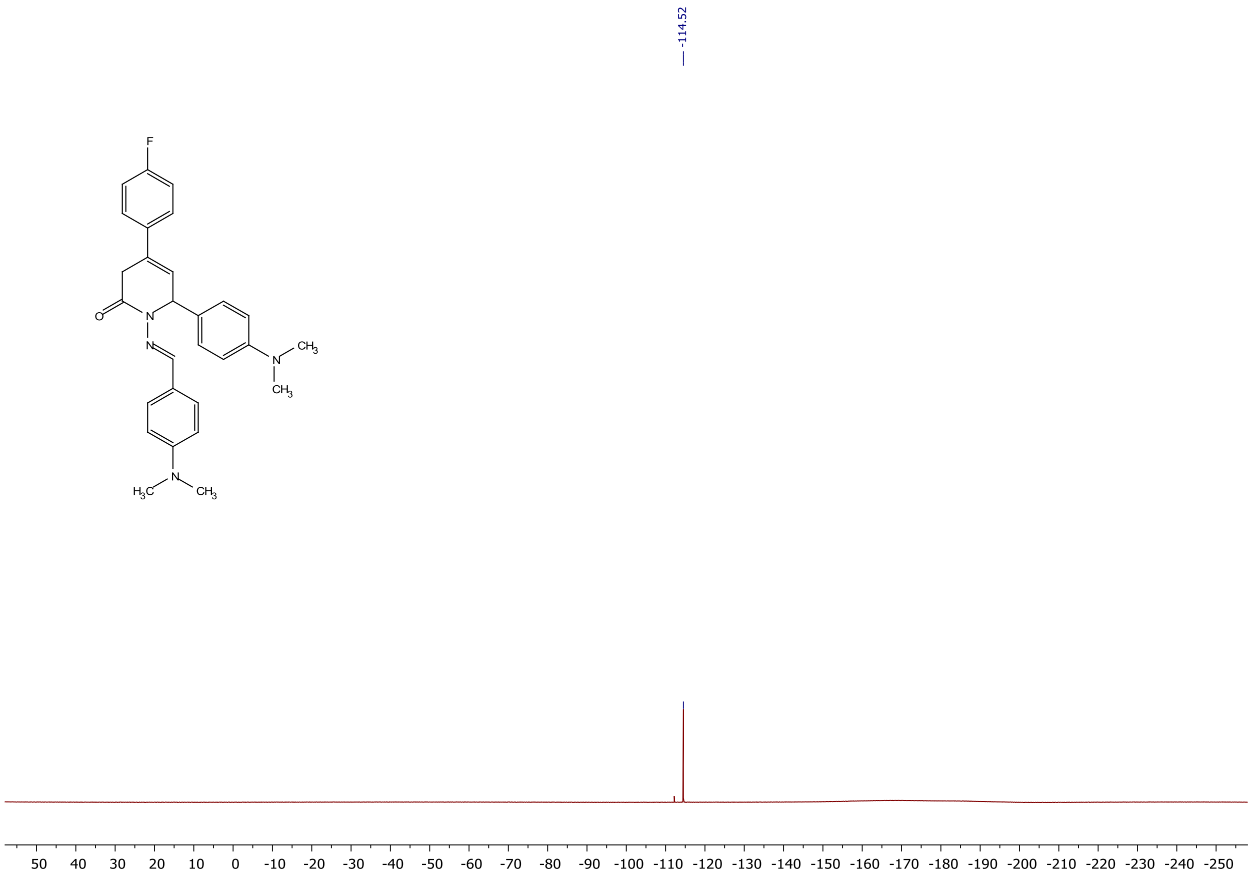
-114.52
F
O
N
N
N
CH3
CH3
N
H3C
CH3
50 40 30 20 10 0 -10 -20 -30 -40 -50 -60 -70 -80 -90 -100 -110 -120 -130 -140 -150 -160 -170 -180 -190 -200 -210 -220 -230 -240 -250

^{1}H, ^{13}C NMR and ^{19}F spectra of compound **4i**

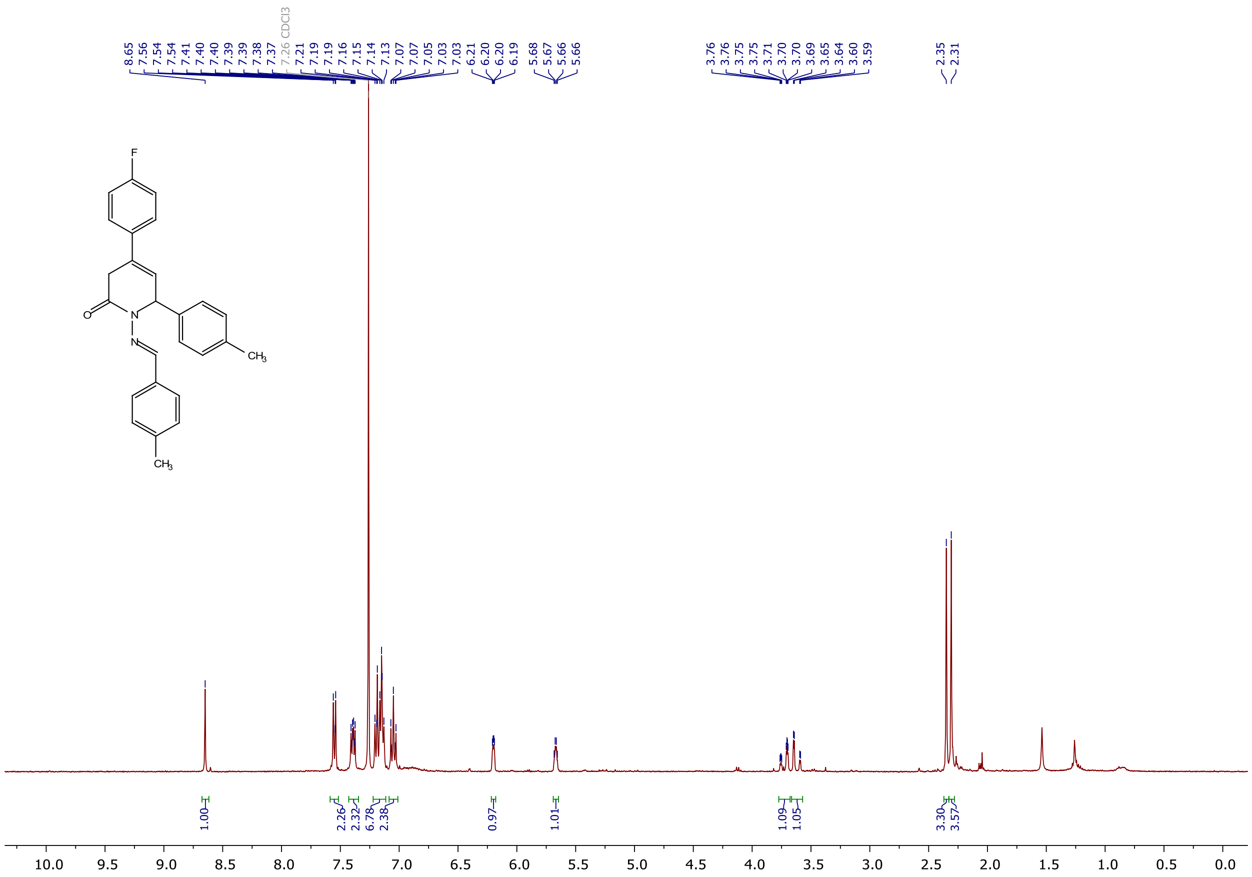

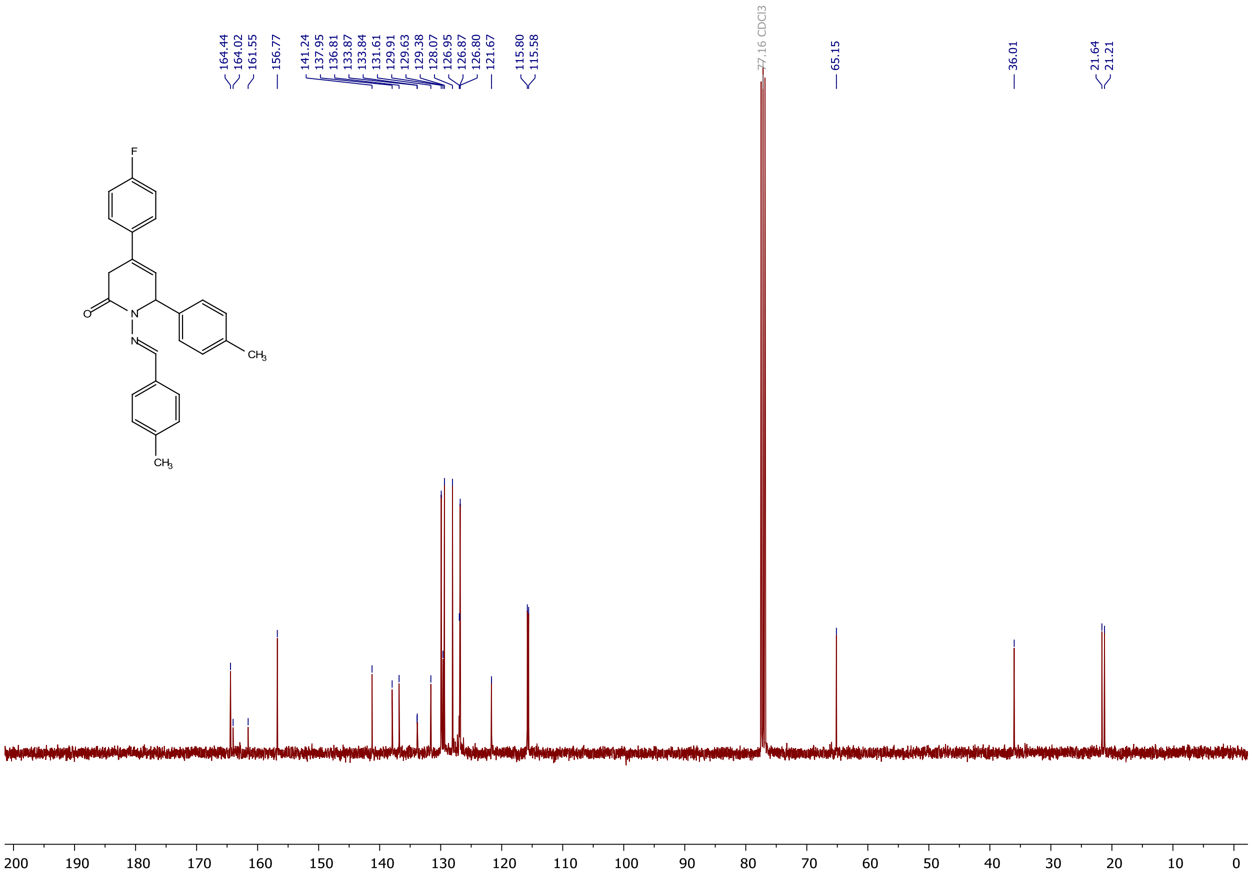

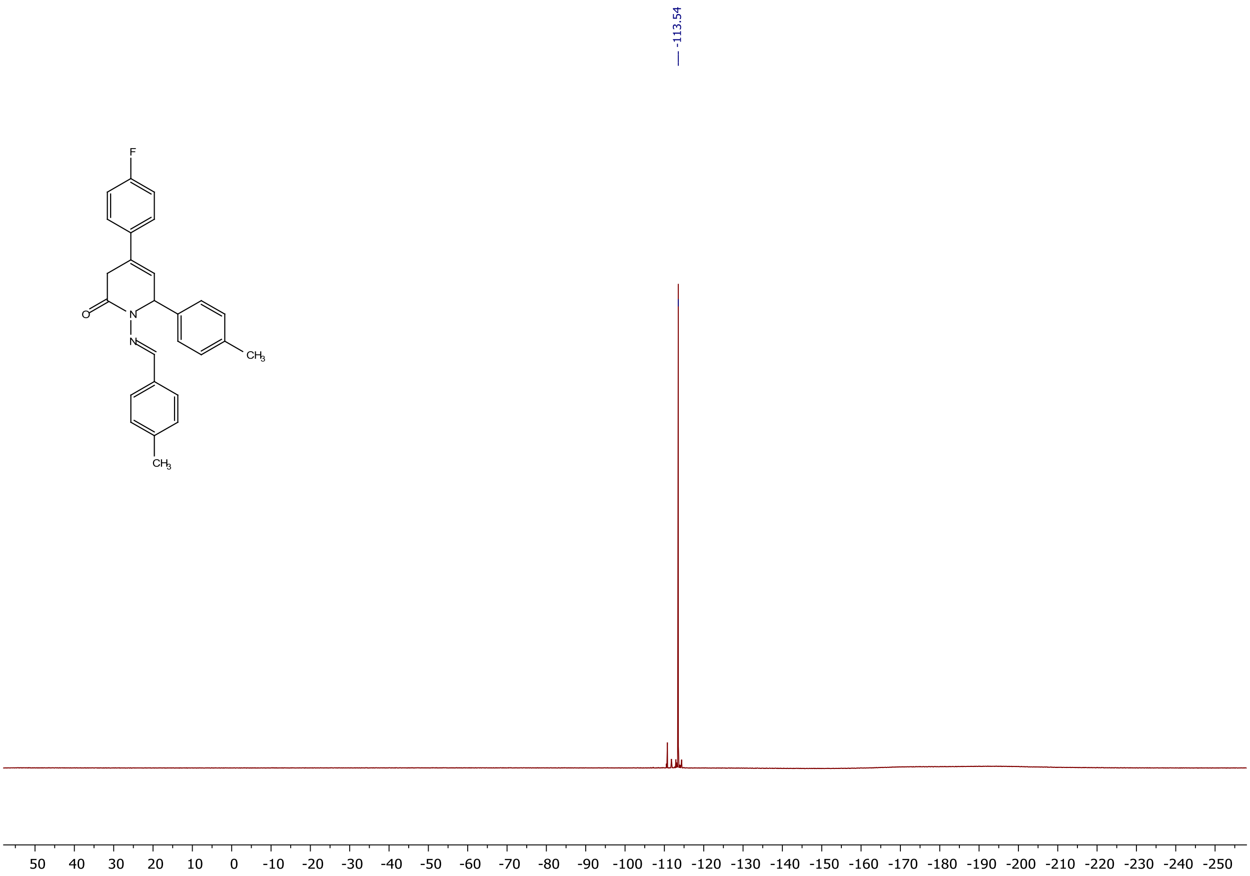
-113.54
F
O
N
N
CH3
CH3
50 40 30 20 10 0 -10 -20 -30 -40 -50 -60 -70 -80 -90 -100 -110 -120 -130 -140 -150 -160 -170 -180 -190 -200 -210 -220 -230 -240 -250

^{1}H, ^{13}C NMR and ^{19}F spectra of compound **4j**

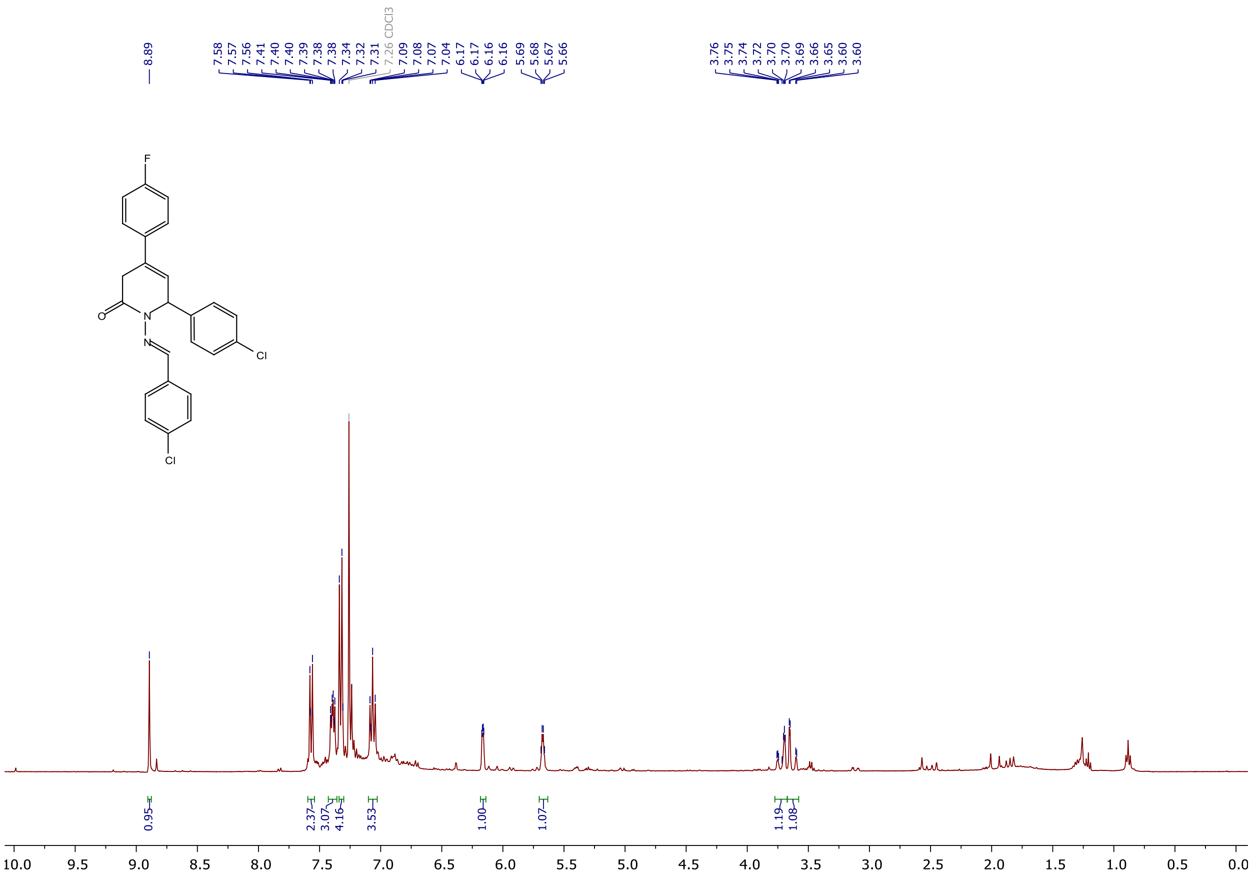

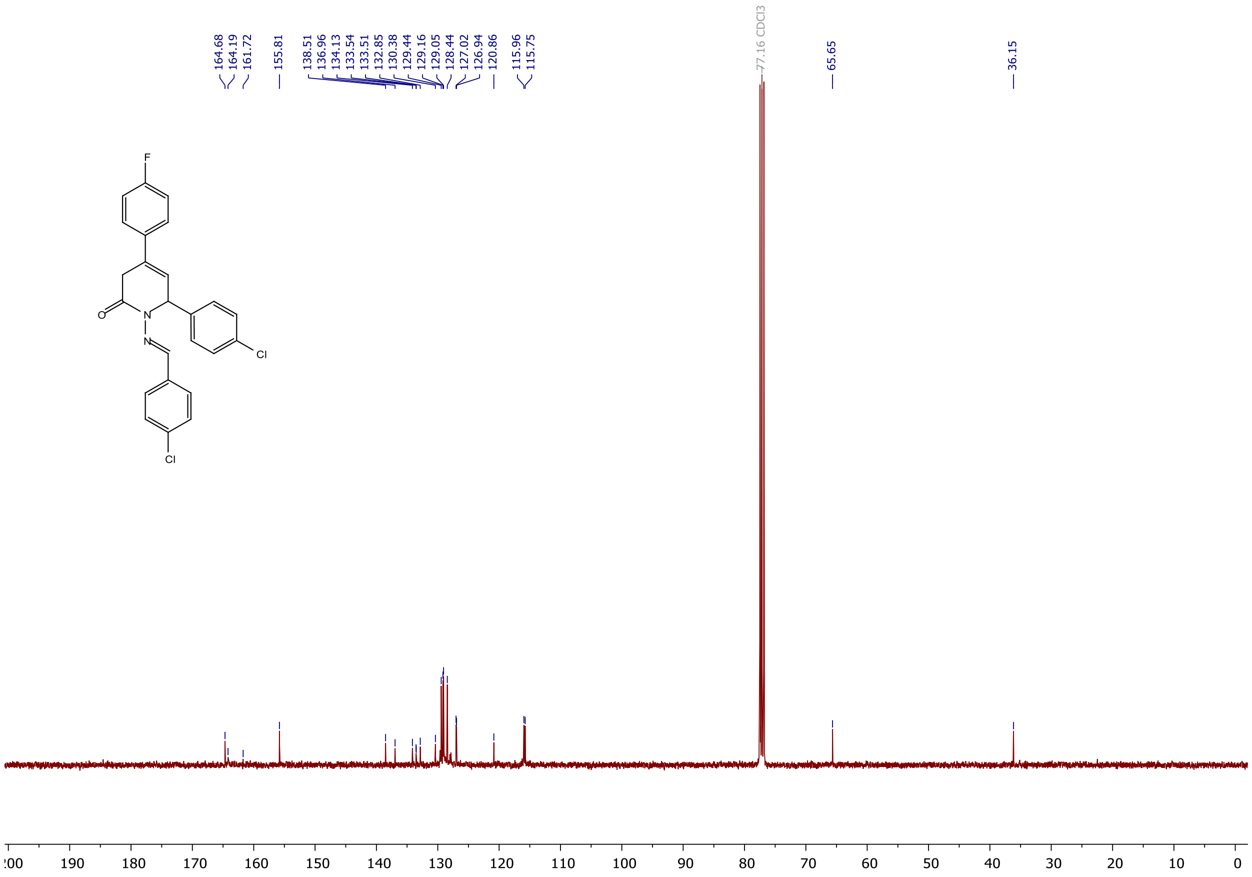

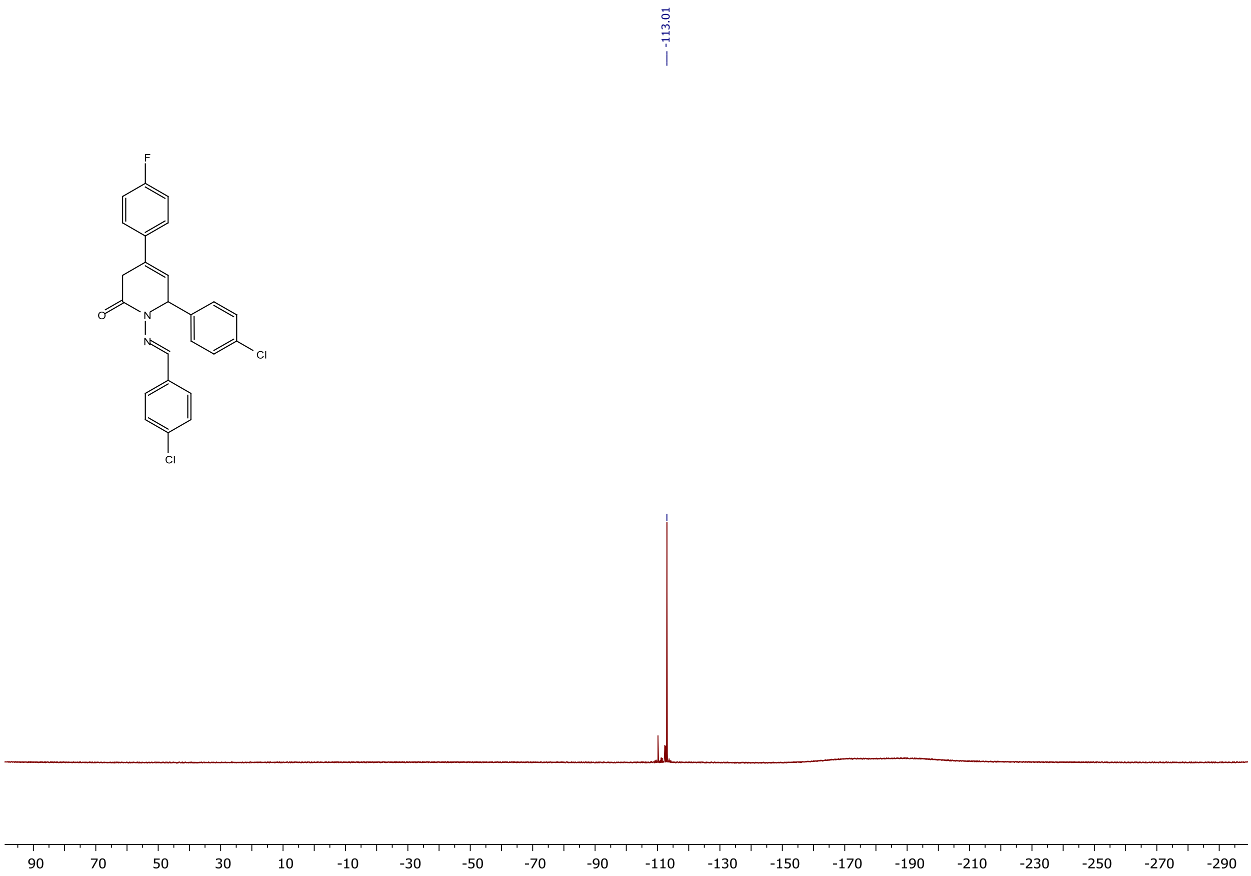
-113.01
F
O
N
N
Cl
Cl
90 70 50 30 10 -10 -30 -50 -70 -90 -110 -130 -150 -170 -190 -210 -230 -250 -270 -290

^{1}H, ^{13}C NMR and ^{19}F spectra of compound **4aa**

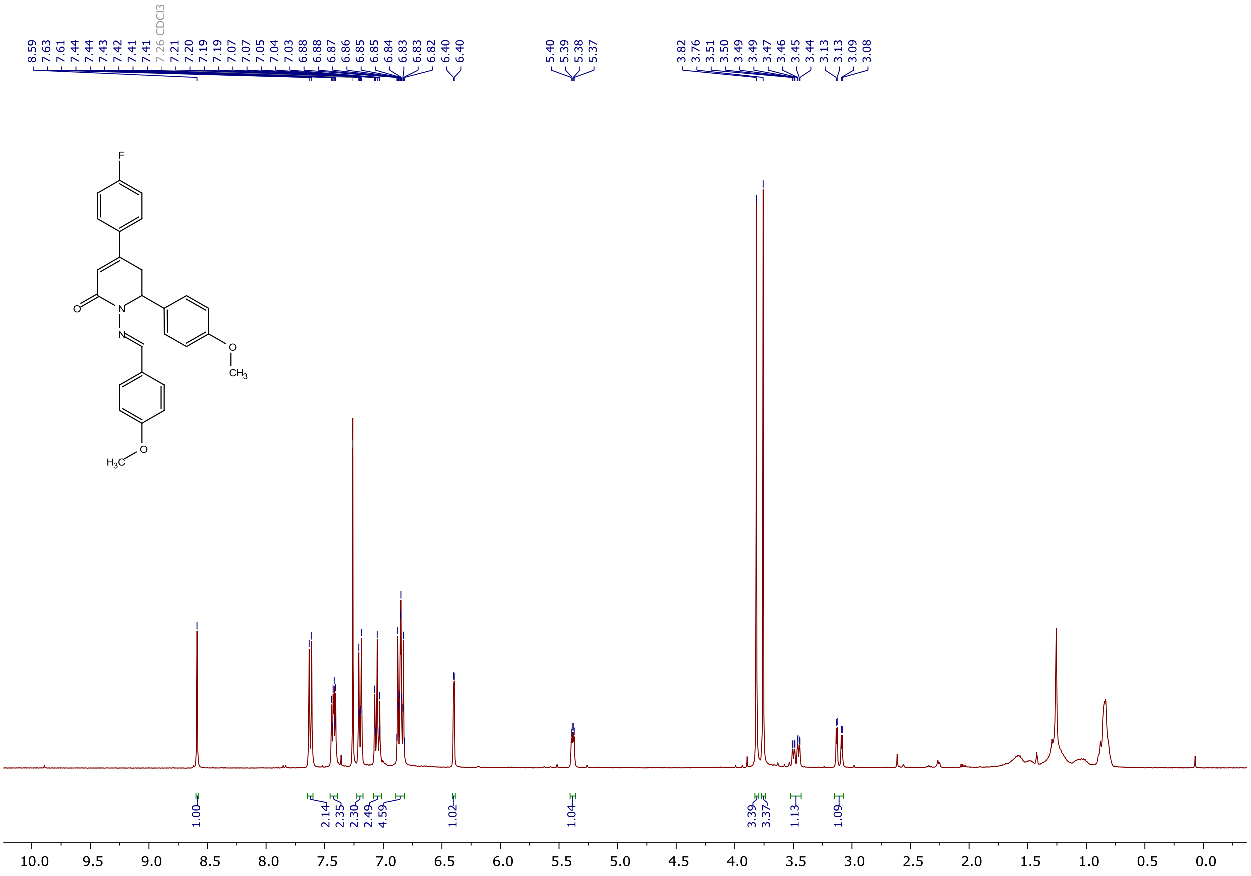

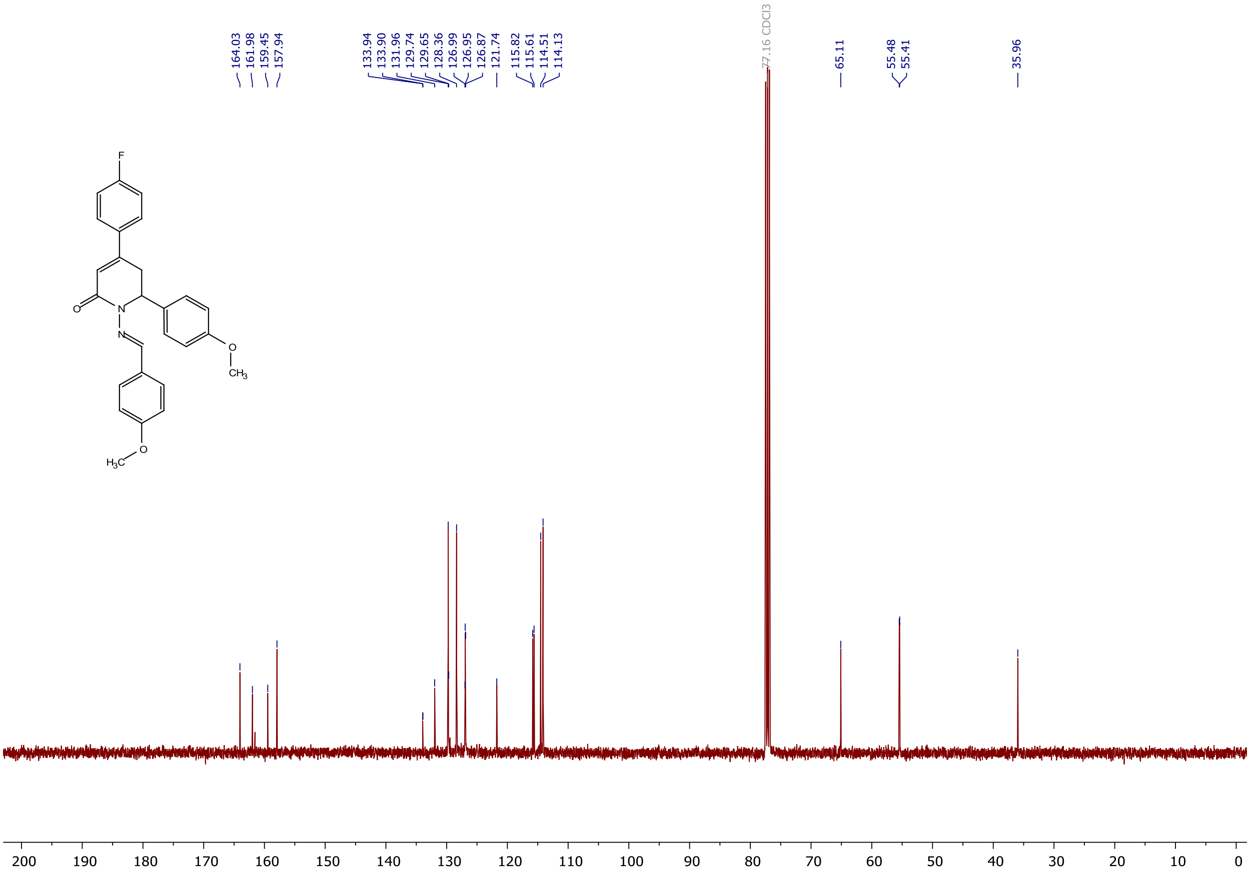

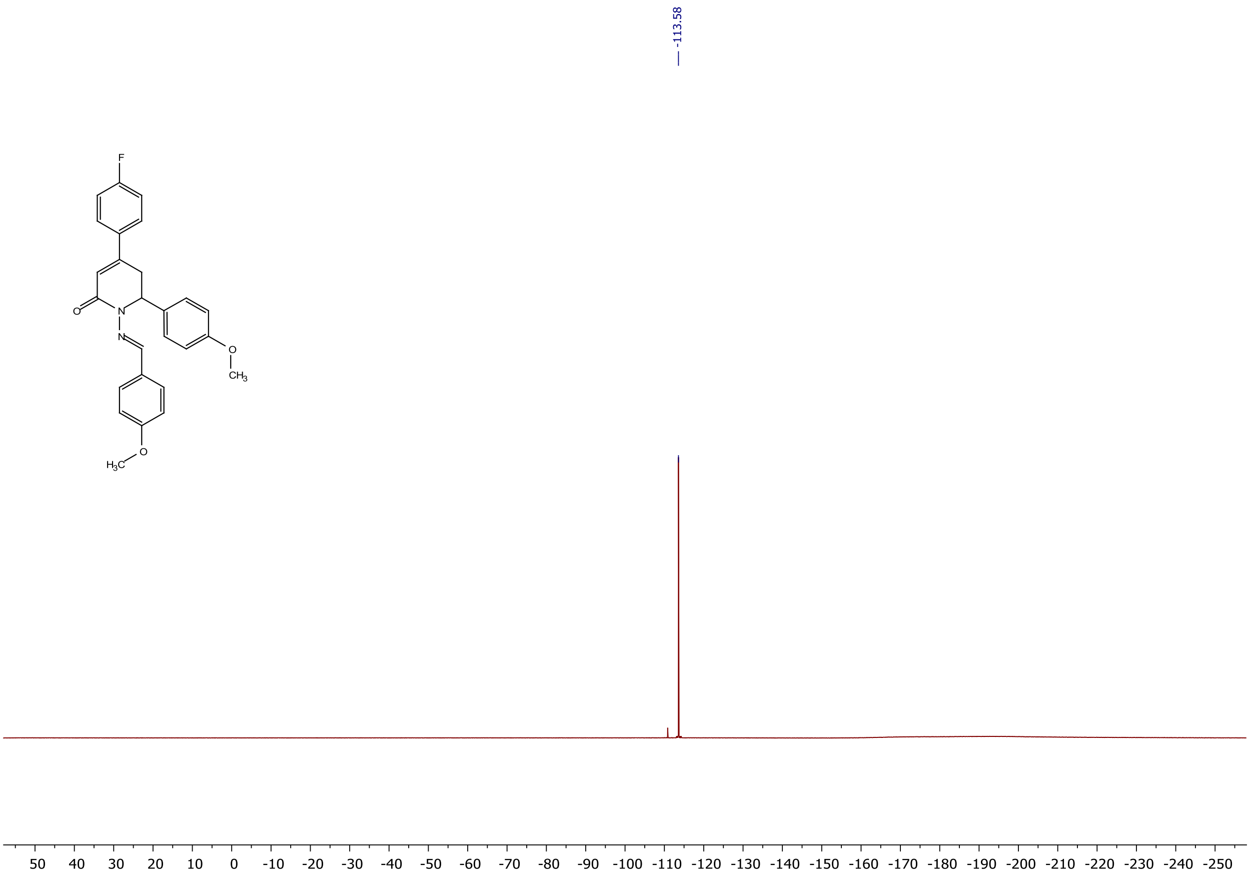
-113.58
F
O
N
N
O
CH3
H3C
O
50 40 30 20 10 0 -10 -20 -30 -40 -50 -60 -70 -80 -90 -100 -110 -120 -130 -140 -150 -160 -170 -180 -190 -200 -210 -220 -230 -240 -250

^{1}H, ^{13}C NMR and ^{19}F spectra of compound **5**

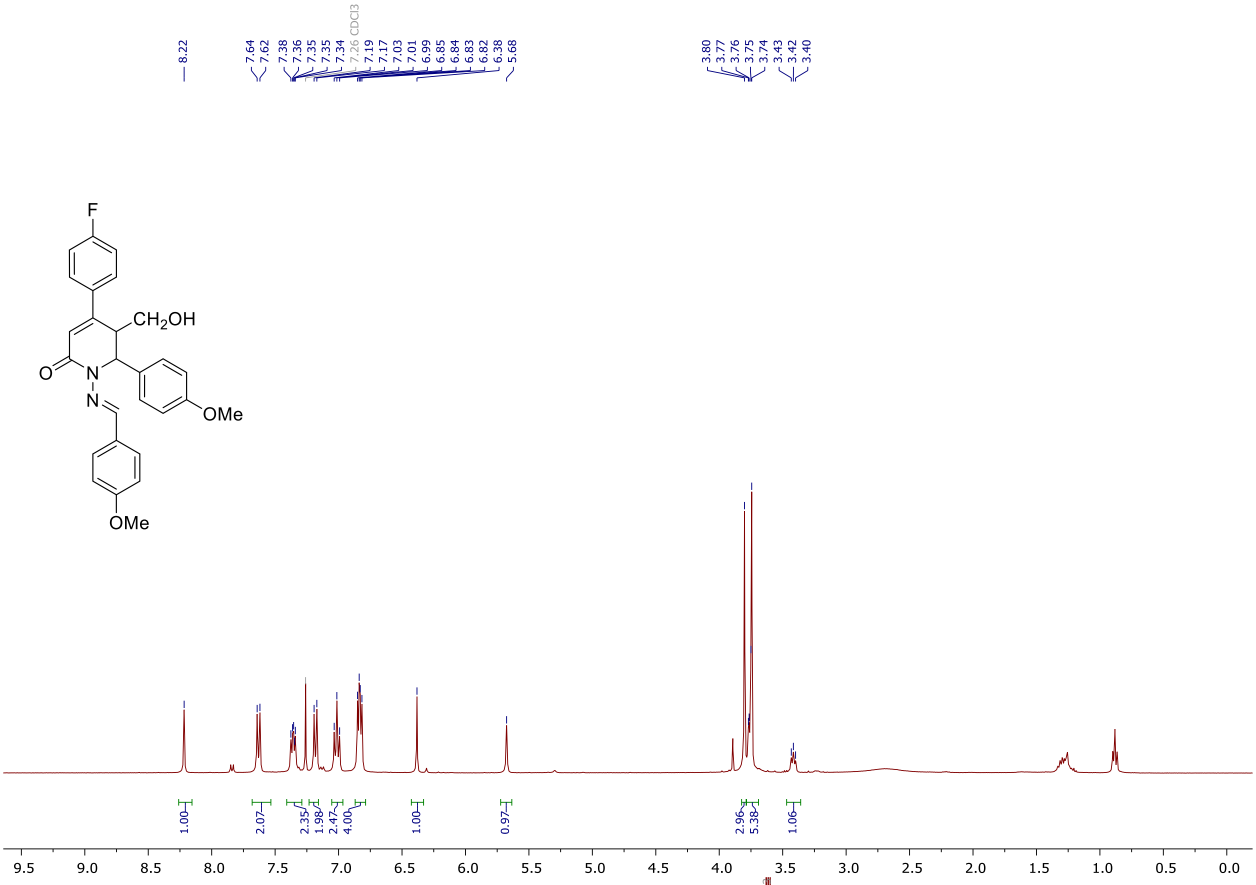

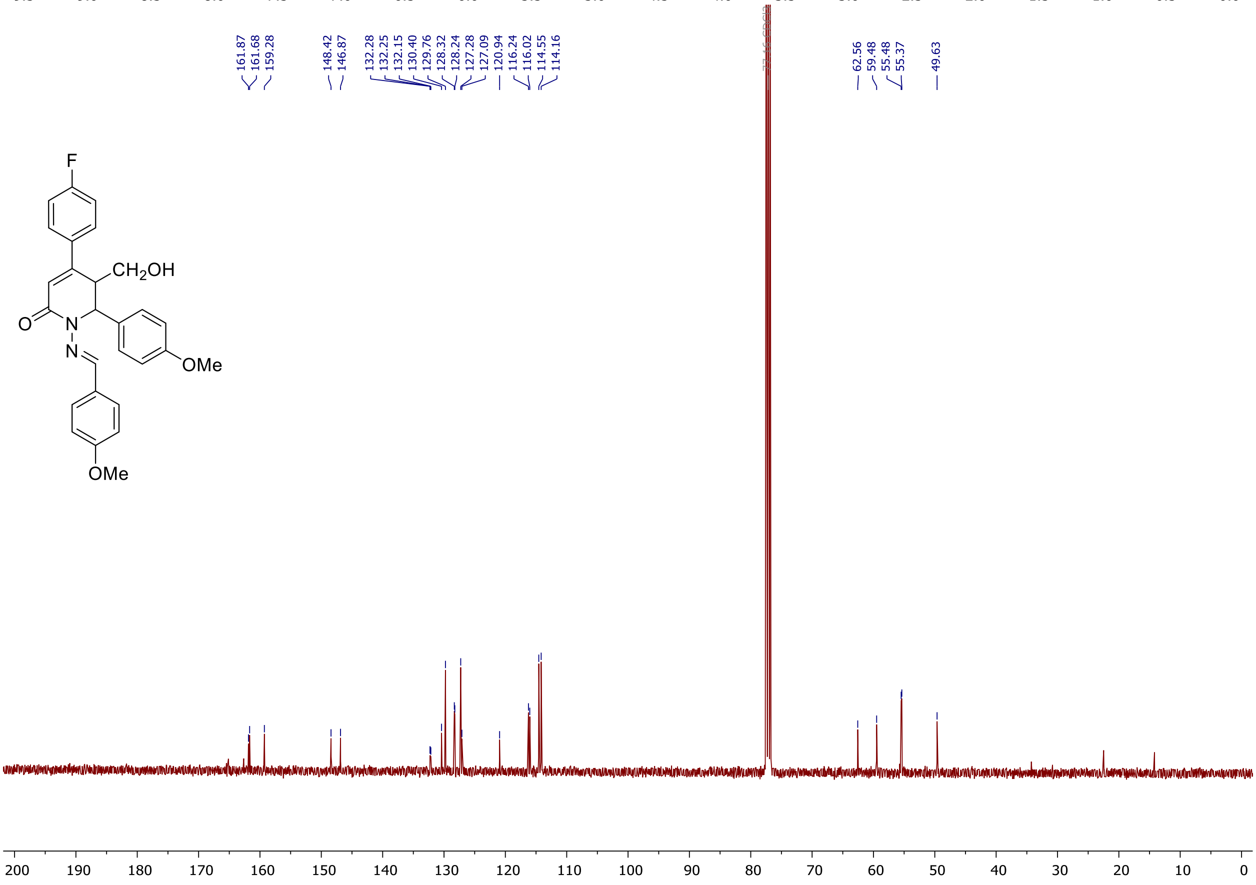

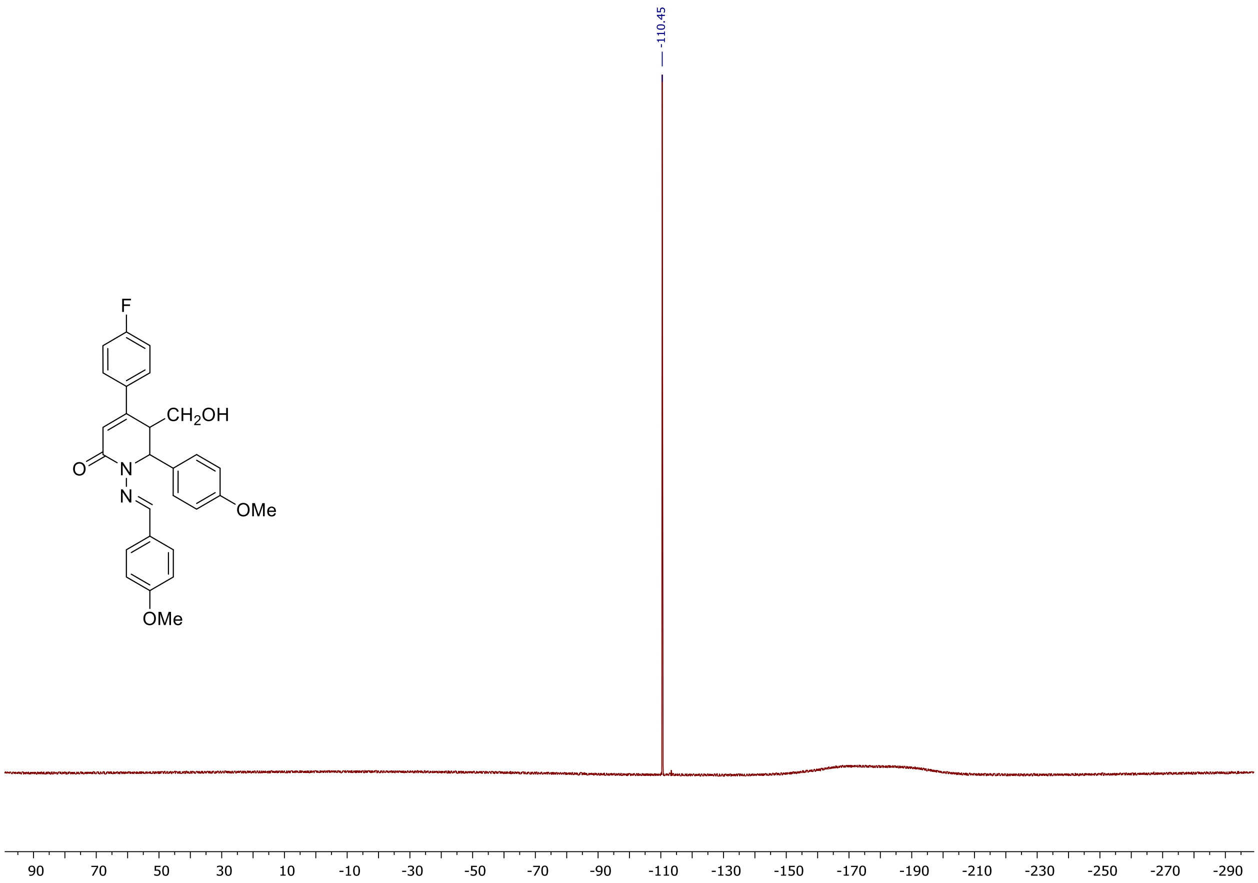
-110.45
F
CH2OH
O
N
N
OMe
OMe
90
70
50
30
10
-10
-30
-50
-70
-90
-110
-130
-150
-170
-190
-210
-230
-250
-270
-290

^{1}H, ^{13}C NMR and ^{19}F spectra of compound **6**

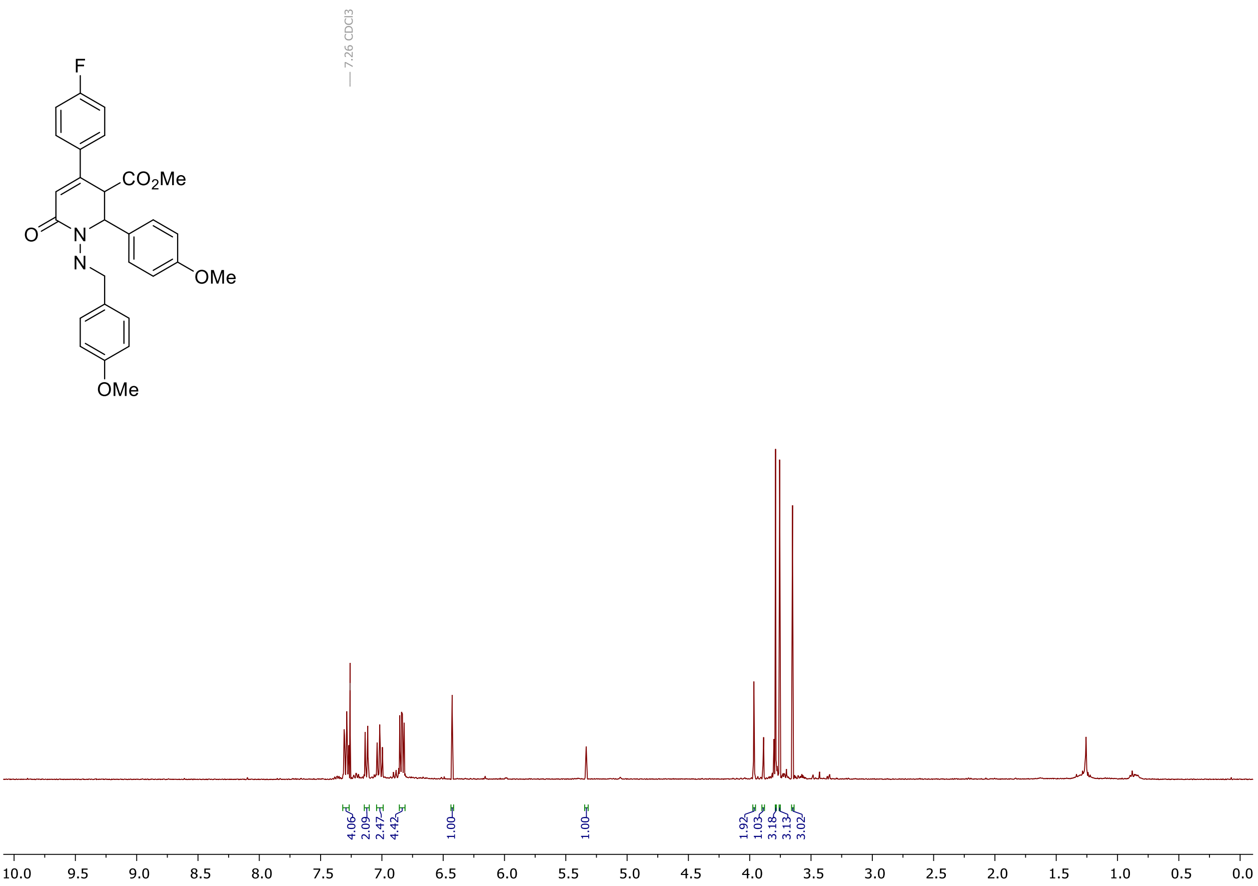

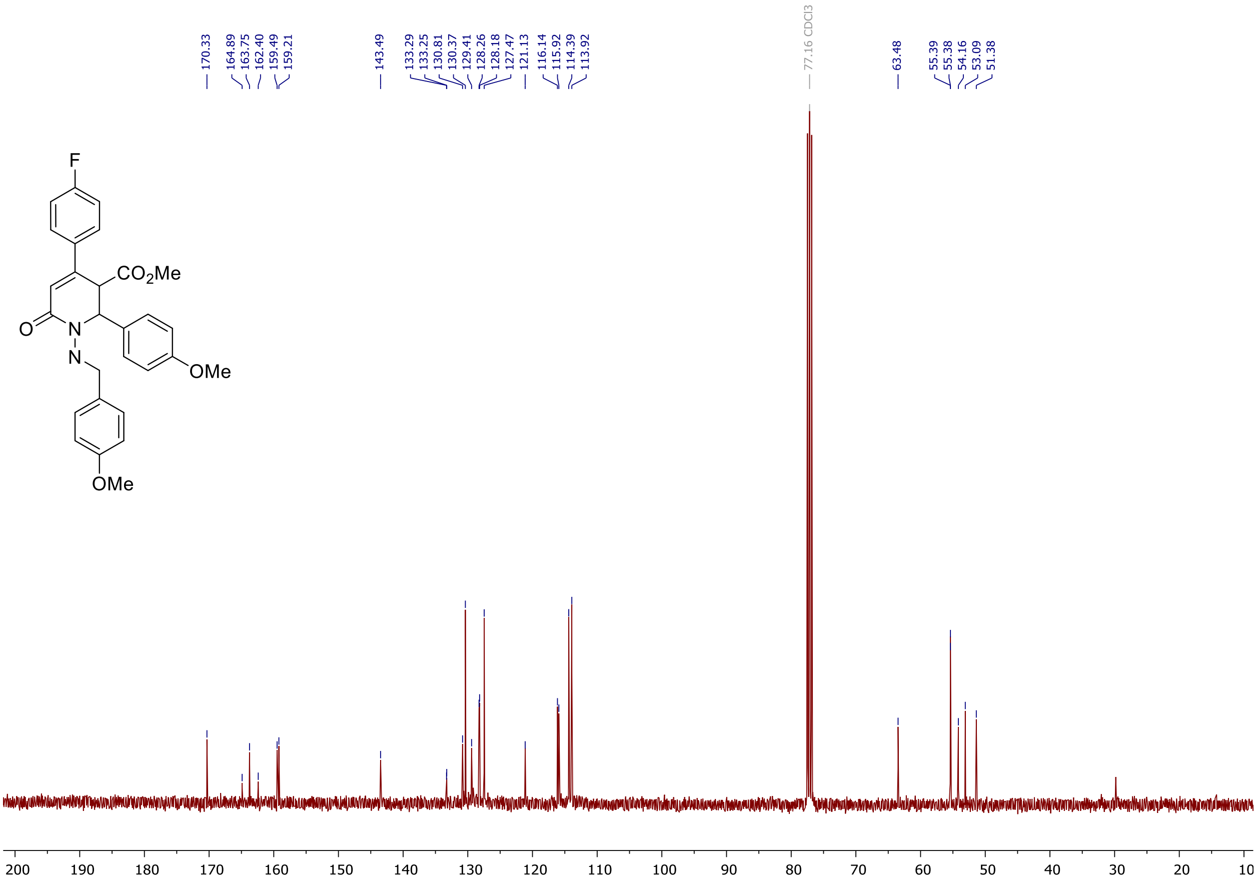

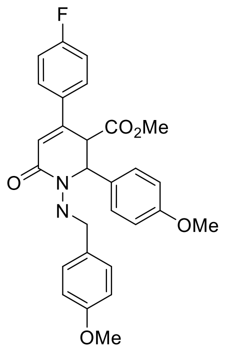

F
CO2Me
O
N
N
OMe
OMe

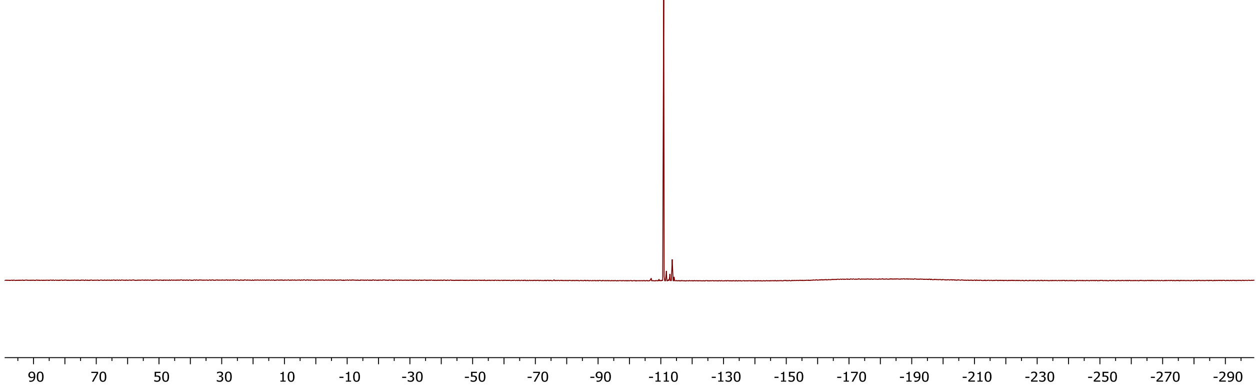

-110.94
90
70
50
30
10
-10
-30
-50
-70
-90
-110
-130
-150
-170
-190
-210
-230
-250
-270
-290

^{1}H, ^{13}C NMR and ^{19}F spectra of compound **7**

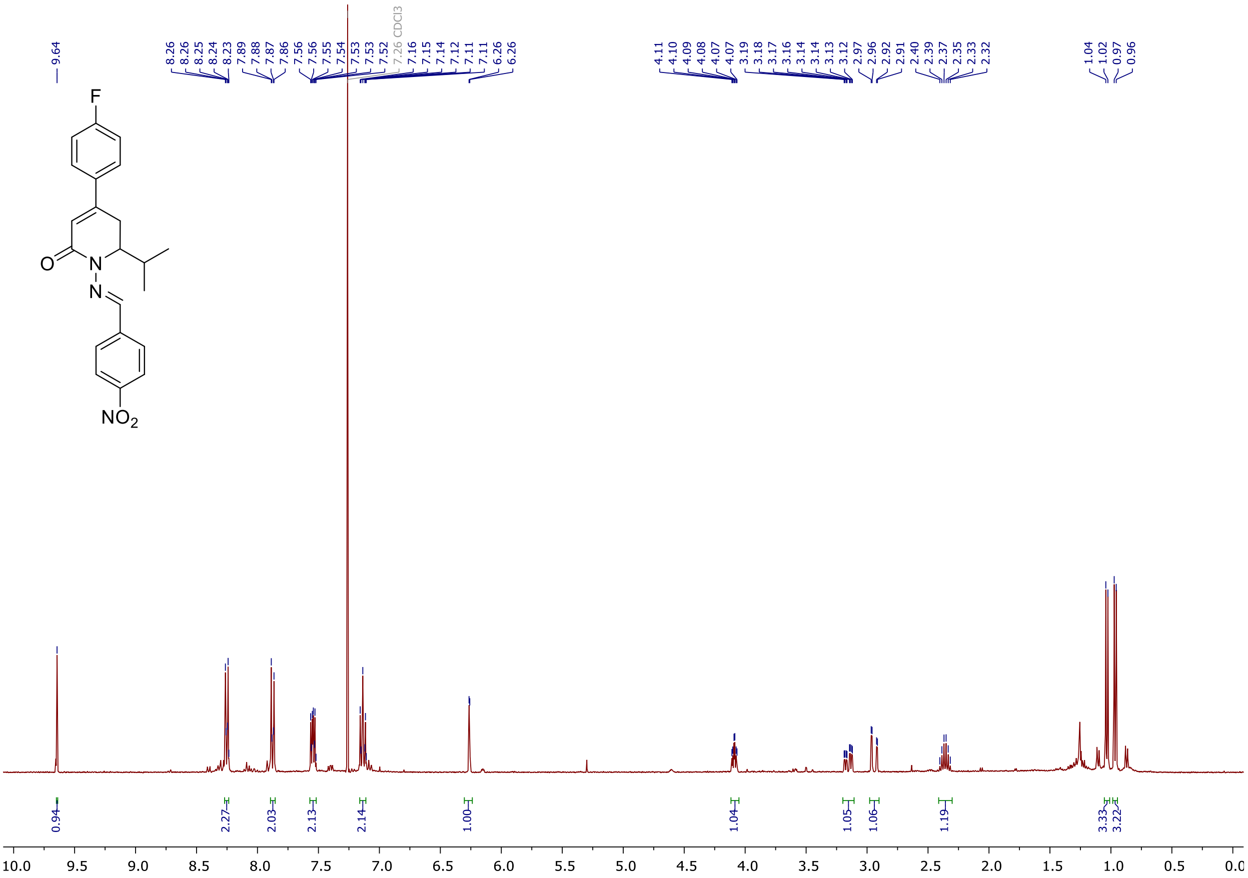

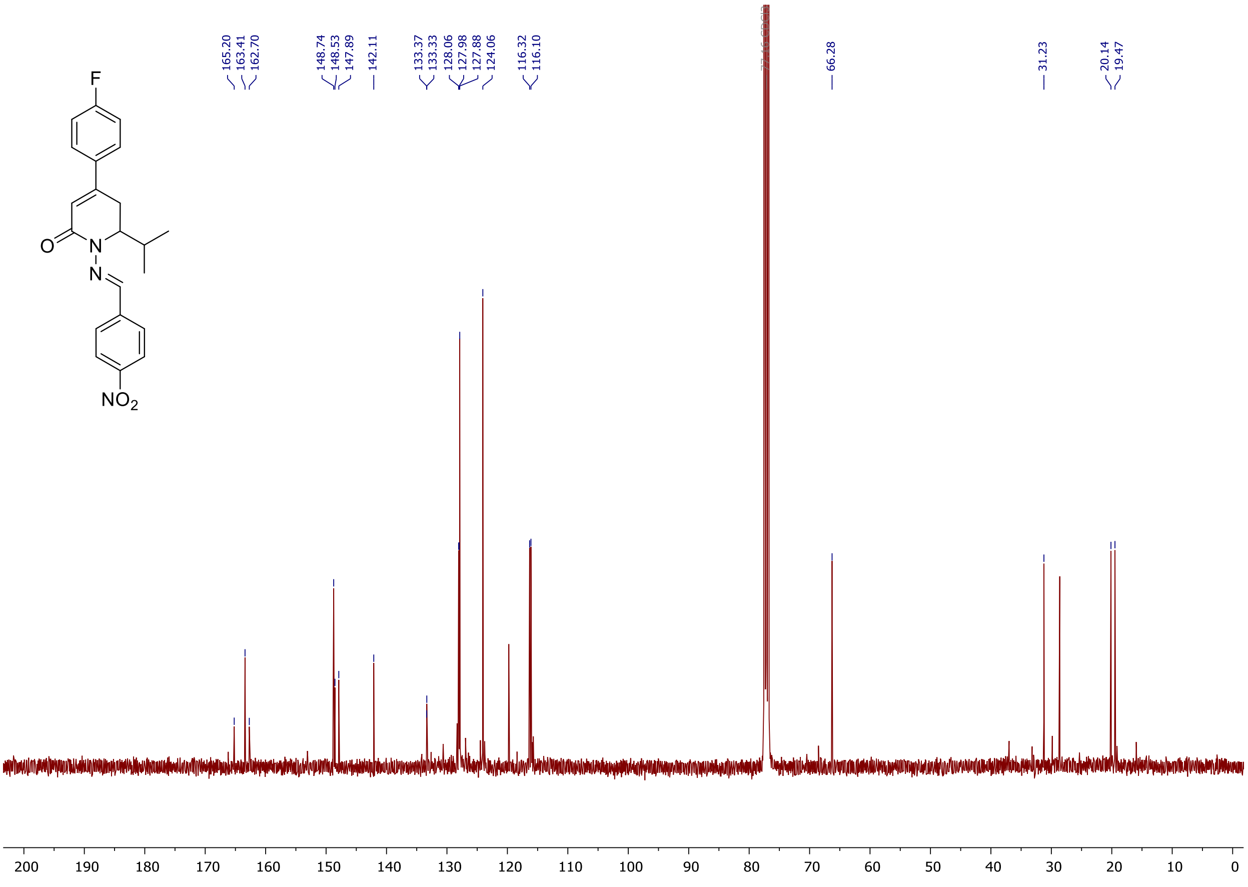

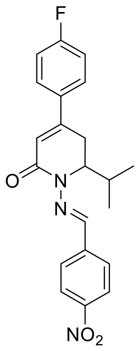
F
O
N
N
NO2

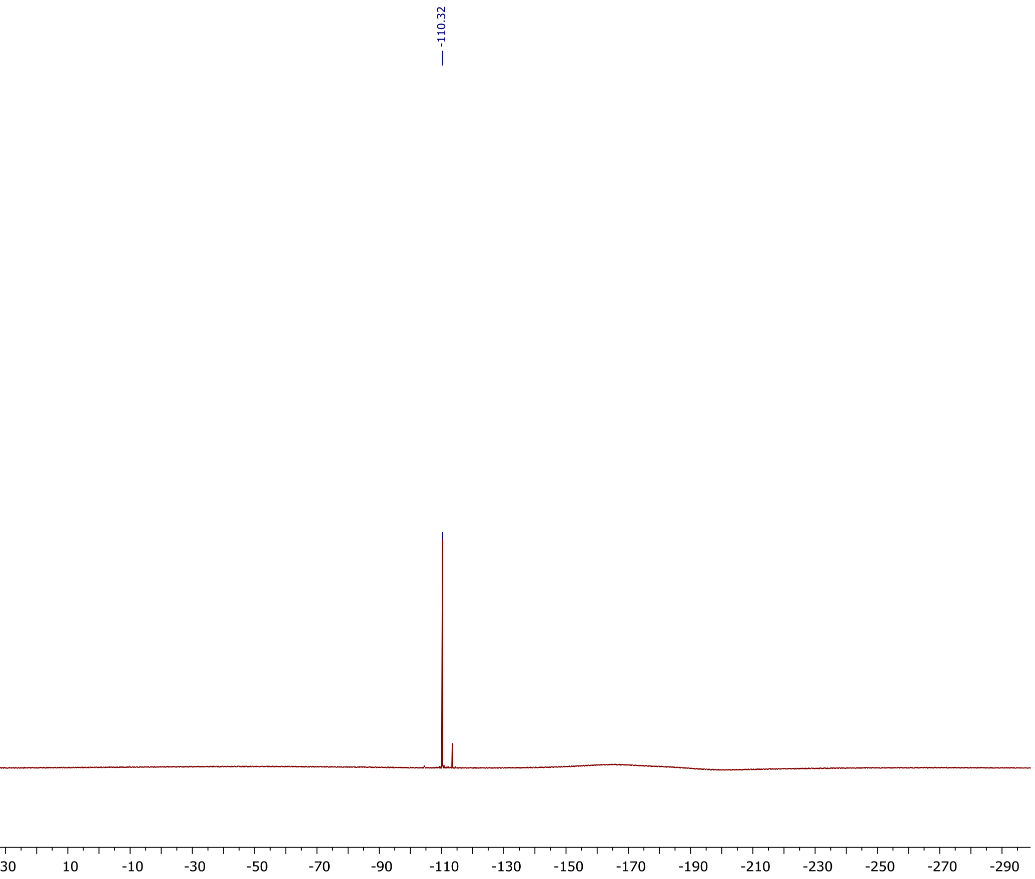
-110.32
90
70
50
30
10
-10
-30
-50
-70
-90
-110
-130
-150
-170
-190
-210
-230
-250
-270
-290

^{1}H, ^{13}C NMR and ^{19}F spectra of compound **8**

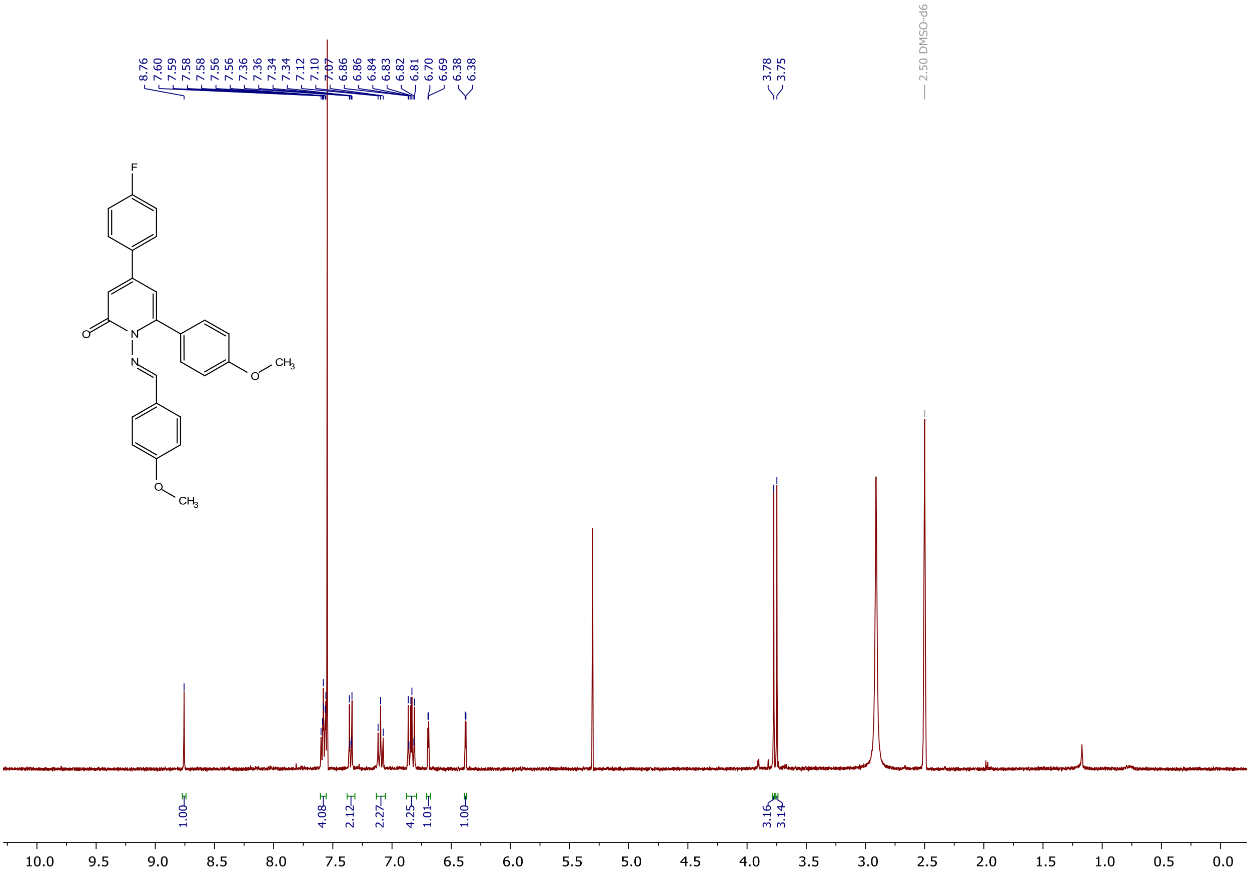

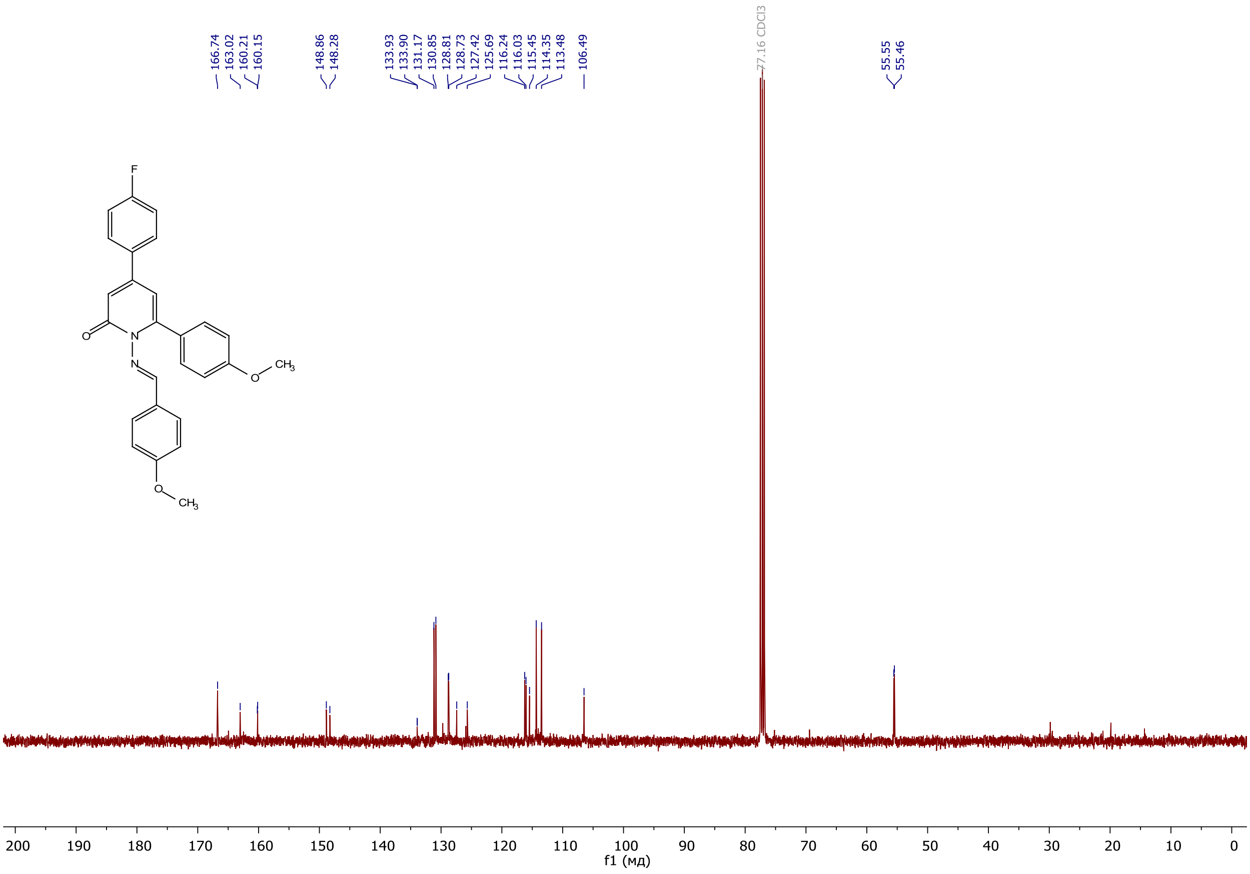

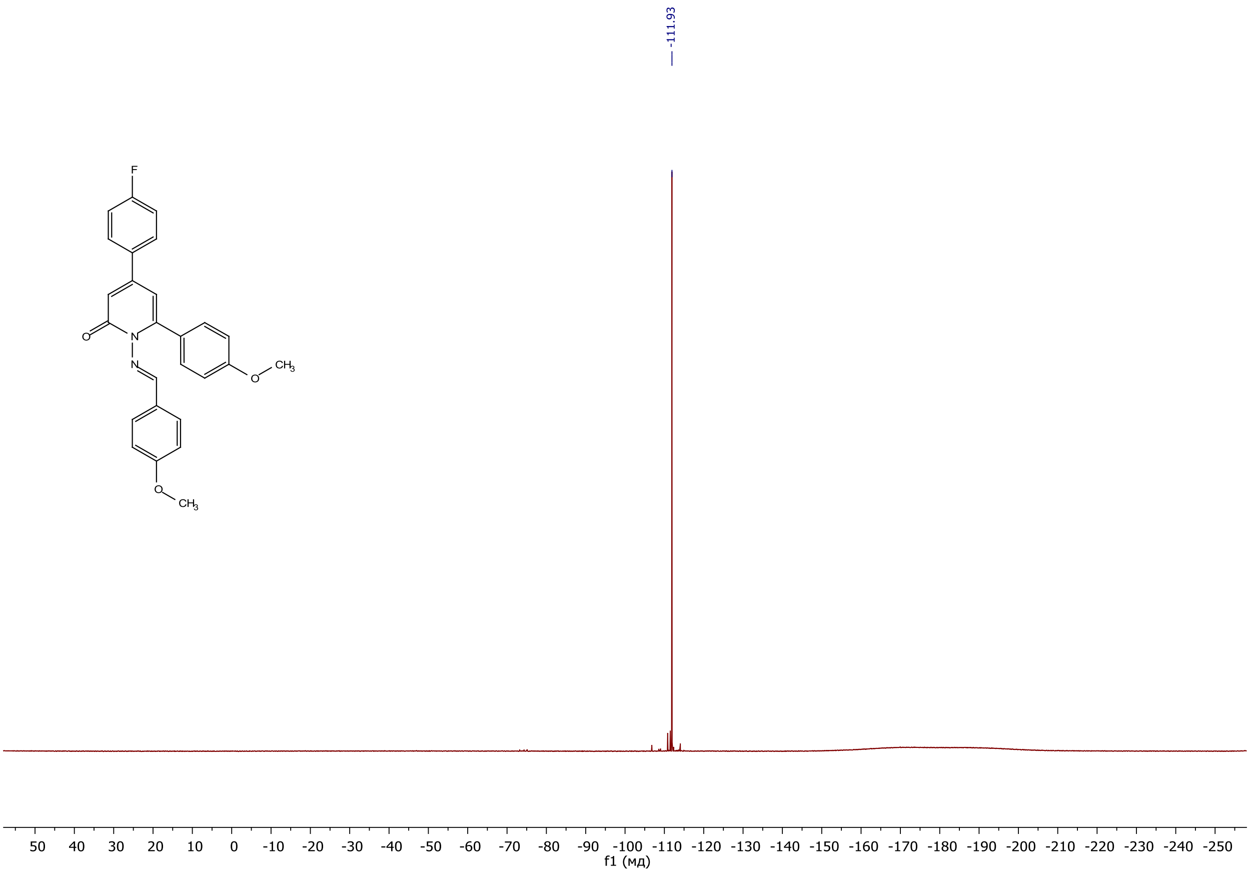
-111.93
F
O
N
N
O
CH3
O
CH3
50 40 30 20 10 0 -10 -20 -30 -40 -50 -60 -70 -80 -90 -100 -110 -120 -130 -140 -150 -160 -170 -180 -190 -200 -210 -220 -230 -240 -250
f1 (мд)

^{1}H, ^{13}C NMR and ^{19}F spectra of compound **9**

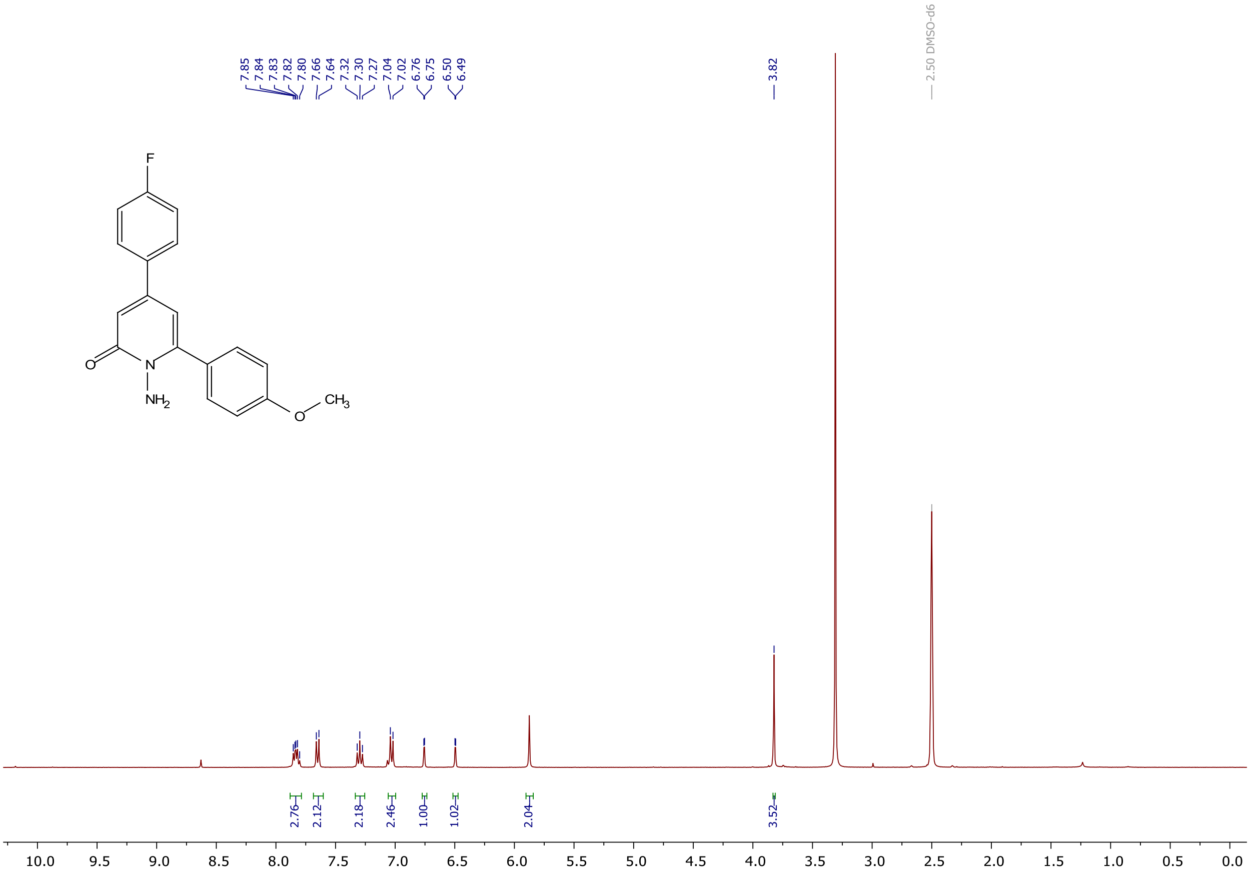

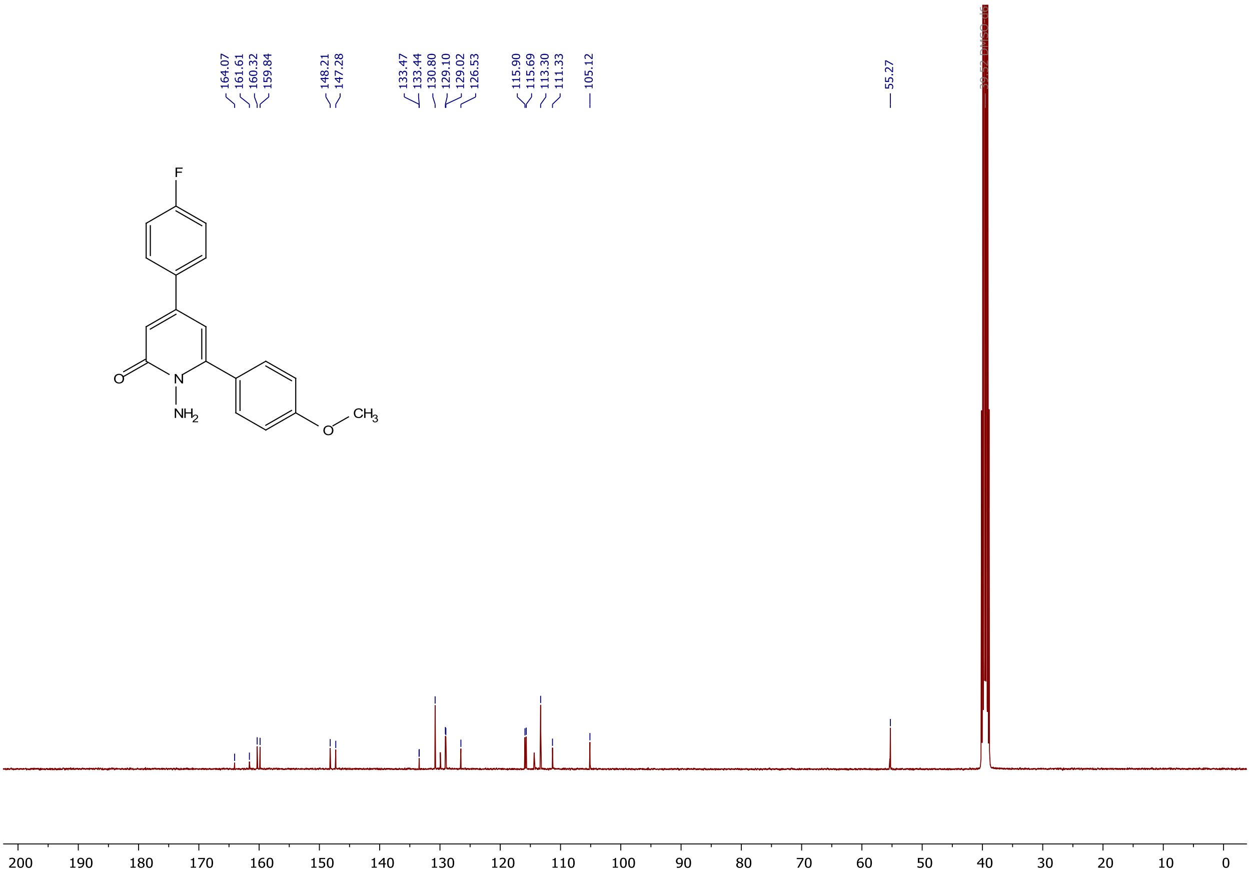

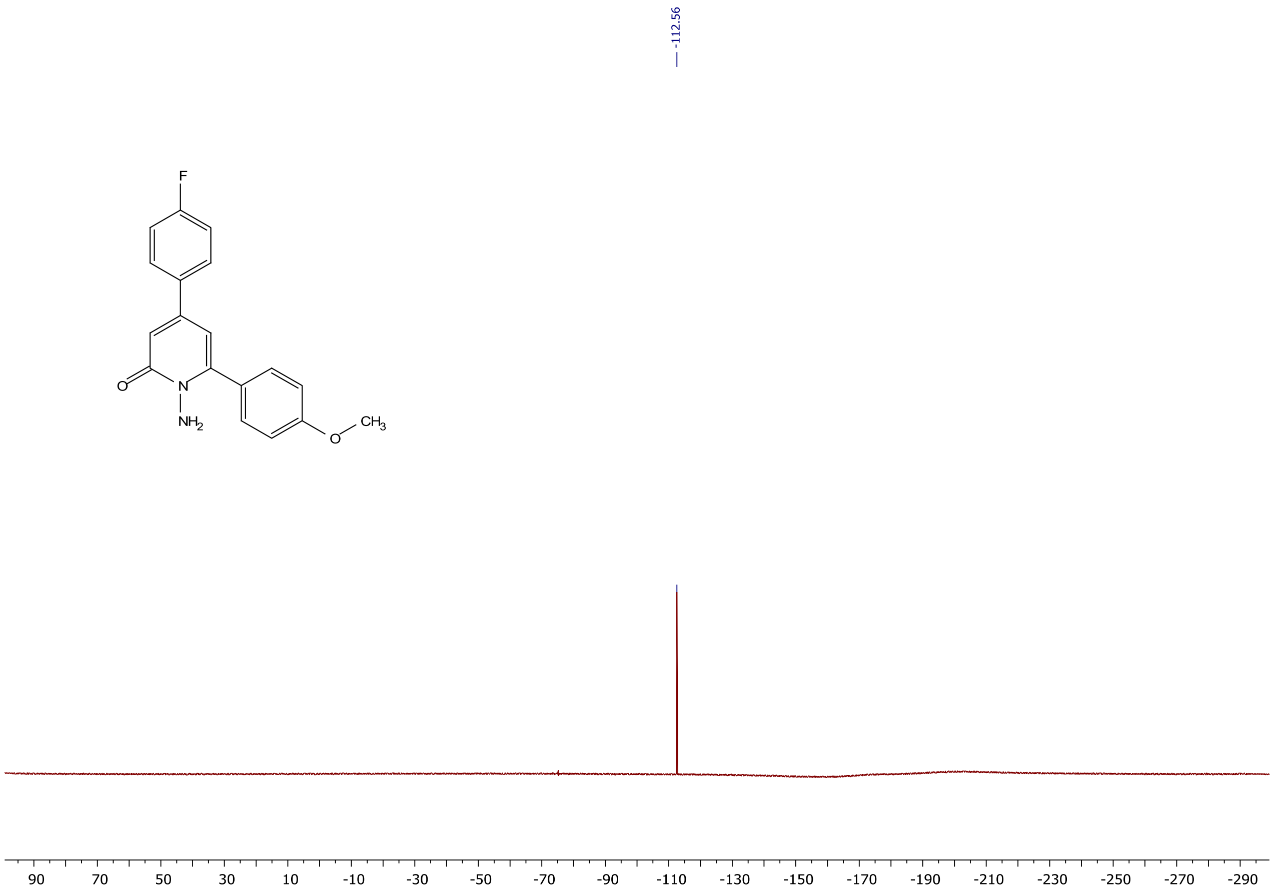
-112.56
F
O
N
NH2
O
CH3
90 70 50 30 10 -10 -30 -50 -70 -90 -110 -130 -150 -170 -190 -210 -230 -250 -270 -290
